# Characterization, phylogeny and recombination analysis of *Pedilanthus leaf curl virus*-Petunia isolate and its associated betasatellite

**DOI:** 10.1186/s12985-018-1047-y

**Published:** 2018-08-31

**Authors:** Sara Shakir, Muhammad Shah Nawaz-ul-Rehman, Muhammad Mubin, Zulfiqar Ali

**Affiliations:** 1 Virology Lab, Center for Agricultural Biochemistry and Biotechnology, University of Agriculture, Faisalabad, 38000 Pakistan; 2 Muhammad Nawaz Sharif University of Agriculture, Multan, 59220 Pakistan; 3 Present address: Boyce Thompson Institute, Ithaca, NY 14853 USA

**Keywords:** Begomovirus, Betasatellite, Phylogenetic analysis, Recombination, Infectivity analysis

## Abstract

**Background:**

Geminiviruses cause major losses to several economically important crops. *Pedilanthus leaf curl virus* (PeLCV) is a pathogenic geminivirus that appeared in the last decade and is continuously increasing its host range in Pakistan and India. This study reports the identification and characterization of PeLCV-Petunia from ornamental plants in Pakistan, as well as geographical, phylogenetic, and recombination analysis.

**Methods:**

Viral genomes and associated satellites were amplified, cloned, and sequenced from *Petunia atkinsiana* plants showing typical geminivirus infection symptoms. Virus-satellite complex was analyzed for phylogenetic and recombination pattern. Infectious clones of isolated virus and satellite molecules were constructed using a partial dimer strategy. Infectivity analysis of PeLCV alone and in combination with Digera yellow vein betasatellite (DiYVB) was performed by *Agrobacterium* infiltration of *Nicotiana benthamiana* and *Petunia atkinsiana* plants with infectious clones.

**Results:**

PeLCV, in association with DiYVB, was identified as the cause of leaf curl disease on *P. atkinsiana* plants. Sequence analysis showed that the isolated PeLCV is 96–98% identical to PeLCV from soybean, and DiYVB has 91% identity to a betasatellite identified from rose. Infectivity analysis of PeLCV alone and in combination with DiYVB, performed by *Agrobacterium* infiltration of infectious clones in *N. benthamiana* and *P. atkinsiana* plants, resulted in mild and severe disease symptoms 14 days after infiltration, respectively, demonstrating that these viruses are natural disease-causing agents. Southern blot hybridization indicated successful replication of the virus-betasatellite complex in the infected plants. Phylogenetic analysis suggests that PeLCV originated from Pakistan and later spread to India. Recombination analysis predicted that PeLCV is a donor parent for recombination and evolution of two important begomoviruses, *Papaya leaf curl virus* (PaLCuV) and *Radish leaf curl virus* (RaLCuV). The molecular phylogeny of genes encoding coat protein (*CP*) and replication associated protein (*Rep*) depict a complex evolutionary pattern of the viruses, with wide diversity in both of the genes.

**Conclusions:**

This study presents PeLCV and DiYVB as a new natural combination resulting in leaf curl disease on *P. atkinsiana* plants. Phylogenetic analysis, in addition to recent agricultural reports, identify PeLCV as an emerging broad host range Begomovirus that is resident in Pakistan and, more recently, has also spread to India. Recombination analysis showed that PeLCV was involved in a natural recombinational event leading to the evolution of two recombinant begomoviruses, RaLCuV and PaLCuV.

**Electronic supplementary material:**

The online version of this article (10.1186/s12985-018-1047-y) contains supplementary material, which is available to authorized users.

## Background

Geminiviruses (family *Geminiviridae*) are phytopathogenic viruses that cause huge crop losses every year throughout the world by infecting diverse plant species, including food and fiber crops, weeds. and ornamental plants [1]. In the past few decades, the severity and incidence of diseases caused by geminiviruses has increased tremendously. Geminiviruses having circular, small single stranded DNA (ssDNA) genome encapsidated in twinned-icosahedral particles [2, 3]. Based on differences in genome organization, mode of transmission through insect vectors, and host range, the family *Geminiviridae* is divided into nine genera [4, 5]. Among these, the genus *Begomovirus* is the largest, most wide-spread, and economically most important [6]. Begomoviruses in the Old World are usually associated with pathogenicity- and symptom-determining betasatellites, classified in the newly-established family *Tolecusatellitidae* (genus *Betasatellite*) [7]. The begomovirus-betasatellite complex not only infects several economically important crops around the world, but also weeds and ornamental plant species.

*Pedilanthus leaf curl virus* (PeLCV), a Begomovirus that was first observed in 2009 on an ornamental shrub *Pedilanthus tithymaloides*, in association with *Tobacco leaf curl betasatellite* (TbLCuB) [8], has swiftly increased its host range in Pakistan and India. The PeLCV-TbLCuB complex has also been found infecting soybean (*Glycine max*) [9], *Sesbania bispinosa* (a leguminous weed)*,* and *Raphanus sativus* (radish), showing continuous expansion in its host range [10, 11]. PeLCV has monopartite genome (single ssDNA molecule of ~ 2.8 kb), with four complementary strand genes specific for virus replication (AC1, AC3), transcription (AC2) and pathogenicity (AC4), and two virion strand genes (AV1 and AV2) for movement of virus molecules within and between plant cells.

Since begomoviruses pose a serious threat to agroecosystems worldwide [12], they have become a topic of considerable attention among molecular biologists in the agriculture sector, particularly because of their increasing host range and severity of losses to economically important crops each year [13, 14]. Since, the discovery of betasatellites in 1999, they have been found in Pakistan, India, China, Japan, Vietnam and several other South Asian and African countries. [15]. The betasatellites encode a single gene of nearly 256 nucleotides, which is responsible for symptoms induction [16, 17]. Although a lot of information is available on viruses infecting agricultural crops, comparatively less attention has been paid to the viruses that infect weeds, wild, and ornamental plant species, which are potential reservoirs of these viruses. Therefore, this study was aimed at evaluating *Petunia atkinsiana,* as an alternative host and potential reservoir for begomoviruses.

## Results

### Cloning and sequencing of viral components from *Petunia atkinsiana*

**Fig. 1:**
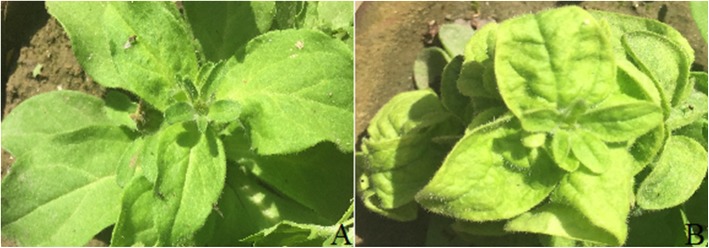
Natural infection of petunia with PeLCV. **a** Healthy petunia plant. **b** Typical PeLCV symptoms on a petunia plant. Begomovirus infection symptoms such as downward leaf curling and vein thickening can be observed in the infected plant

Begomovirus infection symptoms were observed on *P. atkinsiana* plants and leaf samples were collected from symptomatic and asymptomatic plants from two different locations, Faisalabad and Layyah districts of Punjab (Fig. 1). Full-length genomes of expected begomoviruses (~ 2.8 kb) and betasatellites (1.4 kb) were cloned (Additional file 1). Based on partial sequencing results, the clones from the Layyah and Faisalabad districts were essentially the same molecules. Therefore, for final sequencing, we decided to sequence only the clones obtained from Faisalabad. Clones were sequenced in entirety using dideoxy nucleotide chain termination sequencing. Complete sequences were submitted in the GenBank under the accession numbers MF135486 and MF135487. Sequence similarity comparison of the 2.8 kb molecule with available sequences in GenBank using BLASTn [18] identified PeLCV as the most identical known virus. The newly identified sequence was 96–98% identical to PeLCV isolated from soybean in Pakistan (Accession no: AM948961). The genome organization of PeLCV from *P. atkinsiana* consisted of four ORFs in the complementary strand and two in the virion strand, which was typical of begomoviruses.

**Table 1:** The infectivity analysis of DNA-A alone and with betasatellite

Inoculums	Plant infected/Plant inoculated (*Nicotiana benthamiana*)		Plant infected/Plant inoculated (*Petunia atkinsiana)*		Days to infection	Symptoms
	Exp 1	Exp 2	Exp 1	Exp 2		
PeLCV- [Acc # MF135486]	24/30	26/30	28/30	27/30	10–14	Stunted growth and upward leaf curling
PeLCV+DiYVB- [Acc # MF135486 + Acc # MF135487]	27/30	29/30	23/30	25/30	10–14	Downwards leaf curling, leaf swelling and vein yellowing

Most of the inoculated plants showed infection after inoculation of DNA-A alone or in combination with betasatellite

### Association of PeLCV and DiYVB with leaf curl disease in *P. atkinsiana*

The complete sequence of a ∼ 1.4 kb molecule and BLASTn analysis showed that the betasatellite associated with PeLCV is 91% identical to DiYVB, an already known betasatellite isolate from rose (Accession no: GQ478344) [19]. The sequence analysis revealed a typical genome organization of the betasatellite, i.e. the β*C1* gene, adenine rich region, and a satellite conserved region (SCR, a region of 100 nt conserved among all betasatellites) [20, 21]. DNA of infected plants was also subjected to PCR with universal primers for alphasatellites (DNA101 and DNA102) [22], but no amplification was obtained. Similarly, we tried to amplify DNA-B molecules by using universal DNA-B primers [23] but this did not result in any amplification. Thus, apparently no alphasatellites and DNA-B molecules are associated with PeLCV and DiYVB in petunia. The comprehensive analysis of the betasatellite species in association with PeLCV is presented in Additional file 2.

### *Pedilanthus leaf curl virus* shows difference in sequence similarity within the species

**Fig. 2:**
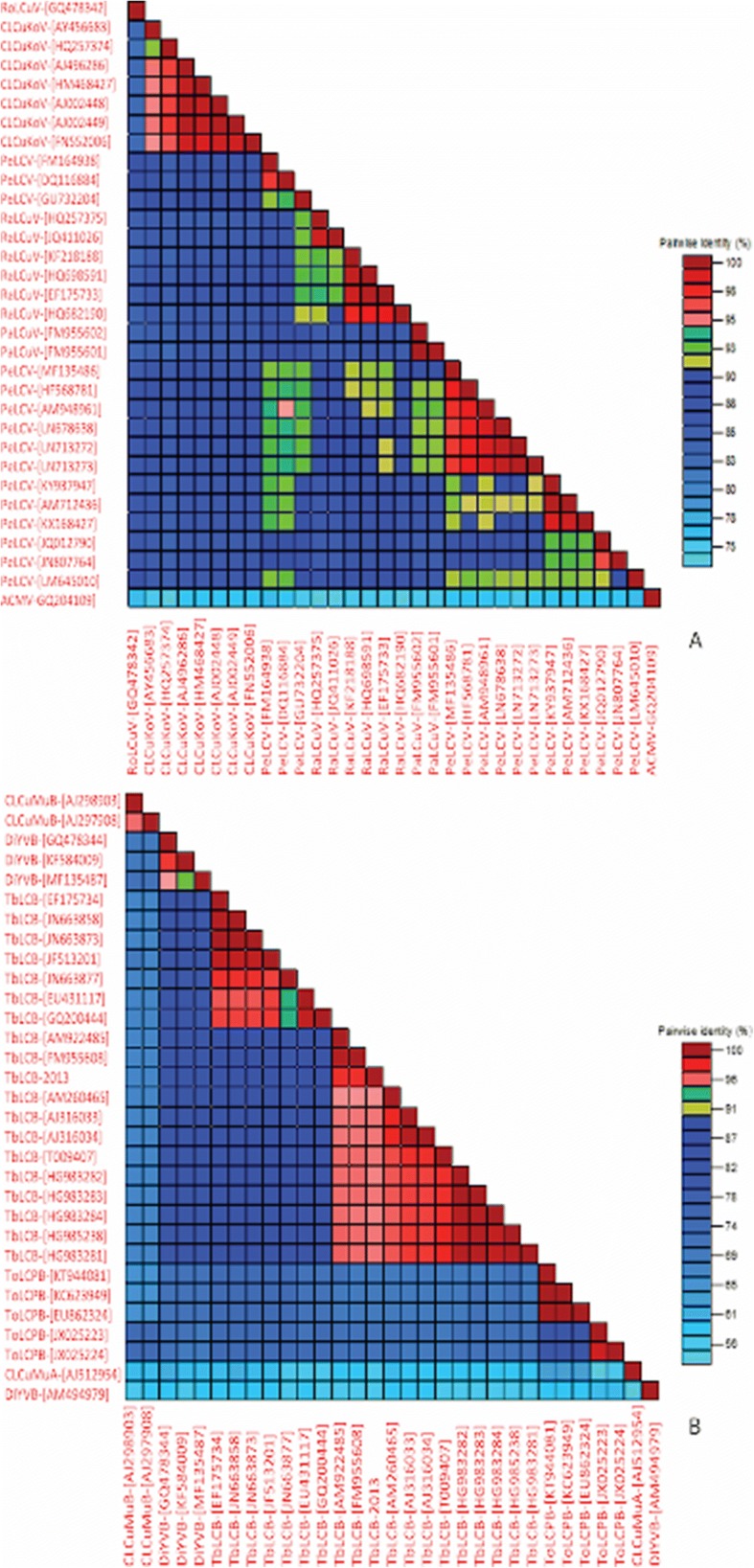
The SDT Matrix of DNA-A and betasatellites. **a** DNA-A molecules were aligned, and identity percentages were calculated using the SDT program. **b** The betasatellite sequences were also aligned by MUSCLW integrated in SDT, and the identity percentage matrix was calculated by standard procedures

**Fig. 3:**
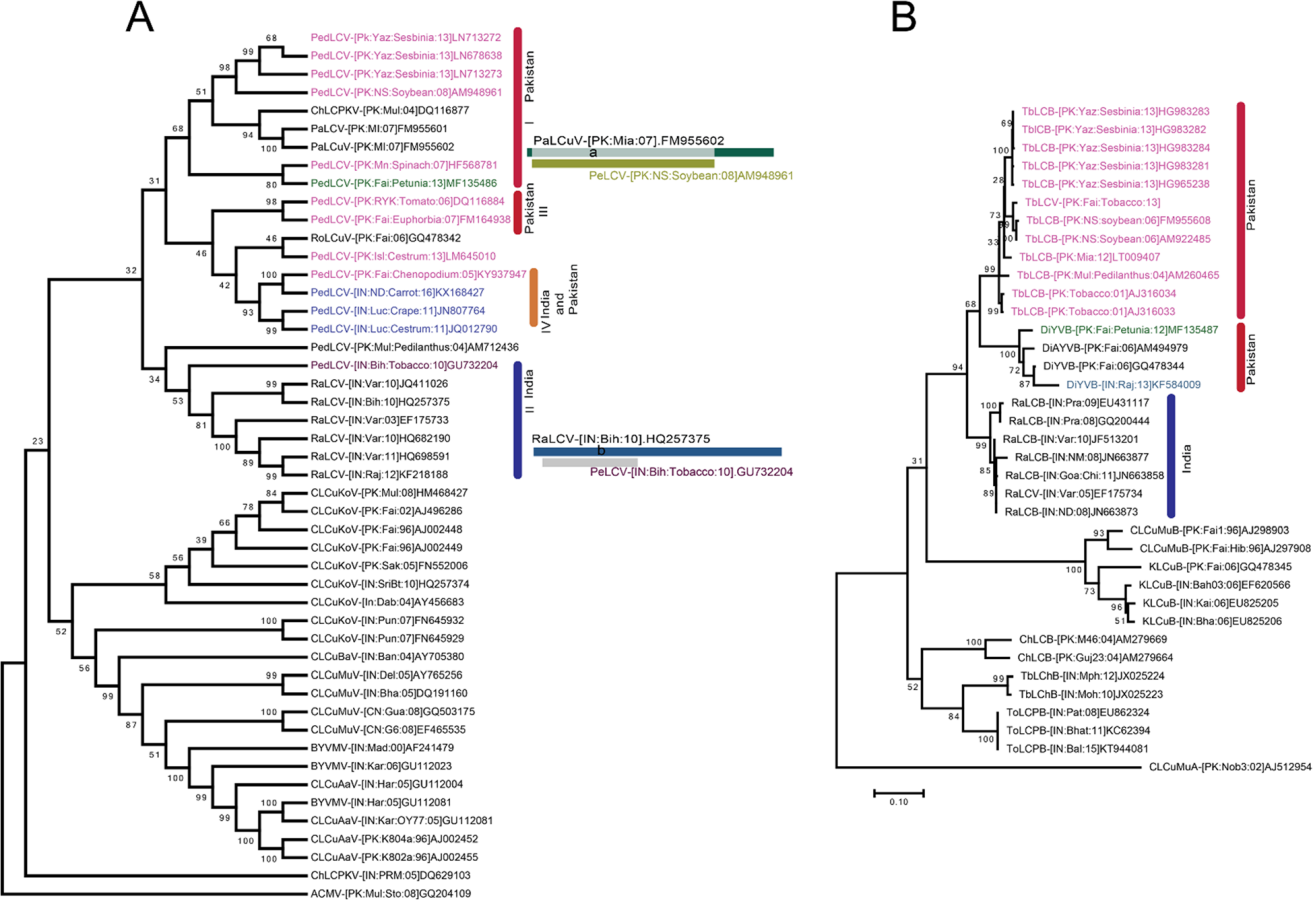
Phylogenetic trees of PeLCV and DiYVB. **a** Maximum likelihood phylogenetic tree of PeLCV was constructed based on MUSCLE alignment of selected begomovirus sequences from GenBank and sequences obtained here. *African cassava mosaic virus* (ACMV) sequence was used as an outgroup to validate the tree. The isolates of PeLCV are presented with different colors and divided into different clusters, based on their country of origin. The recombination between PaLCuV and RaLCuV was obtained through RDP4. Acronyms of viruses were taken from [24]. **b** Phylogenetic tree of DiYVB was generated by MUSCLE alignment using related betasatellite sequences from GenBank and sequence obtained here. TbLCuB isolates from India and Pakistan constitute separate groups, whereas the DiYVB cluster is dominated by isolates from Pakistan. The MF135487 isolate was identified from petunia in this study, while KY937948 was isolated from *Chenopodium*, in combination with PeLCV. Cotton leaf curl Multan alphasatellite was taken as outgroup. The numbers at each node represent the percentage of 1000 bootstrap values

According to the begomoviruses species criteria, 91% sequence identity is considered as a cut-off value for a new begomovirus and betasatellite species [7, 24]. The nucleotide identity percentage with other begomoviruses revealed that PeLCV is closely related with *Papaya leaf curl virus* (PaLCuV), *Radish leaf curl virus* (RaLCuV), and *Cotton leaf curl Kokhran virus* (CLCuKoV). The PaLCuV isolates have greatest sequence similarity (92–93%) with PeLCV isolates from *Sesbania*, petunia, radish, spinach, and soybean (Fig. 2) [8–11]. Similarly, RaLCuV was highly similar to PeLCV isolates from *Sesbania*, petunia, tobacco, and spinach. This shows that PaLCuV and RaLCuV may have originated from PeLCV through recombination (Fig. 3). From Fig. 2, it is clear that CLCuKoV is the most closely related begomovirus to PeLCV in the region, with more than 85% similarity to the PeLCV isolates.

### Molecular phylogeny of PeLCV and associated betasatellites

The phylogenetic analysis of PeLCV revealed different clusters of the virus, along with their recombinants. PeLCV isolates from soybean, petunia, spinach and *Sesbania* constitute a monophyletic group (Fig. 3a). A separate branch of PaLCuV radiating from PeLCV was interesting. The recombination analysis revealed that PaLCuV is a recombinant derived from PeLCV and an unknown virus. The majority of virion and complementary strand genes were derived from PeLCV, which justifies its position in the phylogenetic tree (Fig. 3). Similarly, RaLCuV (originally from India) was also found to be a recombinant of PeLCV and an unknown virus [25]. However, the nucleotide span was limited to the virion (AV2 and coat protein genes) strand genes. The PeLCV isolates from tomato, euphorbia, *Cestrum*, carrot, crape and *Chenopodium* constituted a separate group. With the available data, there was no detectable level of recombination among these isolates. In the phylogenetic tree, four different clusters are evident, where cluster-I represents the PeLCV isolates originating from Pakistan, whereas cluster-II represents the isolates from India. An isolate from cluster-II appeared to be a donor virus for the RaLCuV from India. Cluster-III represents the PeLCV isolates from Pakistan reported from tomato and euphorbia plants. In the fourth cluster, the PeLCV isolates from crape and *Cestrum* make up a separate group. However, the other three isolates represent a separate group of Indian and Pakistani isolates.

The phylogenetic tree based on betasatellites is presented in Fig. 3b. The phylogenetic tree indicated that the majority of the betasatellite isolates associated with PeLCV belong to a single monophyletic group of TbLCuB originating from Pakistan. On the other hand, a monophyletic group of TbLCuB isolates from India constituted a separate clade in phylogenetic tree. However, in the present study, DiYVB was isolated in association with PeLCV, which made a monophyletic group with other isolates of DiYVB. There are only four sequences of DiYVB in GenBank and three of them were reported from Pakistan. Interestingly, the single isolate of DiYVB from India was in a close cluster with the molecules from Pakistan. Although, Cotton leaf curl Multan betasatellite (CLCuMuB), Tobacco leaf curl chlorosis betasatellite (TbLChB) and Tomato leaf curl Patna betasatellites (ToLCPaB) have also been associated with PeLCV, they constitute separate clusters due to lower sequence identity with TbLCuB or DiYVB.

### PeLCV shows differential evolutionary pattern for coat protein and rep protein

**Fig. 4:**
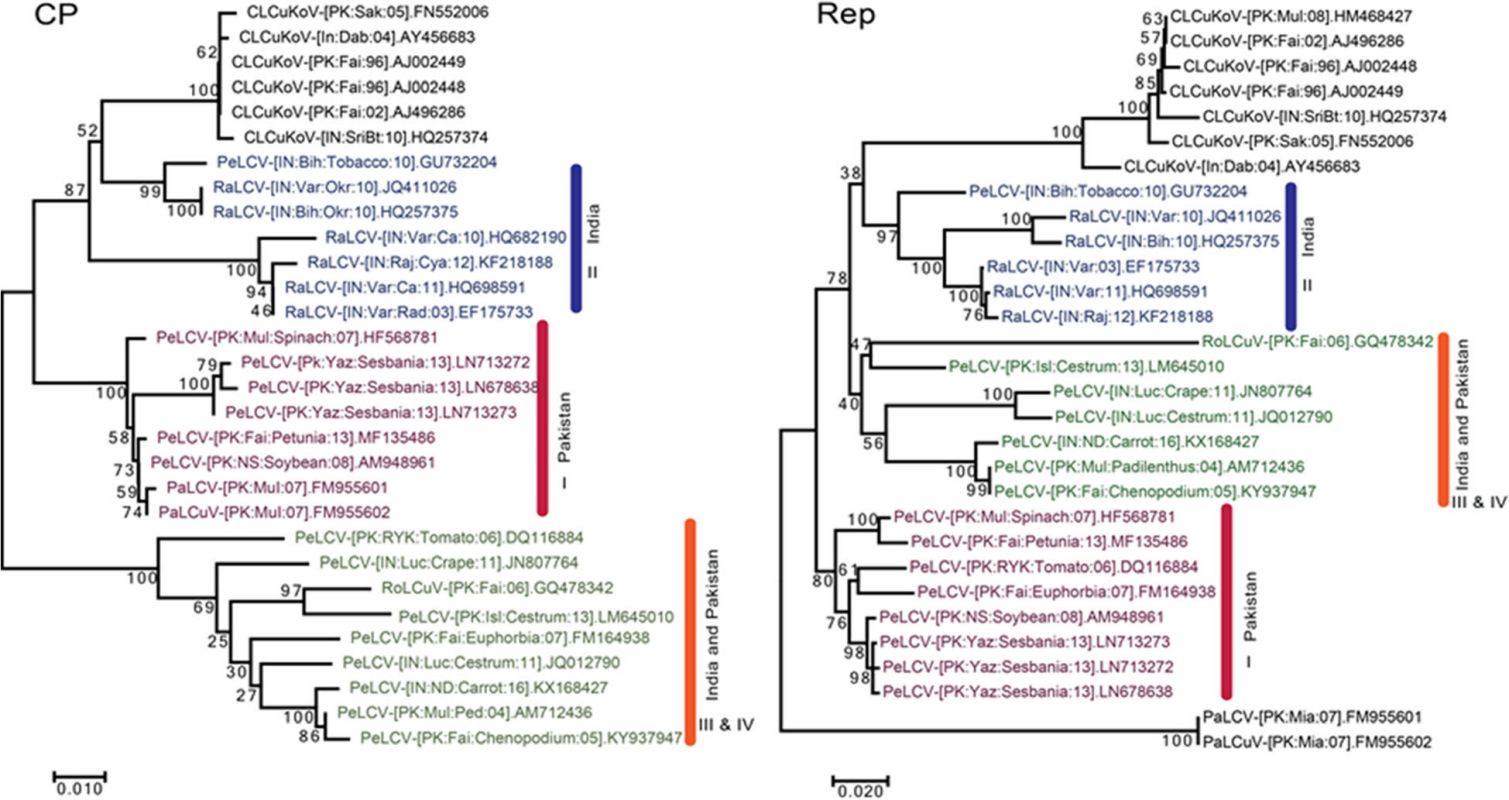
Molecular phylogeny of the *CP* and *Rep* genes: The predicted *CP* genes (left panel) and *Rep* gene sequences (right panel) were used to construct phylogenetic trees. Both genes display the huge molecular diversity of PeLCV. Different clusters have been demarcated according to Fig. 3. The numbers at nodes represent 1000 bootstrap replication values

The phylogenetic trees constructed using the coat protein (*CP*) and Replication associated (*Rep*) genes of PeLCV and closely related viruses resulted in a complex pattern (Fig. 4). With the exception of CLCuKoV and RaLCuV, none of the *CP* or *Rep* genes segregated according to their cognate genes. The close clustering of CLCuKoV for *CP* and their cognate *Rep* gene (collectively spanning 70% of the viral genome) suggested that the RaLCuV and PeLCV isolates (PeLCV-[IN:Bih:Tobacco:10]) in India are closely related to CLCuKoV (Fig. 4, Cluster-II). In contrast, the *CP* gene of Cluster I isolates from Pakistan (Fig. 4a) segregated differently than their cognate *Rep* genes. A similar pattern of differential segregation was observed for *CP* of the cluster-III and IV isolates. The *Rep* gene of PaLCuV segregated differently than its cognate *CP* gene and made an outgroup in the phylogenetic tree (Fig. 4). These observations further support the hypothesis of recombination between PeLCV and a virus of unknown origin.

### Infectivity analysis of PeLCV with DiYVB

**Fig. 5:**
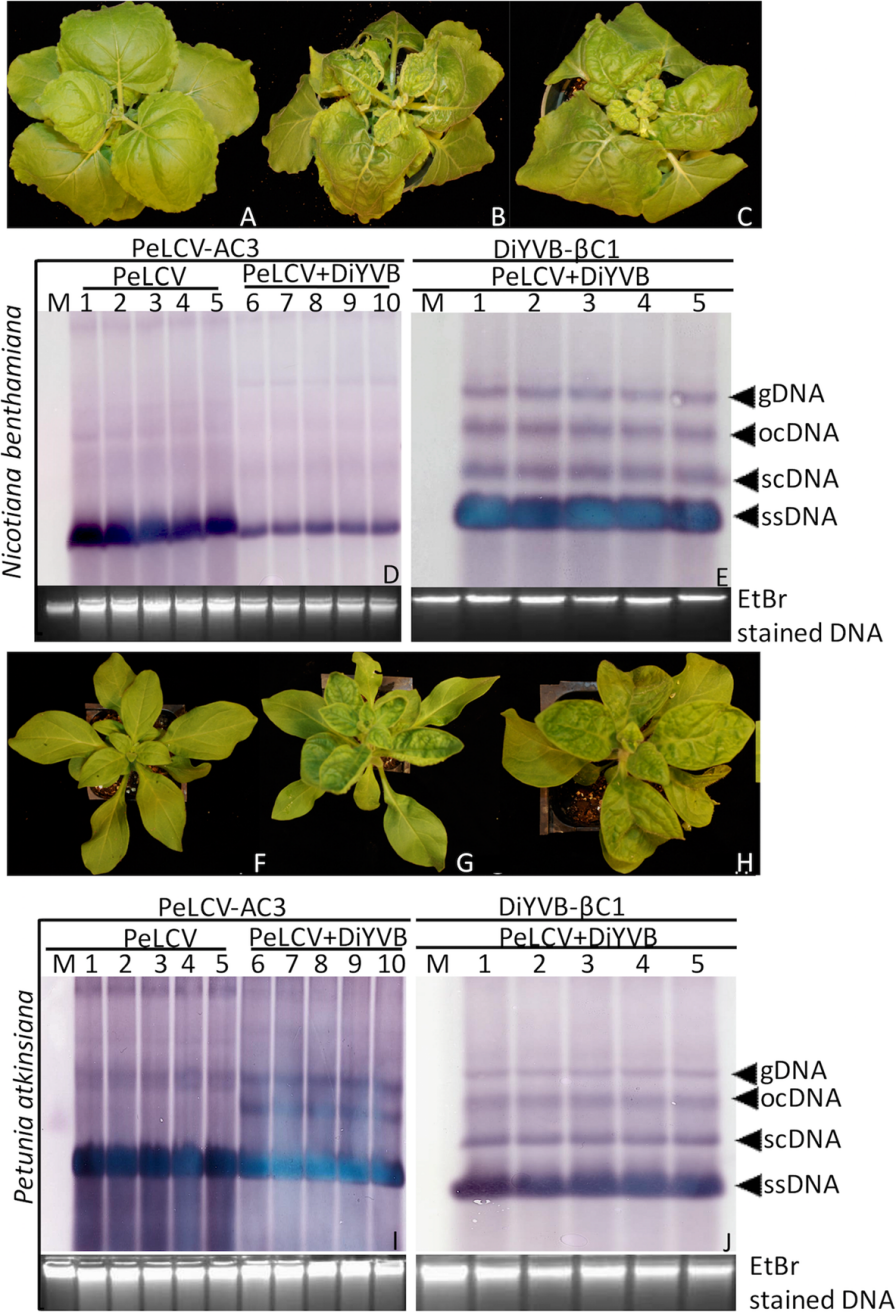
Infectivity analysis of PeLCV alone and with DiYVB partial dimeric constructs in *Nicotiana benthamiana and petunia atkinsiana*. Inoculation of PeLCV alone and in combination with DiYVB resulted in severe symptoms after 14 days. **a** *Nicotiana benthamiana* plants were inoculated with an empty pCambia2300 plasmid. **b** *Nicotiana benthamiana* plants inoculated with PeLCV alone. **c** *Nicotiana benthamiana* plants inoculated with PeLCV and its cognate DiYVB resulted in severe disease symptoms including downward leaf curling, leaf swelling and vein yellowing. Plants were photographed at 14 dpi. **d** Detection of PeLCV replication in *N. benthamiana* plants by Southern blot hybridization. DNA of five different plants infiltrated with PeLCV alone (lane 1–5), and PeLCV along with DiYVB (lane 6–10) were probed with *REn* (AC3) gene. **e** DNA of five different plants infiltrated with PeLCV along with DiYVB (lane 1–5) were probed with *βC1* gene. M represents the mock inoculated plant DNA in both gel blots. Panel **f** represents the control petunia plant, inoculated with empty plasmid pCambia2300. The agroinfiltration of PeLCV alone resulted in mild symptoms on petunia plants (panel **g**), while, addition of DiYVB resulted in severe symptoms on petunia plants (panel **h**). The Southern blotting confirmed the replication of both DNA-A and betasatellite (panels **i** and **j**) in the agro-inoculated plants. The similar probes were used as described for panels **d** and **e**. The bottom panels represent the ethidium bromide (EtBr) stained DNA as a loading control

*Agrobacterium*-mediated inoculation of *N. benthamiana* leaves with an infectious construct of PeLCV resulted in upward leaf curling and stunted growth at 14 dpi as compared to mock inoculated plants (Table 1 and Fig. 5). These pathogenic symptoms along with replication of the viral DNA in the host plant, were confirmed by southern blot analysis (Fig. 5), indicated that PeLCV alone can cause infection in *N. benthamiana* plants. However, co-inoculation of this host with partial dimeric constructs of PeLCV and its cognate DiYVB resulted in more severe downwards curling and swelling of leaves and vein yellowing (Fig. 5), suggesting that DiYVB has an additive role in disease development. Agro-inoculation of the original host, *P. atkinsiana* plants with PeLCV alone and in combination with DiYVB resulted in similar phenotype as observed in *N. benthamiana* (Fig. 5). In both hosts, efficient replication of betasatellite by its helper virus was confirmed by Southern blotting (Fig. 5). However, the observations that DiYVB does not increase the accumulation of viral DNA in either *N. benthamiana* or *P. atkinsiana* plants (Fig. 5), while exacerbating the virus symptoms suggest that there may be another mechanism through which betasatellite is helping virus during its infection cycle. One possible mechanism is that DiYVB affect the viral movement in the host plant, but it is remained to be further studied.

## Discussion

In the present study, phylogenetic, recombination, and infectivity analysis of an important begomovirus, *Pedilanthus leaf curl virus* was performed. The ornamental plant *P. atkinsiana* was found to be infected with this virus. *Petunia atkinsiana* is grown all over the world due to its attractive flower and leaf shape. Based on high nucleotide identity (96–98%) with the previously reported soybean strain of PeLCV, we considered the isolated virus to be a variant of PeLCV. Previously, PeLCV was found in combination with TbLCuB, but in the current study it was associated with DiYVB. Therefore, it can be considered as a new natural combination of a begomovirus with a betasatellite.

The literature analysis of PeLCV identified it as a very interesting virus. The data suggested that PeLCV infects at least 15 different hosts (Additional file 2), the majority of which are from Pakistan. Therefore, it can be predicted that PeLCV originated in Pakistan. Although, several begomoviruses have been reported to infect a single host, only few begomoviruses that infect multiple hosts have been identified yet [26, 27]. In case of RNA viruses, a single virus like *Cucumber mosaic virus* (CMV) has been shown to infect multiple hosts [28]. However, for begomoviruses, such a broad host range has rarely been observed. To the best of our knowledge, PeLCV is among the begomoviruses with the broadest host ranges.

RaLCuV is only reported from India, and in the phylogenetic trees of *CP* and *Rep* genes, it appeared to be closely related to CLCuKoV. CLCuKoV is a highly recombination-prone virus and is considered to be major factor in the evolution of cotton leaf curl disease [29]. From the current data, it can be considered that RaLCuV in India might have evolved as a recombinant virus with high nucleotide similarity to PeLCV or CLCuKoV. Although experimental evidence will be needed to confirm the origin of RaLCuV, it can be hypothesized that RaLCuV evolved due to positive selection for point mutations in CLCuKoV. In recent studies, there is an agreement that the majority of the *Rep* genes of begomoviruses are more variable than *CP* [30]. However, in the case of PeLCV, the genetic variation is equally distributed in both the genes.

The recombination analysis of closely related begomoviruses revealed that PaLCuV in Pakistan possibly originated from PeLCV. Molecular phylogeny of DNA-A indicated that viral isolates of PeLCV originating from Pakistan are different from Indian isolates and constitute separate clades (with the exception of PeLCV-[PK:Fai:Chenopodium:05] and PeLCV-[PK:Mul:Pedilanthus:04]). Therefore, it can be concluded that PeLCV originated from Pakistan and then later spread to India. However, it is surprising that PeLCV is associated with different betasatellites in India and Pakistan. In Pakistan, PeLCV is associated with Tobacco leaf curl betasatellite (TbLCuB), Cotton leaf curl Multan betasatellite (CLCuMuB), or Digera yellow vein betasatellite (DiYVB). In contrast, in India it is associated with DiYVB, ToLCPaB and TbLChB. The complexity of betasatellites can be observed in the phylogenetic tree. Unlike, DNA-A component, the betasatellites (TbLCuB) from both the countries constitute separate groups. The Indian isolates of TbLCuB have been named as Radish leaf curl betasatellite in GenBank, but according to the species criteria of betasatellites from ICTV, they are more than 91% similar to Pakistani isolates of TbLCuB. Therefore, we have renamed them as TbLCuB. The separate group of TbLCuB from India suggests that the TbLCuB present in Pakistan has not yet spread to India. The majority of the DiYVB isolates were found in Pakistan. However, in a single incidence, DiYVB is reported from India, suggesting the possible recent introduction of this particular betasatellite from Pakistan to India. These observations indicate that begomoviruses and their satellites are spreading into different territories.

Although, PeLCV has been reported from several different hosts, we conducted the first infectivity experiments with *N. benthamiana* and *P. atkinsiana*. Successful infection in *N. benthamiana* and the original host, *P. atkinsiana* plants inoculated with partial dimeric constructs of PeLCV and its associated DiYVB proved them to be the causative agents for leaf curl disease. Moreover, plants inoculated with PeLCV alone showed upward curling and swelling of leaves, whereas, more severe symptoms were observed in the host plants with the inoculation of PeLCV along with its cognate betasatellite, DiYVB. These results suggest that PeLCV alone is sufficient to cause infection but addition of betasatellite increases the virus pathogenicity.

## Conclusions

We have characterized *Pedilanthus leaf curl virus* (PeLCV) and *Digera yellow vein betasatellite* (DiYVB) as a new natural combination associated with leaf curl disease on petunia plants. Through the phylogeny of PeLCV, we found complex evolutionary pattern of the virus, with great diversity in genes encoding coat protein and replication associated protein. Phylogenetic analysis predicted that PeLCV is closely related to RaLCuV and PaLCuV and recombination analysis demonstrated that PeLCV was involved in a natural recombination event and is the donor parent for the evolution of two recombinant virus species, RaLCuV and PaLCuV. Infectivity analysis with infectious dimeric constructs of PeLCV and DiYVB reproduced leaf curl disease symptoms in *N. benthamiana* and *P. atkinsiana* plants, proving them as natural disease-causing agents. Recent reports of PeLCV infection on different plant species suggests that it is an emerging Begomovirus that has spread to other weed and vegetable plants within last few years, as well as crossing the continental barriers from Pakistan to India. Moreover, PeLCV showed relaxed trans-replication with five different betasatellite species, make it a particularly interesting begomovirus. High genetic variability in the virus suggests that it is evolving at an alarming rate. The natural existence of broad host ranges and the recombination propensity of different betasatellites may result in a future epidemic of this virus. Therefore, this study highlights the importance and flexibility of PeLCV in terms of infection and spread to other plant species.

## Methods

### Virus source and DNA extraction

During a survey in 2013–2014, *P. atkinsiana* plants were found naturally infected in the field, based on typical symptoms of begomoviruses like leaf curling and vein swelling. Leaf samples from both symptomatic and asymptomatic *P. atkinsiana* plants were collected from Faisalabad and Layyah districts of Punjab, Pakistan. Surrounding regions were also investigated for begomovirus infection on petunia plants, but no significant infection was observed. Leaf samples were labelled and stored in − 80 °C until DNA extraction. DNA was extracted from 100 mg of leaf tissues using the cetyl trimethylammonium bromide (CTAB) method [31] and used as source material to analyze begomovirus infection.

### Rolling circle amplification, cloning and sequencing of begomovirus and betasatellite

To amplify circular molecules from DNA of petunia plants, rolling circle amplification (RCA) was performed following the manufacturer’s protocol (TempliPhiTM, GE Healthcare), as it is most reliable method to look into the diversity of begomoviruses in the infected samples [32]. Briefly, 20 ng of DNA was diluted in 13ul of Phi mixture (100 μM hexamer primers, 10 mM dNTPs and ɸ 29 DNA polymerase buffer) and denatured at 85 °C for 5 min. After cooling the reaction mixture at ice for 5 min, 0.2 μl of ɸ29 DNA polymerase and pyrophosphatase (PPase) were added and incubated at 28 °C for 16–20 h. The reaction was stopped by heating at 65 °C for 5 min. Concatemers obtained in the RCA reaction were monomerized via restriction digestion using different restriction endonucleases. Restriction with *Kpn*1 and *Pst*1 gave required bands of virus and satellite, sized approximately 2.8 and 1.4 kb, respectively, which were subsequently selected for cloning in the pUC19 vector. While restriction with two other enzymes, *Xba*I and *Sac*I did not yield any bands of the required size. Cloned molecules were outsourced for Sanger sequencing in both orientations using the primer walking strategy (walking primer sequences has been shown in Additional file 3).

### Sequence assembly, analysis and identification of begomovirus and betasatellite

The Lasergene (V.8) package (DNASTAR, Madison, Wisconsin) was used to assemble and analyze the sequences. The sequences obtained were compared with similar publicly available sequences in the National Center for Biotechnology Information (NCBI) online database using BLASTn [18]. Coding and noncoding regions of virus/satellite clones were analyzed with the EditSeq module of Lasergene.

### Phylogenetic, recombination and SDT analysis

Sequences were aligned in MEGA6 software [33] by applying the MUSCLE algorithm [34] and the phylogenetic trees were constructed using maximum likelihood methods with 1000 bootstra*p* values. Related sequences were derived from GenBank and virus abbreviations were used as described previously [24]. As a requirement of begomovirus taxonomy [24], further confirmation of the viruses was performed using MUSCLE alignment in the sequence demarcation tool (SDT) program [35]. For recombination analysis, the sequences were converted into a single fasta file and opened in the RDP4 program [36]. From the X-over menu, the default RDP was used to detect the recombination among the sequences. The *p* values were used to validate the RDP results and are presented in Additional file 4.

### Construction of infectious molecules for infectivity analysis of virus and satellites

To check the infectivity, partial dimeric constructs were made for both PeLCV (Accession no: MF135486) and DiYVB (Accession no: MF135487) following a strategy described earlier [37], with few modifications. For construction of a PeLCV partial dimeric clone, the full-length clone (pMVL121, size 2.8 kb) was restriction-digested with *Kpn*I and *Bam*HI and converted into two fragments. The 2-kb fragment containing the origin of replication was cloned in pre-digested pUC19 giving rise to pUC19–2.0 kb. The full-length 2.8 kb molecule was ligated into pUC19–2.0 kb to generate the partial dimeric molecule pUC19–2.0 + 2.8. The partial dimeric molecule was restricted with *Sal*I and *Sac*I enzymes and shifted into pre-digested pCambia2300 vector.

A similar strategy was adopted to make a partial dimeric construct of DiYVB. The full-length clone was restriction-digested with *Pst*I and *Bam*HI into a 1.3 kb fragment containing origin of replication and ligated into pre-digested pGreen0029 at *Pst*I and *Bgl*II to generate the pGreen0029–1.3 kb vector [37]. The full-length betasatellite molecule (1.4 kb) was ligated into the pGreen0029–1.3 kb vector at the *Pst*I restriction site to generate the pGreen00291.3 + 1.4 vector. The partial dimeric molecule was digested with the *Hind*III and *Xba*I enzymes and shifted into pre-digested pCambia2300 vector.

*Nicotiana benthamiana* and *Petunia atkinsiana* plants were grown and maintained in a growth room at 28 °C and inoculated using a standard protocol for *Agro*infiltration [38]. Both of the partial dimeric molecules were mobilized into *A. tumefaciens* (GV3101) using the freeze and thaw method [39]. Thirty *N. benthamiana* plants were infiltrated with PeLCV alone and thirty *N. benthamiana* plants were co-inoculated with PeLCV and its cognate DiYVB by *Agrobacterium* infiltration with two replications, as described earlier [40]. Similarly, thirty *P. atkinsiana* plants were agro-inoculated with PeLCV alone and thirty *P. atkinsiana* plants were co-inoculated with PeLCV and DiYVB with two individual replications. As negative controls, five *N. benthamiana* and five *P. atkinsiana* plants were mock-inoculated with empty plasmid pCambia2300.

### Detection of virus and betasatellite DNAs in infiltrated plants

Total DNA was extracted from 10 mg leaf tissues from *N. benthamiana* and *P. atkinsiana* plants infiltrated with PeLCV, alone and in combination with DiYVB, after 14 dpi using the CTAB method [31]. For a negative control in southern blot hybridization, DNA also was extracted from mock-inoculated plants that showed no virus infection after 14 dpi. Three μg of total DNA were loaded on a 2% agarose gel and transferred to a nylon membrane, as specified by the manufacturer (Hybond N+; Amersham) and processed for Southern blot hybridization using a method described earlier [41]. The virus region encoding the *AC3* gene was amplified from full length PeLCV clone (Accession # MF135486) and used to design a Digoxigenin (DIG)-labeled probe. Similarly, the *βC1* region of DiYVB was amplified from DiYVB full length (Accession # MF135487) and used to make a probe. Specific primers to amplify the *AC3* gene of PeLCV and *βC1* of DiYVB are listed in Additional file 3. Hybridization signals were detected on the membrane after treatment with 5-bromo-4-chloro-3-indolyl phosphate (BCIP) and nitro blue tetrazolium chloride (NBT) (Thermo Scientific™, USA).

## Additional files

Additional file 1: Amplification and cloning of *Pedilanthus leaf curl virus* (PeLCV) and Digera yellow vein betasatellites (DiYVB). (a) Rolling circle amplification (RCA) (Lane 1) and DNA (Lane 2) from petunia leaves, Restriction of RCA product with *Pst*I (Lane 3) and *Kpn*I (lane 4). (b) Restriction confirmation of DiYVB clone in pUC19 with *Pst*I (Lane1 and 2). (c) Restriction confirmation of PeLCV clones with *Kpn*I and *BamH*I (Lane1, 2, 3). (d) Restriction of RCA products did not yield any required band of 2.8 or 1.4 kb by digestion with *Xba*I (lane 1 and 2) and *Sac*I (lane 3 and 4). M-1 kb marker (Thermo scientific). (DOCX 1347 kb)

Additional file 2: Host range and association of multiple betasatellites with PeLCV. (DOCX 45 kb)

Additional file 3: List of primers used in the study. (DOCX 17 kb)

Additional file 4: Average *p*-values of recombination events for PaLCuV and RaLCuV. (DOCX 16 kb)

Additional file 5: The DNA-A sequences used for recombination detection. (DOCX 62 kb)
