# Supplementary material for: Characterization, phylogeny and recombination analysis of Pedilanthus leaf curl virus-Petunia isolate and its associated betasatellite
Source: Virol J. 2018 Aug 31;15:134. doi: 10.1186/s12985-018-1047-y (PMC6117872; doi:10.1186/s12985-018-1047-y)
Supplement: Supplementary file 1 — Amplification and cloning of Pedilanthus leaf curl virus (PeLCV) and Digera yellow vein betasatellites (DiYVB). (a) Rolling circle amplification (RCA) (Lane 1) and DNA (Lane 2) from petunia leaves, Restriction of RCA product with PstI (Lane 3) and KpnI (lane 4). (b) Restriction confirmation of DiYVB clone in pUC19 with PstI (Lane1 and 2). (c) Restriction confirmation of PeLCV clones with KpnI and BamHI (Lane1, 2, 3). (d) Restriction of RCA products did not yield any required band of 2.8 or 1.4 kb by digestion with XbaI (lane 1 and 2) and SacI (lane 3 and 4). M-1 kb marker (Thermo scientific). (DOCX 1347 kb) [file 12985_2018_1047_MOESM1_ESM.docx]

*Virology Journal*: Research Article

Characterization, phylogeny and recombination analysis of Pedilanthus leaf curl virus-Petunia isolate and its associated betasatellite

Sara Shakir^1^, Muhammad Shah Nawaz-ul-Rehman^1^*, Muhammad Mubin^1^ and Zulfiqar Ali^2^

^1^Virology Lab, Center for Agricultural Biochemistry and Biotechnology, University of Agriculture, Faisalabad, 38000, Pakistan

^2^Muhammad Nawaz Sharif University of Agriculture, Multan, 59220, Pakistan

*****Corresponding author: [msnawazulrehman@uaf.edu.pk](mailto:msnawazulrehman@uaf.edu.pk)


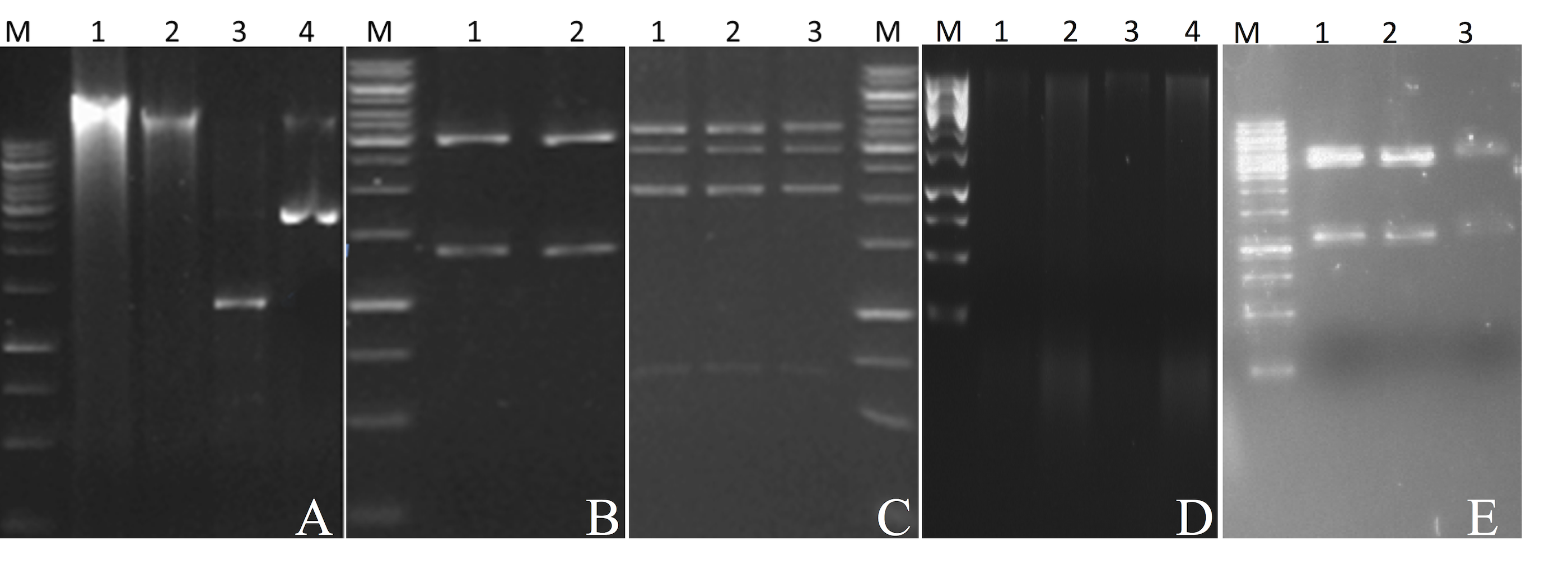


**Figure S1.** Amplification and cloning of *Pedilanthus leaf curl virus* (PeLCV) and Digera yellow vein betasatellites (DiYVB). **(a)** Rolling circle amplification (RCA) (Lane 1) and DNA (Lane 2) from petunia leaves, Restriction of RCA product with *Pst*I (Lane 3) and *Kpn*I (lane 4). **(b)** Restriction confirmation of DiYVB clone in pUC19 with *Pst*I (Lane1 and 2). **(c)** Restriction confirmation of PeLCV clones with *Kpn*I and *BamH*I (Lane1, 2, 3). **(d)** Restriction of RCA products did not yield any required band of 2.8 or 1.4 kb by digestion with *Xba*I (lane 1 and 2) and *Sac*I (lane 3 and 4). M-1kb marker (Thermo scientific).
