# Supplementary material for: Characterization, phylogeny and recombination analysis of Pedilanthus leaf curl virus-Petunia isolate and its associated betasatellite
Source: Virol J. 2018 Aug 31;15:134. doi: 10.1186/s12985-018-1047-y (PMC6117872; doi:10.1186/s12985-018-1047-y)
Supplement: Supplementary file 2 — Host range and association of multiple betasatellites with PeLCV. (DOCX 45 kb) [file 12985_2018_1047_MOESM2_ESM.docx]

*Virology Journal*: Research Article

Characterization, phylogeny and recombination analysis of Pedilanthus leaf curl virus-Petunia isolate and its associated betasatellite

Sara Shakir^1^, Muhammad Shah Nawaz-ul-Rehman^1^*, Muhammad Mubin^1^ and Zulfiqar Ali^2^

^1^Virology Lab, Center for Agricultural Biochemistry and Biotechnology, University of Agriculture, Faisalabad, 38000, Pakistan

^2^Muhammad Nawaz Sharif University of Agriculture, Multan, 59220, Pakistan

*****Corresponding author: [msnawazulrehman@uaf.edu.pk](mailto:msnawazulrehman@uaf.edu.pk)

**Additional file 2:** Host range and association of multiple betasatellites with PeLCV

| **No.** | **Name of the isolate** | **Associated betasatellite** | **Country of origin** | **Host** | **Reference/Accession** |
| --- | --- | --- | --- | --- | --- |
| 1 | PeLCV-[PK;Mul;Pedilanthus;04] | Tobacco leaf curl betasatellite | Pakistan | *Pedilanthus tithymaloides* | [[1](#_ENREF_1)] |
| 2 | PeLCV-[PK;Fai;Euphorbia;07] | - | Pakistan | *Euphorbia pulcherrima* | FM164938 |
| 3 | PeLCV-[PK;Chi;15] | Tobacco leaf curl betasatellite | Pakistan | *Raphanus sativus* | [[2](#_ENREF_2)] |
| 4 | PeLCV-[PK;Fai;Petunia;12] | Digera yellow vein betasatellite | Pakistan | *Petunia atkinsiana* | MF135486 (Current study) |
| 5 | PeLCV-[IN;Luc;Cestrum;11] | - | India | *Cestrum nocturnum* | [[3](#_ENREF_3)] |
| 6 | PeLCV-[IN;Luc;Crape;11] | - | India | *Tabernaemontana divaricata* | [[3](#_ENREF_3)] |
| 7 | PeLCV-[IN;ND;Carrot;16] | Digera yellow vein betasatellite | India | *Daucus carota* | [[4](#_ENREF_4)] |
| 8 | PeLCV-[IN;Bih;Tobacco;10] | Tomato leaf curl Patna betasatellite  Tobacco leaf chlorosis betasatellite | India | *Nicotiana tabacum* | [[5](#_ENREF_5)] |
| 9 | PeLCV-[PK;Mul;Spinach;07] | - | Pakistan | *Spinacia oleracea* | HF568781 |
| 10 | PeLCV-[PK;NS;Soybean;08] | Tobacco leaf curl Betasatellite | Pakistan | *Glycine max* | [[6](#_ENREF_6)] |
| 11 | PeLCV-[PK;RYK;Tomato;06] | - | Pakistan | *Solanum lycopersicum* | DQ116884 |
| 12 | PeLCV-[IN;Luc;Mentha;05] | - | India | *Mentha spicata* | [[7](#_ENREF_7)] |
| 13 | PeLCV-[PK;Yaz;Ses;13] | Tobacco leaf curl betasatellite | Pakistan | *Sesbania bispinosa* | [[8](#_ENREF_8)] |
| 14 | PeLCV-[PK;Isl;Cestrum;13] | - | Pakistan | *Cestrum nocturnum* | LM645010 |
| 15 | PeLCV-[PK;Fai;Chenopodium;05] | Digera yellow vein betasatellite  Cotton leaf curl Multan betasatellite | Pakistan | *Chenopodium album* | KY937947 |

**References**

1. Tahir M, Haider MS, Iqbal J, Briddon RW: **Association of a distinct begomovirus and a betasatellite with leaf curl symptoms in Pedilanthus tithymaloides.** *J Phytopathol* 2009, **157:**188-193.

2. Ismail H, Hassan I, Zubair M, Khan Z, Sarfaraz S, Jamil N, Mansoor S, Asad S, Amin I: **First report of *Pedilanthus leaf curl virus*, Tobacco leaf curl betasatellite and Guar leaf curl alphasatellite infecting radish (*Raphanus sativus*) in Pakistan.** *Plant Dis* 2017.

3. Srivastava A, Kumar S, Raj SK: **Association of *Pedilanthus leaf curl virus* with yellow mottling and leaf curl symptoms in two jasmine species grown in India.** *J Gen Plant Pathol* 2014, **80:**370-373.

4. Saritha RK, Shrawan S, Kalia P, Jain RK, Baranwal VK: **Association of *Pedilanthus leaf curl virus* and Satellites With Carrot (*Daucus carota*) in India.** *Plant Dis* 2016**:**PDIS-05-16-0672-PDN.

5. Kumar J, Singh SP, Kumar A, Khan JA, Tuli R: **Detection and characterization of a new betasatellite: variation in disease symptoms of *Tomato leaf curl Pakistan virus*-India due to associated betasatellite.** *Arch Virol* 2013, **158:**257-261.

6. Ilyas M, Qazi J, Mansoor S, Briddon RW: **Genetic diversity and phylogeography of begomoviruses infecting legumes in Pakistan.** *J Gen Virol* 2010, **91:**2091-2101.

7. Samad A, Mahesh K, Gupta., Shasany AK, Ajayakumar PV, Alam M: **Begomovirus related to *Tomato leaf curl Pakistan virus* newly reported in Mentha crops in India.** *Plant pathol* 2009, **58:**404.

8. Zaidi SS, Amin I, Iqbal Z, Akhtar KP, Scheffler BE, Mansoor S: **Sesbania bispinosa, a new host of a begomovirus-betasatellite complex in Pakistan.** *Can J Plant Pathol* 2016, **38:**107-111.
