# Supplementary material for: Characterization, phylogeny and recombination analysis of Pedilanthus leaf curl virus-Petunia isolate and its associated betasatellite
Source: Virol J. 2018 Aug 31;15:134. doi: 10.1186/s12985-018-1047-y (PMC6117872; doi:10.1186/s12985-018-1047-y)
Supplement: Supplementary file 3 — List of primers used in the study. (DOCX 17 kb) [file 12985_2018_1047_MOESM3_ESM.docx]

*Virology Journal*: Research Article

Characterization, phylogeny and recombination analysis of Pedilanthus leaf curl virus-Petunia isolate and its associated betasatellite

Sara Shakir^1,3^, Muhammad Shah Nawaz-ul-Rehman^1^*, Muhammad Mubin^1^ and Zulfiqar Ali^2^

^1^Virology Lab, Center for Agricultural Biochemistry and Biotechnology, University of Agriculture, Faisalabad, 38000, Pakistan

^2^Muhammad Nawaz Sharif University of Agriculture, Multan, 59220, Pakistan

^3^Present address: Boyce Thompson Institute, Ithaca, NY 14853, USA

*****Corresponding author: [msnawazulrehman@uaf.edu.pk](mailto:msnawazulrehman@uaf.edu.pk)

**Additional file 3.** List of primers used in the study

| **Virus/satellite name** | **Primer name** | **Primer sequence** |
| --- | --- | --- |
| PeLCV | PeLCV -*AC1*-F | 5ʹ-ATGGCTCCTCCCAAGCGATTTC-3ʹ |
| PeLCV | PeLCV -*AC1*-R | 5ʹ- TCAACACGACGACGCCTGGTCC-3ʹ |
| PeLCV | PeLCV-*AC3-*F | 5’-ATGGATTCACGCACAGGGGAAC-3’ |
| PeLCV | PeLCV-*AC3-*R | 5’- TTAATAAATATTGAATTTTATTG-3’ |
| PeLCV | PeLCV -Subseq1-F | 5ʹ-GAGGAACGATTCTCATTGGTC-3ʹ |
| PeLCV | PeLCV -Subseq1-R | 5ʹ-GGATTTTTACACCTCCTGTGGAGG-3ʹ |
| PeLCV | PeLCV -Subseq2-R | 5ʹ-CAGTGACCTGGCCCACATC-3ʹ |
| DiYVB | DiYVB-*βC1*-F | 5ʹ-ATGACGATCAAATACAATAACC-3ʹ |
| DiYVB | DiYVB-*βC1*-R | 5ʹ-TTATACGGATGAATGCGTAAACG-3ʹ |
|  | M13-F | 5ʹ-GTAAAACGACGGCCAG-3ʹ |
|  | M13-R | 5ʹ-CAGGAAACAGCTATGAC-3ʹ |
