# Supplementary material for: Characterization, phylogeny and recombination analysis of Pedilanthus leaf curl virus-Petunia isolate and its associated betasatellite
Source: Virol J. 2018 Aug 31;15:134. doi: 10.1186/s12985-018-1047-y (PMC6117872; doi:10.1186/s12985-018-1047-y)
Supplement: Supplementary file 4 — Average p-values of recombination events for PaLCuV and RaLCuV. (DOCX 16 kb) [file 12985_2018_1047_MOESM4_ESM.docx]

*Virology Journal*: Research Article

Characterization, phylogeny and recombination analysis of Pedilanthus leaf curl virus-Petunia isolate and its associated betasatellite

Sara Shakir^1^, Muhammad Shah Nawaz-ul-Rehman^1^*, Muhammad Mubin^1^ and Zulfiqar Ali^2^

^1^Virology Lab, Center for Agricultural Biochemistry and Biotechnology, University of Agriculture, Faisalabad, 38000, Pakistan

^2^Muhammad Nawaz Sharif University of Agriculture, Multan, 59220, Pakistan

*****Corresponding author: [msnawazulrehman@uaf.edu.pk](mailto:msnawazulrehman@uaf.edu.pk)

**Additional file 4**: Average P-values of recombination events for PaLCuV and RaLCuV. Recombinant regions (A and B) are shown on figure 2. RDP4 was used to identify the average P-values of possible parental regions. A cut off P-value 0.05 was used throughout the analysis.

| **Isolate name** | **Event** | **RDP method** | **Average P value** |
| --- | --- | --- | --- |
| PaLCuV-[PK:Mia:07].FM955602 | A | RDP | 2.01 x 10^-40^ |
|  |  | GENECONV | 5.281 x 10^-50^ |
|  |  | Max Chi | 3.54 x 10^-02^ |
|  |  | Chimera | 9.763 x 10^-26^ |
|  |  | SiScan | 1.545 x 10^-36^ |
|  |  | 3Seq | 4.920 x 10^-73^ |
| RaLCuV-[IN:Bih:10].HQ257375 | B | RDP | 1.778 x 10^-06^ |
|  |  | GENECONV | 4.151 x 10^-08^ |
|  |  | Max Chi | 1.052 x 10^-03^ |
|  |  | Chimera | 4.590 x 10^-05^ |
|  |  | SiScan | 5.864 x 10^-07^ |
|  |  | 3Seq | 1.305 x 10^-7^ |
