# Supplementary material for: Characterization, phylogeny and recombination analysis of Pedilanthus leaf curl virus-Petunia isolate and its associated betasatellite
Source: Virol J. 2018 Aug 31;15:134. doi: 10.1186/s12985-018-1047-y (PMC6117872; doi:10.1186/s12985-018-1047-y)
Supplement: Supplementary file 5 — The DNA-A sequences used for recombination detection. (DOCX 62 kb) [file 12985_2018_1047_MOESM5_ESM.docx]

*Virology Journal*: Research Article

Characterization, phylogeny and recombination analysis of Pedilanthus leaf curl virus-Petunia isolate and its associated betasatellite

Sara Shakir^1^, Muhammad Shah Nawaz-ul-Rehman^1^*, Muhammad Mubin^1^ and Zulfiqar Ali^2^

^1^Virology Lab, Center for Agricultural Biochemistry and Biotechnology, University of Agriculture, Faisalabad, 38000, Pakistan

^2^Muhammad Nawaz Sharif University of Agriculture, Multan, 59220, Pakistan

*****Corresponding author: [msnawazulrehman@uaf.edu.pk](mailto:msnawazulrehman@uaf.edu.pk)

**Additional file 5**: The fasta file of DNA-A sequences used to build the phylogeny and recombination in RDP software.

>RoLCuV-PKFai06GQ478342

ACCGGATGGCCGCGC---TTTTTTGTCCCCTCGTGGGTCCCAC--CAAGTGGTCCATG---------GACACATGGCCCAATCAAA-AGCACTCGTG-----------AAAGCTCAATTGATA-T-GTGGGCCCCAT---ATATAATTGCTTGCTGAGTAAGT-T-TG---TTGTAAAC-----------ATGTGGGACCCACT------ATTGAATGAGTTTCCAGAAACTGTTCATGGGTTTAGGTGTATGCTAGCAATT--------AAATACTTGCA-------GCTAGTAGAAAATACGTA-TTCCCCAGACAC---TCTGGGATACGAT--TTAATTAGGGATTTGATTTCAGTAATAAGGGCTAGGAATTATGTCGAAGCGACCAGCAGATATAATCATTTCCACGCCCGCCTCGAAGGTACGCCGCCGTCTCAACTTCGACAGCCCATATGCGAGCCGTGCTGCTGCCCCCATT---GTCCGCG-----------------TCACAAAGGCAAG---GGCATGGGCGAACAGGCCCATGAACAGAAAGCCCAGGATGTAC-AGGATGTACAGAAGCCCAGATGTTCC-GAGGGGATGT----GAAGGCCCATGCAAGGTCCAGTCATTTGAGTCCA-GACATGATATCCAGCACATT-GGTA-AAGTCATGTGTGTTAGTGATGTTACTCGTGGTATTGGGCTGACCCACAGGGTTGG---CAAGAGGTTCTGTGTTA-AGTCC----GTTTATGTTCTGGGCAAGATCTGGATGGATGAGAACATCAAGACTAAGAATCATACGAATAGTGTTATGTTTTTCC-TTGTTAGGGATCGTAGGCCTGTTGACAAG---CCTCAAGATTTTGGT-GAGGTGTTTAACATGTTTGATAATGAGCCCAGCACGGCGACTGTGAAGAATGTTCATCGTGATAGGTACCAGGT-ATTAAGGA---AGTGGCACGCA-----ACTGTGACAGGTGGCCTGTATGCATCGAAGGAGCAGGCTCTCGTGAAGAAG-TTTATTAGGGTTA-ATAATTATGT-TGTGTACAACCAG--------CAA-GAGGCTGGCAAGTATGAGAATCATACTGAGAATGCATTGATGTTGTATATGGCGTGTACCCACGCCTCTAACCCTGTGTATGCCACACTGAAGATACGGATCTATTTTTATGATTCAG-------TATCGAATTAATAAAATTTATCAGTATCGAATTAATAAATATTGAATTTTATTGAAGATGATTGTTTTACATATAC-----AATATGCTCTAATACATTCCATAATACATGATCAACTGCTCTAATTACATTGTTAATACTGATAACTCCTAGATTATCTAAATACTTAAGCAC-------TTGTGTCTTAAAG---------------ACCCTTAAGAAACGA-------------CCAGTCGGAG-GCTGTGAGGTC-----------ATCCAGATTC-GGAAGGCTAGGAAAC-ATTTGTGTATTCCC------AACGCTTT----------CCTCAGGTTGTGATTGAACTGTATCTGGACAGTGATGATGTCTTGGTTC--------ATGAGGAATGGCCTGTTGTGGTGCTCTGTTATCTTGAAATAGAGGGGATTTTGAACCTCCC--------AGATA------------AACACGCCATTCTGTGCTTGAGCT--GCAGTGATGGATTCCCCTGTGCG--TGAATCCATGGTTGTGGCAGTTGATGTGTACGTAG-------TATGAGCAGCCACACTCGAGGTC-AACCCTCTTACGCCGGATGGCTCTACGCTTGGCCAGCCTGTGTTGGACCTTGA----TGGGCACCTGAGTACAGTGGCTCTGTGAGGGTGATGAATGT---------------------------TGCATTGTGTATAGC------CCAAGACTTCAGTGCTGAGTTCTTTTCCTCATCGAGGAACTCTTTATAGCT-GGAATTGGGCCCAGGATTGCATAGGAAGATC-------------------------------------GTGGGAATGCCCCCTTTAA-TTTGAACTGGCTTCCCGTACTTGGTGTTACTTTGCCAGTCCCTC-----------------------TGGGCCCCCATGAATTCTTTAAAGTG--------CTTTAGGTAGTGGGGGTCTACGTCATCAATGACG-TTG----------------TACCATGCATC-ATTTGAATAGAT---CTTAGGGCTCAGATCTAAATGGCCACATAGGTAATTATGTGGACCCAGTGACCTAGCCCACATTGTTTTCCCCGT-------ACGACTATCACCCTCA-----------ATGACGATACTTTTAGGTCTCAATGG-CCGCGCAGCGGGACCCATCACATTATCAGAGGCCCATTCCTCTATGGCCTCTGGAACTTGATCGAAGGAAGAAGAAAG------------------------------AAAAGGGGAAACATAAACCTCCATTGGAGGTGCAAAAAT-CCTATCTAGATTAGC---ATTAAGATTATGAAATTGTAAGACATAATCCTTTGGTGCTAGTTCCCTAATGACT-CTAAG--AGCCTCTGACTTACTGCCTGTGTTAATTGCCTGGGCGTAAGCATGCATTGGCTGACTGTT----GTCCCCCTCGTGCAGATCTTCCATCGATCTGAAACTCTCCCCAATCGAGGGTGTCTCCGT-CTTCTCCAGATAGGACTTGACGTCCGAGCTTGA---TTTAGCT-----CCATGAATGTTAGGATGGAAAT--GTGCTGACCTGGTTGGCGATACCAGGTCGAAGAATCG-GTTATTTGTGCACTGGTACTTCCCCTCGAACTGGAT-GAGCACGTGAAGAT---GAGGTTCCCCATTTTCGTGAAGCTCTC-TGCAGATCC--------TGATGAATTTTTTGTTTACTGG--GGTATGTATGTTTTGTAATTGATAGAGGGCTTCTTCTTTAGTTAGAGA----GCATTTGGGATAAGTGAGGAAGTAATTCTTAGCATTTATGCGGAA------GGCACGTGAC--ATT----TTGG---CAATCG-GTGT---ACAC---ACTAA------TTCTC--TGTCAA---TTG---GTGTAAGGGGGTA------CAA----------TATATA-CTTGTA-CACCAAATGGCAATT-TGGTAA--------TTCA-GGAACTTTAATTT-----GAATTTTGAA----ATTCAAAATT--CCCAAAA--GC-GGCCAT--CCGTA-TAATATT

>RaLCV-INVar11HQ698591

-------ACCGGATGGCCGCGA---TTTATT------TTGGGGCCCCCAC--AAAGCACTAACTGACA----ATGACATGTGGTCCAATGAGA-ATCGTTCCTC-----------GTAGCCTAATTGTTT-T-GTGGTCCCCTC----TATAA--ACTTAGTGCGCAAGC-TGTG---TTTCACATTC-----ACC-ATGTGGGATCCGTT------ATTGAATGAGTTTCCCGAAACCGTTCACGGTTTTAGGTGTATGTTAGCAGTT--------AAATATCTGCA-------GTTAGTAGAAAATACGTA-TTCCCCAGATAC---TCTGGGATACGAT--TTAATTAGGGATTTGATTCCAGTAATAAGGGCCAGGAATTATGTCGAAGCGACCAGCAGATATAATCATTTCCACGCCCGCTTCGAAGGTACGTCGCCGTCTCAACTTCGACAGCCCATATGTGAGCCGTGTTGCTGCCCCCATT---GTCCGCG-----------------TCACCAAAGCAAA---AGCATGGGCGAACAGGCCCATGAACAGAAAGCCCAGGATGTAC-AGGATGTACAGAAGTCCAGATGTTCC-TAGAGGATGT----GAAGGCCCATGTAAGGTTCAGTCCTTTGAGTCCA-GACATGATATTCAGCATATA-GGTA-AAGTCATGTGTGTCAGTGATGTTACTCGTGGAACTGGGCTTACCCATAGAGTGGG---TAAAAGGTTTTGTGTTA-AGTCT----GTTTATGTTTTGGGCAAGATCTGGATGGATGAAAATATTAAAACTAAGAATCACACTAATAGTGTGATGTTCTTTC-TAGTTAGGGATCGTAGACCTGTAGATAAA---CCCCAAGATTTTGGA-GAGGTATTTAACATGTTTGATAATGAGCCCAGTACGGCTACTGTGAAGAATGTTCATCGTGATAGGTATCAAGT-TCTTCGGA---AATGGCATGCA-----ACTGTTACCGGTGGACAATATGCGTCAAAGGAACAAGCCCTCGTGAAGAAG-TTTGTTAGGGTTA-ATAATTATGT-TGTATATAACCAG--------CAA-GAAGCTGGCAAGTATGAGAATCATTCTGAGAATGCGTTAATGTTGTATATGGCATGTACTCATGCCTCTAACCCAGTGTATGCTACTTTGAAGATACGGATCTATTTCTATGATTCCG-------TAACAAA------------------------T---TAAATATTAAATTTTATTGAAT---ATTGTTCGACATATAC-----AATGTGTTGTAATACATTCCATAAAACATGATCAACTGATCTGATTACATTATTAATACTGATAACTCCTAAATTATCTAAATATTTTAAAAC-------TTGAGTCTTAAAG---------------ACCCTTAAGAAACGA-------------CCAGTCTGAG-GCTGTGAAGTC-----------ATCCAGATTC-GGTAGACTAGAAAAC-ACTTGTGCATCTCC------AACGCTTT----------CCTCAGGTTGTAGTTGAACTGTATTTGGACTTTGATTATGTCTTCTTTC--------ATTGTGAATGGACGGTTGTGGTGTTCTGTTATCTTGAAATACAGCCGATAATGAATCTCCC--------AGATA------------AACACGCCATTCTCTGCTTGAGCT--GCAGTGATGAGTTCCCCGGTGCG--TGAATCCATAATTGTGACAGGCTAGTGCTATGAAA-------TATGAACATCCACAAGGTAGATC-AACACGACGACGTC---TGTTCCCCTTCTTGGCATGCCTGTGCTGCACTTTGA----TTGGAACCTGAGTAGAGTGGGCCTTCGAGGGTGATGAAGGT---------------------------CGCATTCTTTAAAGC------CCAATTCT---GTGCAGCATTCTTCTCTTCATCCAAGAACACTTTATAACT-GGAATTGGGTCCTGGATTGCAGAGGAAGATA-------------------------------------GTGGGAATTCCGCCTTTAA-TTTGTTCTGGCTTTCCGTACTTTGTATTTGAAAGCCAGTCCCTT-----------------------TGGGCCCCCATGAATTCTTTAAAGTG--------CTTTAGGTAGTGGGGATCGACGTCATCAATGATA-TTG----------------TACCAGGCATC-ATTATTGTAGAC---CTTAGGGCTAAGGTCTAGATGTCCACACAAATAATTATGTGGTCCCAGTGACCTAGCCCACATTGTCTTCCCTGT-------CCTACTATCACCCTCA-----------ATCACTACACTTATTGGTCT-ATTGGCCCGCGCAGCGGCACTGACGACGTTCTCGGCAGCCCATTCCTCAAGTTCTTCCGGAACTTGATCAAAAGAAGAAGAAGA------------------------------AAAAGGAGAAACATAAACCTCCAGAGGAGGTGTATAAAT-CCTATCTAAATTAGC---ATTTAAATTATGGAATTGTAATACATAATCTTTTGGAGCTAACTCCTTAATGATT-CTAAG--AGCCTC---CTTACTGCCTGCGTTAAGCGCTGCGGCGTAAGCGT-CGTTGGCTGTCTGTT----GTCCCCCTCTAGCAGATCGTCCGTCGATCTGAAACTCTCCGGAGTCGAGGGTGTCTCCGTCCTTCTCCATATAGGACTTGACATCGGAGCTTGA---TTTAGCT-----CCCTGAATGTTCGGATGGAAAT--GTGCTGACCTGGTTGGGGATACGAGGTCGAAGAATCT-GTTATTTTTGCACTTGTATTTGCCTTCGAACTGGAT-GAGCACGCGAAGAT---GAGGTTCCCCATTTTCATGAAGCTCTC-TGCAGATTT--------TAATGTATTTTTTGTTTACTGG--GGTTTGTAGGTTTTGTAGTTGGGAAAGTGCTTCCTCTTTAGTAAGGGA----GCATTTGGGATAAGTAAGGAAATAATTTTTGGCATATATCTGAAATTGTTTGGGAGGAGCC--ATTGAC-TTGGT--CAATCG-GTAC---CCAGA--ACTAG------TCTTA--TGGCAA---TT----GGTGAACGGTACC------CTA----------TATATA--GTGGG-TACTGAATGGCAATATTTGTAA--------TTAT-GAAA--GAAATTC-----AAAATCCTCA----C------GCT--CCAATC---GC-GGCCAT--CCGTA-TAATATT

>RaLCV-INVar10JQ411026

-------ACCGGATGGCCGCGA---TTTTTT------TTGTGGCCCCCAC--AAAGCACTAACTGACA----ATGACATGTGGTCCAATGTGA-ATCGTTCCTC-----------GTAGCCTAATTGTTTGT-GTGGTCCCCTC----TATAA--ACTTAGTGCGCAAGT-TGTG---TTTTCCATTC-----AAT-ATGTGGGATCCGTT------ATTGAATGAGTTTCCCGAAACCGTTCACGGTTTTGGGTGTATGTTAGCAGTT--------AAATATCTGCA-------GTTAGTAGAAAATACGTA-TTCCCCAGATAC---TCTGGGATACGAT--TTAATTAGGGATTTGATTTCAGTAATAAGGGCCAGGAATTATGTCGAAGCGACCAGCAGATATAATCATTTCCACGCCCGCTTCGAAGGTACGTCG-CGTCTCAACTTCGACAGCCCATATGTGCGCCGTGCTGCTGCCCCCATT---GTCCGCG-----------------TCACCAAAGCAAA---AGCATGGGCGAACAGACCCATGGACAGAAAGCCCAGGATGTAC-AGGATGTACAGAAGTCCAGATGTTCCGTAGAGGATGT----GAAGGCCCATGTAAGGTTCAGTCCTT-GAGTCCA-GACATGATATTCAGCATATACGGTA-AAGTCATGTGTGTCAGTGATGCTACTCGTGGAACTGGGCTTACCCATAGAGTGGG---TAAGAGATTTTGTGTGA-AGTCT----GTGTATGTTTTGGGTAAGATATGGATGGATGAGAACATTAAGACCAAGAATCACACGAATAGTGTGATGTTTTTCT-TAGTTAGAGATCGTAGACCAGTTGATAAA---CCTCAAGATTTTGGA-GAGGTGTTTAACATGTTTGATAATGAGCCCAGTACGGCGACTGTGAAGAATGTTCATCGTGATAGGTATCAAGT-GCTGCGCA---AATGGTATGCA-----ACTGTCACCGGTGGACAATACGCTTCAAAGGAACAAGCTCTCGTGAAGAAG-TTTATTAGGGTTA-ATAATTATGT-TGTGTATAACCAG--------CAA-GAAGCTGGCAAGTATGAGAATCATTCTGAGAATGCTTTAATGTTGTATATGGCGCGTACTCACGCCTCTAACCCAGTGTATGCTACTTTGAAAATACGGATCTACTTCTATGATTCCG-------TGACAAA------------------------TTAATAAATATTGAATTTTATTGAAGATGATTGGTCTACATATAC------ACATGCTCTAATACATTCCATAATACATGAGCAACTGCTCCAA-TACATTATTAATACTGACAATTCCGAAGTTATGTAAATACTTAAGCAC-------TTGGGTCTTAAAG---------------ACCCGGAAGAAACGA-------------CCAATCGGAG-GCTGTGAGGTCGAGACAGACGAGTCCAGATTC-G------TAGAAAAC-ACTGGTGTATCCCC------AACGCTTT----------CCGCAGGTTGTAGTTGAACTGGACTTGTATGGTGAGTATGTCGTTGTTG--------GATAGAAATGGTCTCTCGTGGTGTTGGGATATCTTGAAATAGAGGGGATTTTTGACCGTCC--------AAATA------------TACACGCCACTCTCTGCCTGAGTT--GCAGTGATGAGTTCCCCTGTGCG--TGAATCCATGTGATGCGCAGGCAAGTCCAAAATAG-------TAAGAGCAACCGCAGGGAAGATCTAATGCGAA-----C---TGGTCT-CTTCTTGGCTATTCTGTGCTGGACTTTGA----TGGGTACCTGAGTACAACGGCTCGGTGAGGGTGAAGAATTC---------------------------TGCATTCTTTAATGC------CCAGTCTTTTAGTGCTGAATTCTTTTCCTCATCCAAGTACTCTTTATATGA-TGATGTCGGGCCTGGATTGCAGAGGAAGATA-------------------------------------GTGGGAATCCCACCTTTAATTTTGCATGGGCTTCCCGTACTTTGTGTT-GCTTGCCAGTCCCTT-----------------------TGGGCCCCCATGAACTCCTTAAAGTG--------CTTTAGATAGTGGGGATCGACGTCA-CAATGACGTTTG----------------TACCAGGCATC-ATTACTGTACAC---CTTTGGACTCAGGTCAAGGTGACCACATAAATAGTTATGTGGACCCAGAGACCTAGCCCACATTGTCTTCCCTGT-------CCTACTATCACCCTCA-----------ATCACTACACTTATTGGTCT-ATTGGCCCGCGCAGCGGCACTGACGACGTTCTCGGCAGCCCATTCCTCAAGTTCTTCCGGAACTTGATCAAAAGAAGAAGAAGA------------------------------AAAAGGAGAAACATAAACCTCCAGAGGAGGTGTATAAAT-CCTATCTAAATTAGC---ATTTAAATTATGGAATTGTAATACATAATCTTTTGGAGCTAACTCCTTAATGATT-CTAAG--AGCCTCTGACTTACTGCCTGCGTTAAGCGCTGCGGCGTAAGCGT-CGTTGGCTGTCTGTT----GTCCCCCTCTAGCAGATCGTCCGTCGATCTGAAACTCTCCCCAGTCGAGGGTGTCTCCGTCCTTCTCCATATAGGACTTGACATCGGAGCTTGA---TTTAGCT-----CCCTGAATGTTCGGATGGAAAT--GTGCTGACCTGGTTGGGGATACGAGGTCGAAGAATCT-GTTATTTTTGCACTTGTATTTGCCTTCGAACTGGAT-GAGCACGCGAAGAT---GAGGTTCCCCATTTTCATGAAGCTCTC-TGCAGATTT--------TAATGTATTTTTTGTTTACTGG--GGTTTGTAGGTTTTGTAGTTGGGAAAGTGCTTCCTCTTTAGTAAGGGA----GCATTTGGGATAAGTAAGGAAATAATTTTTGGCATATATCTGAAATTGTTTGGGAGGAGCC--ATTGAC-TTGGT--CAATCG-GTAC---CCAGA--ACTAG------TCTTA--TGGCAA---TT----GGTGAACGGTACC------CTA----------TATATA--GTGGG-TACTGAATGGCAATATTTGTAA--------TTAT-GAAA--GAAATTC-----AAAATCCTCA----C------GCT--CCAATC---GC-GGCCAT--CCGTA-TAATATT

>RaLCV-INVar10HQ682190

-------ACCGGATGGCCGCGA---TTTATT------ATGGGGCCCGCAC--AAAGCACTAACTGACA----ATGACATGTGGTCCAATGAGA-ATCGTTCCTC-----------GTAGCCTAATTGTTT-T-GTGGTCCCCTC----TATAA--ACTTAGTGCGCAAGC-TGTG---TTTCACATTC-----ACC-ATGTGGGATCCGTT------ATTGAATGAGTTTCCCGAAACCGTTCACGGTTTTAGGTGTATGTTAGCAGTT--------AAATATCTGCA-------GTTAGTAGAAAATACGTA-TTCCCCAGATAC---TCTGGGATACGAT--TTAATTAGGGATATGATTCCAGTAATAAGGGCCAGGAATTATGTCGAAGCGACCAGCAGATATAATCATTTCCACGCCCGCTTCGAAGGTACGTCGCCGTCTCAACTTCGACAGCCCATATGTGAGCCGTGTTGCTGCCCCCATT---GTCCGCG-----------------TCACCAAAGCATT---AGCATGGGCGAACTGCCCCATGTATAGAAAGCCAAGGATGTAC-AGGATGTACAGAAGCCCAGATGTCCC-TAGAGGATGT----GAAGGCCCATGTAAGGTCCAGTCGTT---GTCTA-GACATGACATTCAGCATATA-GGTA-AAGTTATGTGTATTAGTGATGTTACTCGTGGAACTGGGCTGACCCATCGAGTGGG---TAAAAGGTTTTGTGTTA-AGTCT----GTTTATGTTTTGGGCAAGATCTGGATG---GAAAATATTAAAACTAAGAATCACACTAATAGTGTGATGTTCTTTC-TAGTTAGGGATCGTAGACCTGTAGATATT---CCCCAAGATTTTGGA-GAGGTATTTAACATGTTTGATAATGAGCCCAGTACGGCTACTGTGAAGAATGTTCATCGTGATAGGTATCAAGT-TCTTCGGA---AATGGCATGCA-----ACTGTTACTGGTGGACAATATGCGTCAAAGGAACAAGCCCTCGTGAAGAAG-TTTGTTAGGGTTA-ATAATTATGT-TGTATATAACCAG--------CAA-GAAGCTGGCAAGTATGAGAATCATTCTGAGAATGCGTAAATGTTGTATATGGCATGTACTCATGCCTCTAACCCAGTGTATGCTACTTTGAAGATACGGATCTATTTCTATGATTCCG-------TAACAAA------------------------TTAATAAATATTAAATTTTATTGAATATGATTGTTCGACATATAC-----AATGTGTTGTAATACATTCCATAAAACATGATCAAC---TCTGATTACATTATTAATACTGATAACTCCTAAATTATCTAAATATTTTAAAAC-------TTGAGTCTTAAAG---------------ACGCTTAAGAAACGA-------------CCAGTCTGAG-GCTGTGAAGTC-----------ATCCAGATTC-GGTAGACTAGAAAAC-ACTTGTGCATCTCC------AACGCTTT----------CCTCAGGTTGTAGTTGAACTGTATTTGGACTTTGATTATGTCTTCTTTC--------ATTGTGAATGGACGGTTGTGGTGTTCTGTTATCTTGAAATACAGGCCATTTTGAATCTCCC--------AGATA------------AACACGCCATTCTCTGCTTGAGCT--GCAGTGATGAGTTCCCCGGTGCG--TGAATCCATAATTGTGACAGGCTAGTGCTATGAAA-------TATGAACATCCACAAGGTAGATC-AACACGACGACGTC---TGTTCCGCTTCTTGGCATGCCTGTGCTGCACTTTGA----TTGGAACCTGAGTAGAGTGGGCCTTCGAGGGTGATGAAGGT---------------------------CGCATTCTTTAAAGC------CCAATTCTTGAGTGCAGCATTCAACTCTTCATCCAAGAACACTTTATAACT-GGAATTGGGTCCTGGATTGCAGAGGAAGATA-------------------------------------GTGGGAATTCCGCCTTTAA-TTTGTTCTGGCTTTCCGTACTTTGTATTTGATAGCCAGTCCCTT-----------------------TGGGCGCCGATGAATTCTTTAAAGTG--------CTTTAGGTAGTGCGGATCGACGTCATCAATGATA-TTG----------------TACCAGGCATC-ATTATTGTAGAC---CTTAGGGCTAAGGTCTAGATGTCGACACAAATAATTATGTGGTCCCAGTGACCTAGCCCACATTGTCTTCCCTGT-------CCTACTATCACCCTCA-----------ATCACTACACTTATTGGTCT-ATTGGCCCGCGCAGCCCCACTGACGACGTTCTCGGCAGCCCATTCCTCAAGTTCTTCCGGAACTTGATCAAAAGAAGAAGAAGA------------------------------AAAAGGAGTTTCATATTCCTCCAGAGGAGGTGTATAAAT-CCTATCTAAATTAGC---ATTTAAATTATGGAATTGTAATACATAATCTTTTGGAGCTAACTCCTTAATGATT-CTAAG--AGCCTCTGACTTACTGCCTGCGTTAAGCGCTGCGGCGTAAGCGT-CGTTGGCTGTCTGTT----GTCCGCCTCTAGCAGATCGTCCGTCGATCTGAAACTCTCCCCAGTCGAGGGTGTCTCCGTCCTTCTCCATATAGGACTTGACATCGGAGCTTGA---TTTAGCT-----CCCTGAATGTTCGGATGGAAAT--GTGCTGACCTGGTTGGGGATACGAGGTCGAAGAATCT-GTTATTTTTGCACTTGTATTTGCCTTCGAACTGGAT-GAGCACGCGAAGAT---GAGGTTCCCCATTTTCATGAAGCTCTC-TGCAGATTT--------TAATGTATTAAATGTTTACTGG--GGTTTGTAGGTTTTGTAGTTGGGAAAGTGCTTCCTCTTTAGTAAGGGA----GCATTTGGGATAAGTAAGGAAATAATTTTTGGCATATATCTGAAATTGTTTGGGAGGAGCC--ATTGAC-TTGGT--CAATCG-GTAC---CCAGA--ACTAG------TCTTA--TGGCAA---TT----GGTGAACGGTAGG------GTA----------TATATA--GTGGG-TACTGAATGGCAATATTTGTAA--------TTAT-GAAA--GAAATTC-----AAAATCCTCA----C------GCT--CCAATC---GC-GGCCAT--CCGTA-TAATATT

>RaLCV-INVar03EF175733

-------ACCGGATGGCCGCGA---TTTTTT------TTGGGGCCCCCAC--AAAGCACTAACTGACA----ATGACATGTGGTCCAATGAGA-ATCGTTCCTC-----------GTAGCCTAATTGTTT-T-GTGGTCCCCTC----TATAA--ACTTAGTGCGCAAGC-TGTG---TTTCACATTC-----ACC-ATGTGGGATCCGTT------ATTGAATGAGTTTCCCGAAACCGTTCACGGTTTTAGGTGTATGTTAGCAGTT--------AAATATCTGCA-------GTTAGTAGAAAATACGTA-TTCCCCAGATAC---TCTGGGATACGAT--TTAATTAGGGATTTGATTCCAGTAATAAGGGCCAGGAATTATGTCGAAGCGACCAGCAGATATAATCATTTCCACGCCCGCTTCGAAGGTACGTCGCCGTCTCAACTTCGACAGCCCATATGTGAGCCGTGTTGCTGCCCCCATT---GTCCGCG-----------------TCACCAAAGCAAA---AGCATGGGCGAACAGGCCCATGAACAGAAAGCCCAGGATGTAC-AGGATGTACAGAAGTCCAGATGTTCC-TAGAGGATGT----GAAGGCCCATGTAAGGTTCAGTCCTTTGAGTCCA-GACATGATATTCAGCATATA-GGTA-AAGTCATGTGTGTCAGTGATGTTACTCGTGGAACTGGGCTTACCCATAGAGTGGG---TAAAAGGTTTTGTGTTA-AGTCT----GTTTATGTTTTGGGCAAGATCTGGATGGATGAAAATATTAAAACTAAGAATCACACTAATAGTGTGATGTTCTTTC-TAGTTAGGGATCGTAGACCTGTAGATAAA---CCCCAAGATTTTGGA-GAGGTATTTAACATGTTTGATAATGAGCCCAGTACGGCTACTGTGAAGAATGTTCATCGTGATAGGTATCAAGT-TCTTCGGA---AATGGCATGCA-----ACTGTTACCGGTGGACAATATGCGTCAAAGGAACAAGCCCTCGTGAAGAAG-TTTGTTAGGGTTA-ATAATTATGT-TGTATATAACCAG--------CAA-GAAGCTGGCAAGTATGAGAATCATTCTGAGAATGCGTTAATGTTGTATATGGCATGTACTCATGCCTCTAACCCAGTGTATGCTACTTTGAAGATACGGATCTATTTCTATGATTCCG-------TAACAAA------------------------TTAATAAATATTAAATTTTATTGAATATGATTGTTCGACATATAC-----AATGTGTTGTAATACATTCCATAAAACATGATCAACTGATCTGATTACATTATTAATACTGATAACTCCTAAATTATCTAAATATTTTAAAAC-------TTGAGTCTTAAAG---------------ACCCTTAAGAAACGA-------------CCAGTCTGAG-GCTGTGAAGTC-----------ATCCAGATTC-GGTAGACTAGAAAAC-ACTTGTGCATCTCC------AACGCTTT----------CCTCAGGTTGTAGTTGAACTGTATTTGGACTTTGATTATGTCTTCTTTC--------ATTGTGAATGGACGGTTGTGGTGTTCTGTTATCTTGAAATACAGGGGATTTTGAATCTCCC--------AGATA------------AACACGCCATTCTCTGCTTGAGCT--GCAGTGATGAGTTCCCCGGTGCG--TGAATCCATAATTGTGACAGGCTAGTGCTATGAAA-------TATGAACATCCACAAGGTAGATC-AACACGACGACGTC---TGTTCCCCTTCTTGGCATGCCTGTGCTGCACTTTGA----TTGGAACCTGAGTAGAGTGGGCCTTCGAGGGTGATGAAGGT---------------------------CGCATTCTTTAAAGC------CCAATTCTTGAGTGCAGCATTCTTCTCTTCATCCAAGAACACTTTATAACT-GGAATTGGGTCCTGGATTGCAGAGGAAGATA-------------------------------------GTGGGAATTCCGCCTTTAA-TTTGTTCTGGCTTTCCGTACTTTGTATTTGAAAGCCAGTCCCTT-----------------------TGGGCCCCCATGAATTCTTTAAAGTG--------CTTTAGGTAGTGGGGATCGACGTCATCAATGATA-TTG----------------TACCAGGCATC-ATTATTGTAGAC---CTTAGGGCTAAGGTCTAGATGTCCACACAAATAATTATGTGGTCCCAGTGACCTAGCCCACATTGTCTTCCCTGT-------CCTACTATCACCCTCA-----------ATCACTACACTTATTGGTCT-ATTGGCCCGCGCAGCGGCACTGACGACGTTCTCGGCAGCCCATTCCTCAAGTTCTTCCGGAACTTGATCAAAAGAAGAAGAAGA------------------------------AAAAGGAGAAACATAAACCTCCAGAGGAGGTGTATAAAT-CCTATCTAAATTAGC---ATTTAAATTATGGAATTGTAATACATAATCTTTTGGAGCTAACTCCTTAATGATT-CTAAG--AGCCTCTGACTTACTGCCTGCGTTAAGCGCTGCGGCGTAAGCGT-CGTTGGCTGTCTGTT----GTCCCCCTCTAGCAGATCGTCCGTCGATCTGAAACTCTCCCCAGTCGAGGGTGTCTCCGTCCTTCTCCATATAGGACTTGACATCGGAGCTTGA---TTTAGCT-----CCCTGAATGTTCGGATGGAAAT--GTGCTGACCTGGTTGGGGATACGAGGTCGAAGAATCT-GTTATTTTTGCACTTGTATTTGCCTTCGAACTGGAT-GAGCACGCGAAGAT---GAGGTTCCCCATTTTCATGAAGCTCTC-TGCAGATTT--------TAATGTATTTTTTGTTTACTGG--GGTTTGTAGGTTTTGTAGTTGGGAAAGTGCTTCCTCTTTAGTAAGGGA----GCATTTGGGATAAGTAAGGAAATAATTTTTGGCATATATCTGAAATTGTTTGGGAGGAGCC--ATTGAC-TTGGT--CAATCG-GTAC---CCAGA--ACTAG------TCTTA--TGGCAA---TT----GGTGAACGGTACC------CTA----------TATATA--GTGGG-TACTGAATGGCAATATTTGTAA--------TTAT-GAAA--GAAATTC-----AAAATCCTCA----C------GCT--CCAATC---GC-GGCCAT--CCGTA-TAATATT

>RaLCV-INRaj12KF218188

-------ACCGGATGGCCGCGA---TTTATT------TTGGGGCCCCCAC--AAAGCACTAACTGACA----ATGACATGTGGTCCAATGAGA-ATCGTTCCTC-----------GTAGCCTAATTGTTT-T-GTGGTCCCCTC----TATAA--ACTTAGTGCGCAAGC-TGTG---TTTCACATTC-----ACC-ATGTGGGATCCGTT------ATTGAATGAGTTTCCCGAAACCGTTCACGGTTTTAGGTGTATGTTAGCAGTT--------AAATATCTGCA-------GTTAGTAGAAAATACGTT-TTCCCCAGATAC---TCTGGGATACGAT--TTAATTAGGGATTTGATTCCAGTAATAAGGGCCAGGAATTATGTCGAAGCGACCAGCAGATATAATCATTTCCACGCCCGCTTCGAAGGTACGTCGCCGTCTCAACTTCGACAGCCCATATGTGAGCCGTGTTGCTGCCCCCATT---GTCCGCG-----------------TCACCAAAGCAAA---AGCATGGGCGAACAGGCCCATGAACAGAAAGCCCAGGATGTAC-AGGATGTACAGAAGTCCAGATGTTCC-TAGAGGATGT----GAAGGCCCATGTAAGGTTCAATCCTTTGAGTCCA-GACATGATATTCAGCATATA-GGTA-AAGTCATGTGTGTCAGTGATGTTACTCGTGGAACTGGGCTTACCCATAGAGTGGG---TAAAAGGTTTTGTGTTA-AGTCT----GTTTATGTTTTGGGCAAGATCTGGATGGATGAAAATATTAAAACTAAGAATCACACTAATAGTGTGATGTTCTTTC-TAGTTAGGGATCGTAGACCTGTAGAAAAA---CCCCAAGATTTTGGA-GAGGTATTTAACATGTTTGATAATGAGCCCAGTACGGCTACTGTGAAGAATGTTCATCGTGATAGGTATCAAGT-TCTTCGGA---AATGGCATGCA-----ACTGTTACCGGTGGACAATATGCGTCAAAGGAACAAGGCCTCGTGAAGAAG-TTTGTTAGGGTTA-ATAATTATGT-TGTATATAACCAG--------CAA-GAAGCTGGCAAGTATGAGAATCATTCTGAGAATGCGTTAATGTTGTATATGGCATGTACTCATGCCTCTAACCCAGTGTATGCTACTTTGAAGATACGGATCTATTTCTATGATTCCG-------TAACAAA------------------------T---TAAATATTAAATTTTATTGAAA---ATTGTTCGACATATAC-----AATGTGTTGTAATACATTCCATAAAACATGATCAACTGATCTGATTACATTATTAATACTGATAACTCCTAAATTATCTAAATATTTTAAAAC-------TTGAGTCTTAAAG---------------ACCCTTAAGAAACGA-------------CCAGTCTGAG-GCTGTGAAGTC-----------ATCCAGATTC-GGTAGACTAGAAAAA-ACTTGTGCATCTCC------AACGCTTT----------CCTCAGGTTGTAGTTGAACTGTATTTGGACTTTGATTATGTCTTCTTTC--------ATTGTGAATGGACGGTTGTGGTGTTTTGTTATCTTGAAATACAGCCGATAATGAATCTCCC--------AGATA------------AACACGCCATTCTCTGCTTGAGCT--GCAGTGATGAGTTGCCCGGTGCG--TGAATCCATAATTGTGACAGGCTAGTGCTATGAAA-------TATGAACATCCACAAGGTAGATC-AACACGACGACGTC---TGTTCCCCTTCTTGGCATGCCTGTGCTGCACTTTGA----TTGGAACCTGAGTAGAGTGGGCCTTCGAGGGTGATGAAGGT---------------------------CGCATTCTTTAAAGC------CCAATTCT---GTGCAGAATTCTTCTCTTCATCCAAGAACACTTTATAACT-GGAATTGGGTCCTGGATTGCAGAGGAAGATA-------------------------------------GTGGGAATTCCCCCTTTAA-TTTGTTCTGGTTTTCCGTACTTTGTATTTGAAAGCCAGTCCCTT-----------------------TGGGCCCCCATGAATTCTTTAAAGTG--------CTTTAGGTAGTGGGGATCGACGTCATCAATGATA-TTG----------------TACCAGGCATC-ATTATTGTAGAC---CTTAGGGCTAAGGTCTAGATGTCCACACAAATAATTATGTGGTCCCAGTGACCTAGCCCACATTGTCTTCCCTGT-------CCTACTATCACCCTCA-----------ATCACTACACTTATTGGTCT-ATTGGCCCGCGCAGCGGCACTGACGACGTTCTCGGCAGCCCATTCCTCAAGTTCTTCCGGAACTTGATCAAAAGAAGAAGAAGA------------------------------AAAAGGAGAAACATAAACCTCCAGAGGAGGGGTATAAAT-CCTATCTAAATTAGC---ATTTAAATTATGGAATTGTAATACATAATTTTTTGGAGCTAACTCCTTAATGATT-CTAAG--AGCCTC---CTTACTGCCTGCGTTAAGCGCTGCGGCGTAAGCGT-CGTTGGCTGTCTGTT----GTCCCCCTCTAGCAGATCGTCCGTCGATCTGAAACTCTCCGGAGTCGAGGGTGTCTCCGTCCTTCTCCATATAGGACTTGACATCGGAGCTTGA---TTTAGCT-----CCCTGAATGTTCGGATGGAAAT--GTGCTGACCTGGTTGGGGATACGAGGTCGAAGAATCT-GTTATTTTTGCACTTGTATTTGCCTTCGAACTGGAT-GAGCACGCGAAGAT---GAGGTTCCCCATTTTCATGAAGCTCTC-TGCAGATTT--------TAATGTATTTTTTGTTTACTGG--GGTTTGTAGGTTTTGTAGTTGGGAAAGTGCTTCCTCTTTAGTAAGGGA----GCATTTGGGATAAGTAAGGAAATAATTTTTGGCATATATCTGAAATTGTTTGGGAGGAGCC--ATTGAC-TTGGT--CAATCG-GTAC---CCAGA--ACTAG------TCTTA--TGGCAA---TT----GGTGAACGGTACC------CTA----------TATATA--GTGGG-TACTGAATGGCAATATTTGTAA--------TTAT-GAAA--GAAATTC-----AAAATCCTCA----C------GCT--CCAAT------------------TA-TAATATT

>RaLCV-InBih10HQ257375

-------ACCGGATGGCCGCGA---TTTTTT------TTGTGGCCCCCAC--AAAGCACTAACTGACA----ATGACATGTGGTCCAATGAGA-ATCGTTCCTC-----------GTAGCCTAATTGTTTGT-GTGGTCCCCTC----TATAA--ACTTAGTGCGCAAGT-TGTG---TTTTCCATTC-----AAT-ATGTGGGATCCGTT------ATTGAATGAGTTTCCCGAAACCGTTCACGGTTTTGGGTGTATGTTAGCAGTT--------AAATATCTGCA-------GTTAGTAGAAAATACGTA-TTCCCCAGATAC---TCTGGGATACGAT--TTAATTAGGGATTTGATTTCAGTAATAAGGGCCAGGAATTATGTCGAAGCGACCAGCAGATATAATCATTTCCACGCCCGCTTCGAAGGTACGTCG-CGTCTCAACTTCGACAGCCCATATGTGCGCCGTGCTGCTGCCCCCATT---GTCCGCG-----------------TCACCAAAGCAAA---AGCATGGGCGAACAGACCCATGGACAGAAAGCCCAGGATGTAC-AGGATGTACAGAAGTCCAGATGTTCCGTAGAGGATGT----GAAGGCCCATGTAAGGTTCAGTCCTT-GAGTCCA-GACATGATATTCAGCATATACGGTA-AAGTCATGTGTGTCAGTGATGCTACTCGTGGAACTGGGCTTACCCATAGAGTGGG---TAAGAGATTTTGTGTGA-AGTCT----GTGTATGTTTTGGGTAAGATATGGATGGATGAGAACATTAAGACCAAGAATCACACGAATAGTGTGATGTTTTTCT-TAGTTAGAGATCGTAGACCAGTTGATAAA---CCTCAAGATTTTGGA-GAGGTGTTTAACATGTTTGATAATGAGCCCAGTACGGCGACTGTGAAGAATGTTCATCGTGATAGGTATCAAGT-GCTGCGCA---AATGGTATGCA-----ACTGTCACCGGTGGACAATACGCTTCAAAGGAACAAGCTCTCGTGAAGAAG-TTTATTAGGGTTA-ATAATTATGT-TGTGTATAACCAG--------CAA-GAAGCTGGCAAGTATGAGAATCATTCTGAGAATGCTTTAATGTTGTATATGGCGCGTACTCACGCCTCTAACCCAGTGTATGCTACTTTGAAAATACGGATCTACTTCTATGATTCCG-------TGACAAA------------------------TTAATAAATATTGAATTTTATTGAAGATGATTGGTCTACATATAC------ACATGCTCTAATACATTCCATAATACATGAGCAACTGCTCCAA-TACATTATTAATACTGACAATTCCGAAGTTATGTAAATACTTAAGCAC-------TTGGGTCTTAAAG---------------ACCCGGAAGAAACGA-------------CCAATCGGAG-GCTGTGAGGTCGAGACAGACGAGTCCAGATTC-G------TAGAAAAC-ACTGGTGTATCCCC------AACGCTTT----------CCGCAGGTTGTAGTTGAACTGGACTTGTATGGTGAGTATGTCGTTGTTG--------GATAGAAATGGTCTCTCGTGGTGTTGGGATATCTTGAAATAGAGGGGATTTTTGACCGTCC--------AAATA------------TACACGCCACTCTCTGCCTGAGTT--GCAGTGATGAGTTCCCCTGTGCG--TGAATCCATGTGATGCGCAGGCAAGTCCAAAATAG-------TAAGAGCAACCGCAGGGAAGATCTAATGCGAAC--------TGGT-CTCTTCTTGGCTATTCTGTGCTGGACTTTGA----TGGGTACCTGAGTACAACGGCTCGGTGAGGGTGAAGAATTC---------------------------TGCATTCTTTAATGC------CCAGTCTTTTAGTGCTGAATTCTTTTCCTCATCCAAGTACTCTTTATATGA-TGATGTCGGGCCTGGATTGCAGAGGAAGATA-------------------------------------GTGGGAATCCCACCTTTAATTTTGCATGGGCTTCCCGTA----GTATTTGAAAGCCAGTCCCTT-----------------------TGGGCCCCCATGAATTCTTTAAAGTG--------CTTTAGGTAGTGGGGATCGACGTCATCAATGATA-TTG----------------TACCAGGCATC-ATTATTGTAGAC---CTTAGGGCTAAGGTCTAGATGTCCACACAAATAATTATGTGGTCCCAGTGACCTAGCCCACATTGTCTTCCCTGT-------CCTACTATCACCCTCA-----------ATCACTACACTTATTGGTCT-ATTGGCCCGCGCAGCGGCACTGACGACGTTCTCGGCAGCCCATTCCTCAAGTTCTTCCGGAACTTGATCAAAAGAAGAAGAAGA------------------------------AAAAGGAGAAACATAAACCTCCAGAGGAGGTGTATAAAT-CCTATCTAAATTAGC---ATTTAAATTATGGAATTGTAATACATAATCTTTTGGAGCTAACTCCTTAATGATT-CTAAG--AGCCTCTGACTTACTGCCTGCGTTAAGCGCTGCGGCGTAAGCGT-CGTTGGCTGTCTGTT----GTCCCCCTCTAGCAGATCGTCCGTCGATCTGAAACTCTCCCCAGTCGAGGGTGTCTCCGTCCTTCTCCATATAGGACTTGACATCGGAGCTTGA---TTTAGCT-----CCCTGAATGTTCGGATGGAAAT--GTGCTGACCTGGTTGGGGATACGAGGTCGAAGAATCT-GTTATTTTTGCACTTGTATTTGCCTTCGAACTGGAT-GAGCACGCGAAGAT---GAGGTTCCCCATTTTCATGAAGCTCTC-TGCAGATTT--------TAATGTATTTTTTGTTTACTGG--GGTTTGTAGGTTTTGTAGTTGGGAAAGTGCTTCCTCTTTAGTAAGGGA----GCATTTGGGATAAGTAAGGAAATAATTTTTGGCATATATCTGAAATTGTTTGGGAGGAGCC--ATTGAC-TTGGT--CAATCG-GTAC---CCAGA--ACTAG------TCTTA--TGGCAA---TT----GGTGAACGGTACC------CTA----------TATATA--GTGGG-TACTGAATGGCAATATTTGTAA--------TTAT-GAAA--GAAATTC-----AAAATCCTCA----C------GCT---CCAATC--GC-GGCCAT--CCGTA-TAATATT

>PedLCV-PKYazSes13LN713273

-------ACCGGATGGCCGCGA--TTTTTTT------TTGTGGCCCCCAC--AAAGCACTAACTGACA----ATGACCTGTTGACCAATGAGA-ATCGTTCCTC-----------ATAGCCTAATTGTTT-C-GTGGTCCCCCC----TATAA--ACGTAGTGCGCAAGT-TGTG---TTTTCCATTC-----GCT-ATGTGGGATCCGTT------ATTGAACGAGTTTCCCGAAACCGTTCACGGTTTTAGGTGTATGTTAGCAGTT--------AAATATCTGCA-------GTTAGTAGAAAATACGTA-TTCCCCAGATAC---TCTGGGATACGAT--TTAATTAGGGATTTGATTTCAGTAATAAGGGCTAGGAATTATGTCGAAGCGACCAGCAGATATAATCATTTCCACGCCCGTTTCGAAGGTACGCCGCCGTCTCAACTTCGACAGCCCCTATGGAGCTCGTGCAGTTGTCCCCATT---GCCCGCG-----------------TCACCAAAGCAAA---AGCATGGGCGAACAGGCCCATGAACAGAAAGCCCAGGATGTAC-AGGATGTACAGAAGTCCAGATGTTCC-TAGAGGATGT----GAAGGCCCATGTAAGGTCCAGTCCTTTGAGTCCA-GACATGATATCCAGCATATA-GGTA-AAGTCATGTGTGTCAGTGATGTTACTCGTGGAACTGGGCTGACCCATAGAGTGGG---TAAGAGATTTTGTGTCA-AGTCT----GTGTATGTGTTGGGCAAGATTTGGATGGATGAGAACATCAAGACCAAGAATCATACGAATAGTGTTATGTTTTTTT-TAGTTAGAGACCGTAGACCAGTTGACAAG---CCTCAGGATTTTGGA-GAGGTTTTTAACATGTTTGATAATGAGCCCAGTACGGCGACTGTGAAGAATGTGCATCGTGATAGGTACCAGGT-TCTGCGCA---AATGGTATGCA-----ACTGTCACCGGTGGACAATATGCGTCGAAGGAACAAGCTCTCGTGAAGAAG-TTTATTAGAGTTA-ATAATTATGT-TGTGTATAACCAG--------CAA-GAAGCTGGCAAGTATGAGAATCATTCTGAGAATGCGTTAATGTTGTATATGGCGTGTACTCACGCCTCTAATCCAGTGTATGCTACTTTGAAGATACGGATCTACTTCTATGATTCCG-------TGACAAA------------------------TTAATAAATATTGAATTTTATTGAAGATGATTGGTTTACATATAC-----AACATGCTCTAATACATTCCATAATACATGATCAACTGCTCTAACTACATTATTAATACTGATAACTCCTAGATTATCTAAATACTTAAGCAC-------TTGGGTCTTAAAG---------------ACCCTTAAGAAACGA-------------CCAGTCGGAG-GCTGTGAGGTC-----------ATCCAGATTC-GGAAGGCTAGGAAAC-ATTTGTGTATCCCC------AACGCTTT----------CCTCAGGTTGTGATTGAACTGTATCTGGACAGTGATGATGTCTTGGTTC--------ATGAGGAATGGTCTGTTGTGGTGCTCTGTTATCTTGAAATAGAGGGGATTTTGAATCTCCC--------AGATA------------AATACGCCATTCTGTGCTTGAGCT--GCAGTGATGAGTTCCCCTGTGCG--TGAATCCATGGTTGTGGCAGGCTAATGCTATGAAG-------TATGAACACCCACACGGGAGATC-AACACGACGACGCC---TGGTCCCCTTCTTGGCTAGCCTGTGCTGCACTTTGA----TTGGAACCTGAGTAGAGTGGGCCTTCGAGGGTGATGAAGGT---------------------------CGCATTCTTTAATGC------CCAATTTTTAAGTGCAGAATTCTTCTCCTCATCCAAAAACTCTTTATAGCT-TGAGTTGGGTCCTGGATTGCAGAGGAAGATA-------------------------------------GCGGGAATTCCGCCTTTAA-TTTGAACTGGCTTTCCGTACTTTGTATTTGATTGCCAGTCCCTT-----------------------TGGGCCCCCATGAATTCTTTAAAGTG--------CTTTAGGTAGTGGGGATCGACGTCATCAATGACG-TTG----------------TACCAGGCCTC-GTTGCTGTAGAC---CTTTGGACTAAGGTCTAAATGACCACACAGATAATTGTGTGGACCCAGTGACCTGGCCCACATCGTCTTCCCCGT-------TCTACTATCACCCTCT-----------AAGACAATACTTTTAGGTCTCAATGG-CCGCGCAGCGGCATCCACCACGTTCTCAGCAGCCCAGACTTCAAGTTCTTCCGGAACTTGATCAAAAGAAGAAGAAGA------------------------------AAAAGGAGAGACATAAACCTCCATAGGAGGTGTAAAAAT-CCTATCTAAATTAGC---ATTTAAATTATGAAATTGAAGTACATAATCTTTTGGTGCTAATTCTTTAATTACT-CTAAG--AGCCTCTGACTTACTGCCTGCGTTAAGCGCTGCGGCGTAAGCGT-CGTTGGCTGACTGTT----GTCCCCCTCTTGCAGATCTTCCATCGATCTGAAACTCTCCCCAGTCGAGGGTGTCTCCGTCCTTCTCCAGATAGGACTTGACGTCGGAGCTTGA---CTTAGCT-----CCCTGAATGTTCGGATGGAAAT--GTGCTGACCTGGTTGGGGATACGAGGTCGAAGAATCG-CTGATTTTGGCACTTGTATTTGCCCCCGAACTGGAT-GAGCACGTGAAGAT---GAGGTTCCCCATTTTCGTGGAGCTCTC-TGCAGATTT--------TAATGTATTTTTTGTTTACTGG--GGTTTGTAGGTTTTGGATTTGGGAAAGTGCTTCCTCTTTGGTGAGAGA----GCACTTGGGATAAGTGAGAAAGAAATTTTTGGCATATATTTGAAAACGCTTGGGAGGAGCC--ATTGAC-TTGGT--CAATCG-GTAC---TCAGC--ACTAG------TCCTA--TGGCAA---TC----GGTGATCAGTACT------CAA----------TATATA--GTGAG-TACCGAATGGCATTA-TCGTCA--------TTTG-GGAAA-GTAATTC-----AAAATCCTCA----C------GCT--CCAAAAA--GC-GGCCAT--CCGTA-TAATATT

>PedLCV-PkYazSes13LN713272

-------ACCGGATGGCCGCGA--TTTTTTT------TTGTGGCCCCCAC--AAAGCACTAACTGACA----ATGACCTGTTGACCAATGAGA-ATCGTTCCTC-----------ATAGCCTAATTGTTT-C-GTGGTCCCCCC----TATAA--ACTTAGTGCGCAAGT-TGTG---TTTTCCATTC-----ACT-ATGTGGGATCCGTT------ATTGAACGAGTTTCCCGAAACCGTTCACGGTTTTAGGTGTATGTTAGCAGTT--------AAATATCTGCA-------GTTAGTAGAAAATACGTA-TTCCCCAGATAC---TCTGGGATACGAT--TTAATTAGGGATTTGATTTCAGTAATAAGGGCTAGGAATTATGTCGAAGCGACCAGCAGATATAATCATTTCCACGCCCGTTTCGAAGGTACGCCGCCGTCTCAACTTCGACAGCCCCTATGGAGCTCGTGCAGTTGTCCCCATT---GCCCGCG-----------------TCACCAAAGCAAA---AGCATGGGCGAACAGGCCCATGAACAGAAAGCCCAGGATGTAC-AGGATGTACAGGAGTCCAGATGTTCC-TAGAGGATGT----GAAGGCCCATGTAAGGTCCAGTCCTTTGAGTCCA-GACATGATATCCAGCATATA-GGTA-AAGTCATGTGTGTCAGTGATGTTACTCGTGGAACTGGGCTGACCCATAGAGTGGG---TAAGAGATTTTGTGTCA-AGTCT----GTGTATGTGTTGGGCAAGATTTGGATGGATGAGAACATCAAGACCAAGAATCATACGAATAGTGTTATGTTTTTTT-TAGTTAGAGACCGTAGACCAGTCGACAAG---CCTCAGGATTTTGGA-GAGGTTTTTAACATGTTTGATAATGAGCCCAGTACGGCGACTGTGAAGAATGTGCATCGTGATAGGTACCAGGT-TCTGCGCA---AATGGTATGCA-----ACTGTCACCGGTGGACAATATGCGTCGAAGGAACAAGCTCTCGTGAAGAAG-TTTATTAGAGTTA-ATAATTATGT-TGTGTATAACCAG--------CAA-GAAGCTGGCAAGTATGAGAATCATTCTGAGAATGCGTTAATGTTGTATATGGCGTGTACTCACGCCTCTAATCCAGTGTATGCTACTTTGAAGATACGGATCTACTTCTATGATTCCG-------TGACAAA------------------------TTAATAAATATTGAATTTTATTGAAGATGATTGGTTTACATATAC-----AACATGCTCTAATACATTCCATAATACATGATCAACTGCTCTAACTACATTATTAATACTGATAACTCCTAGATTATCTAAATACTTAAGCAC-------TTGGGTCTTAAAG---------------ACCCTTAAGAAACGA-------------CCAGTCGGAG-GCTGTGAGGTC-----------ATCCAGATTC-GGAAGGCTAGGAAAC-ATTTGTGTATCCCC------AACGCTTT----------CCTCAGGTTGTGATTGAACTGTATCTGGACAGTGATGATGTCTTGGTTC--------ATGAGGAATGGTCTGTTGTGGTGCTCTGTTATCTTGAAATAGAGGGGATTTTGAATCTCCC--------AGATA------------AATACGCCATTCTGTGCTTGAGCT--GCAGTGATGAGTTCCCCTGTGCG--TGAATCCATGGTTGTGGCAGGCTAATGCTATGAAG-------TATGAACACCCACACGGGAGATC-AACACGACGACGCC---TGGTCCCCTTCTTGGCYAGCCTGTGCTGCACTTTGA----TTGGAACCTGAGTAGAGTGGGCCTTCGAGGGTGATGAAGGT---------------------------CGCATTCTTTAATGC------CCAATTTTTAAGTGCAGAATTCTTCTCCTCATCCAAAAACTCTTTATAGCT-TGAGTTGGGTCCTGGATTGCAGAGGAAGATA-------------------------------------GCGGGAATTCCGCCTTTAA-TTTGAACTGGCTTTCCGTACTTTGTATTTGATTGCCAGTCCCTT-----------------------TGGGCCCCCATGAATTCTTTAAAGTG--------CTTTAGGTAGTGGGGATCGACGTCATCAATGACG-TTG----------------TACCAGGCCTC-GTTGCTGTAGAC---CTTTGGACTAAGGTCTAAATGACCACACAGATAATTGTGTGGACCCAGTGACCTGGCCCACATCGTCTTCCCCGT-------TCTACTATCACCCTCT-----------AAGACAATACATTTAGGTCTCAATGG-CCGCGCAGCGGCATCCACCACGTTCTCAGCAGCCCAGACTTCAAGTTCTTCCGGAACTTGATCAAAAGAAGAAGAAGA------------------------------AAAAGGAGAGACATAAACCTCCATAGGAGGTGTAAAAAT-CCTATCTAAATTAGC---ATTTAAATTATGAAATTGAAGTACATAATCTTTTGGTGCTAATTCTTTAATTACT-CTAAG--AGCCTCTGACTTACTGCCTGCGTTAAGCGCTGCGGCGTAAGCGT-CGTTGGCTGACTGTT----GTCCCCCTCTTGCAGATCTTCCATCGATCTGAAACTCTCCCCAGTCGAGGGTGTCTCCGTCCTTCTCCAGATAGGACTTGACGTCGGAGCTTGA---CTTAGCT-----CCCTGAATGTTCGGATGGAAAT--GTGCTGACCTGGTTGGGGATACGAGGTCGAAGAATCG-CTGATTTTGGCACTTGTATTTGCCCTCGAACTGGAT-GAGCACGTGAAGAT---GAGGTTCCCCATTTTCGTGGAGCTCTC-TGCAGATTT--------TAATGTATTTTTTGTTTACTGG--GGTTTGTAGGTTTTGGATTTGGGAAAGTGCTTCCTCTTTGGTGAGAGA----GCACTTGGGATAAGTGTGAAAGAAATTTTTGGCATATATTTGAAAACGCTTGGGAGGAGCC--ATTGAC-TTGGT--CAATCG-GTAC---TCAGC--ACTAG------TCCTA--TGGCAA---TC----GGTGATCAGTACT------CAA----------TATATA--GTGAG-TACCGAATGGCATTA-TCGTCA--------TTTG-GGAAA-GTAATTC-----AAAATCCTCA----C------GCT--CCAAAAA--GC-GGCCAT--CCGTA-TAATATT

>PedLCV-PKYazSes13LN678638

-------ACCGGATGGCCGCGA--TTTTTTT------TTGTGGCCCCCAC--AAAGCACTAACTGACA----ATGACCTGTTGACCAATGAGA-ATCGTTCCTC-----------ATAGCCTAATTGTTT-C-GTGGTCCCCCC----TATAA--ACTTAGTGCGCATGT-TGTG---TTTTCCATTC-----ACT-ATGTGGGATCCGTT------ATTGAACGAGTTTCCCGAAACCGTTCACGGTTTTAGGTGTATGTTAGCAGTT--------AAATATCTGCA-------GTTAGTAGAAAATACGTA-TTCCCCAGATAC---TCTGGGATACGAT--TTAATTAGGGATTTGATTTCAGTGATAAGGGCTAGGAATTATGTCGAAGCGACCAGCAGATATAATCATTTCCACGCCCGTTTCGAAGGTACGCCGCCGTCTCAACTTCGACAGCCCCTATGGAGCTCGTGCAGTTGTCCCCATT---GCCCGCG-----------------TCACCAAAGCAAA---AGCATGGGCGAACAGGCCCATGAACAGAAAGCCCAGGATGTAC-AGGATGTACAGAAGTCCAGATGTTCC-TAGAGGATGT----GAAGGCCCATGTAAGGTCCAGTCCTTTGAGTCCA-GACATGATATCCAGCATATA-GGTA-AAGTCATGTGTGTCAGTGATGTTACTCGTGGAACTGGGCTGACCCATAGAGTGGG---TAAGAGATTTTGTGTCA-AGTCT----GTGTATGTGTTGGGCAAGATTTGGATGGATGAGAACATCAAGACCAAGAATCATACGAATAGTGTTATGTTTTTTT-TAGTTAGAGACCGTAGACCAGTCGACAAG---CCTCAGGATTTTGGA-GAGGTTTTTAACATGTTTGATAATGAGCCCAGTACGGCGACTGTGAAGAATGTGCATCGTGATAGGTACCAGGT-TCTGCGCA---AATGGCATGCA-----ACTGTCACCGGTGGACAATATGCGTCGAGGGAACAAGCTCTCGTGAAGAAG-TTTATTAGAGTTA-ATAATTATGT-TGTGTATAACCAG--------CAA-GAAGCTGGCAAGTATGAGAATCATTCTGAGAATGCGTTAATGTTGTATATGGCGTGTACTCACGCCTCTAATCCAGTGTATGCTACTTTGAAGATACGGATCTACTTCTATGATTCCG-------TGACAAA------------------------TTAATAAATATTGAATTTTATTGAAGATGATTGGTTTACATATAC-----AACATGCTCTAATACATTCCATAATACATGATCAACTGCTCTAACTACATTATTAATACTGATAACTCCTAGATTATCTAAATACTTAAGCAC-------TTGGGTCTTAAAG---------------ACCCTTAAGAAACGA-------------CCAGTCGGAG-GCTGTGAGGTC-----------ATCCAGATTC-GGAAGGCTAGGAAAC-ATTTGTGTATCCCC------AACGCTTT----------CCTCAGGTTGTGATTGAACTGTATCTGGACAGTGATGATGTCTTGGTTC--------ATGAGGAATGGTCTGTTGTGGTGCTCTGTTATCTTGAAATAGAGGGGATTTTGAATCTCCC--------AGATA------------AATACGCCATTCTGTGCTTGGGCT--GCAGTGATGAGTTCCCCTGTGCG--TGAATCCATGGTTGTGGCAGGCTAATGCTATGAAG-------TATGAACACCCACACGGGAGATC-AACACGACGACGCC---TGGTCCCCTTCTTGGCTAGCCTGTGCTGCACTTTGA----TTGGAACCTGAGTAGAGTGGGCCTTCGAGGGTGATGAAGGT---------------------------CGCATTCTTTAATGC------CCAATTTTTAAGTGCAGAATTCTTCTTCTCATCCAAAAACTCTTTATAGCT-TGAGTTGGGTCCTGGATTGCAGAGGAAGATA-------------------------------------GCGGGAATTCCGCCTTTAA-TTTGAACTGGCTTTCCGTACTTTGTATTTGATTGCCAGTCCCTT-----------------------TGGGCCCCCATGAATTCTTTAAAGTG--------CTTTAGGTAGTGGGGACCGACGTCATCAATGACG-TTG----------------TACCAGGCCTC-GTTGCTGTAGAC---CTTTGGACTAAGGTCTAAATGACCACACAGATAATTGTGTGGACCCAGTGACCTGGCCCACATCGTCTTCCCCGT-------TCTACTATCACCCTCT-----------AAGACAATACTTTTAGGTCTCAATGG-CCGCGCAGCGGCATCCACCACGTTCTCAGCAGCCCAGACTTCAAGTTCTTCCGGAACTTGATCAAAAGAAGAAGAAGA------------------------------AAAAGGAGAGACATAAACCTCCATAGGAGGTGTAAAAAT-CCTATCTAAATTAGC---ATTTAAATTATGAAATTGAAGTACATAATCTTTTGGTGCTAATTCTTTAATTACT-CTAAG--AGCCTCTGACTTACTGCCTGCGTTAAGCGCTGCGGCGTAAGCGT-CGTTGGCTGACTGTT----GTCCCCCTCTTGCAGATCTTCCATCGATCTGAAACTCTCCCCAGTCGAGGGTGTCTCCGTCCTTCTCCAGATAGGACTTGACGTCGGAGCTTGA---CTTAGCT-----CCCTGAATGTTCGGATGGAAAT--GTGCTGACCTGGTTGGGGATACGAGGTCGAAGAATCG-CTGATTTTGGCACTTGTATTTGCCCTCGAACTGGAT-GAGCACGTGAAGAT---GAGGTTCCCCATTTTCGTGGAGCTCTC-TGCAGATTT--------TAATGTATTTTTTGTTTACTGG--GGTTTGTAGGTTTTGGATTTGGGAAAGTGCTTCCTCTTTGGTGAGAGA----GCACTTGGGATAAGTGAGAAAGAAATTTTTGGCATATATTTGTAAACGCTTGGGAGGAGCC--ATTGAC-TTGGT--CAATCG-GTAC---TCAGC--ACTAG------TCCTA--TGGCAA---TC----GGTGATCAGTACT------CAA----------TATATA--GTGAG-TACCAAATGGCATTA-TCGTCA--------TTTG-GGAAA-GTAATTC-----AAAATCCTCA----C------GCT--CCAAAAA--GC-GGCCAT--CCGTA-TAATATT

>PedLCV-PKRYKTomato06DQ116884

-------ACCGGATGGCCGCGC----TTTTT------TTATGGCCCCCAC--AGAGCACTAACTGACA----ATGACATGTGGACCAATGAGA-ATCGTTCCTC-----------GTAGCCTAGTTATTT-C-ATGGTCCCCCC----TATAA--ACTTAGTGCGCAAGT-TGTG---TTTCACATTC-----ATT-ATGTGGGATCCGTT------GTTGAACGAGTTTCCCGAAACCGTTCACGGTTTTAGGTGTATGCTAGCAGTT--------AAATATTTGCA-------GTTAGTAGAAAATGCGTA-TTCCCCAGATAC---TTTGGGATACGAT--TTAATTAGGGATTTAATTTCAGTAATAAGGGCTAGGAATTATGTCGAAGCGACCAGCAGATATAATCATTTCCACGCCCGCCTCGAAGGTACGTCGCCGTCTCAATTTCGACAGCCCATATGCGAGCCGTGCTGCTGCCCCCATT---GTCCGCG-----------------TCACAAAGGCAAG---GGCATGGGCGAACAGGCCCATGAACCGAAAGCCCAGGATGTAC-AGGATGTACAGAAGCCCAGATGTTCC-TAGAGGATGT----GAAGGCCCGTGTAAGGTCCAGTCATTTGAGTCCA-GACATGATATCCAGCACATT-GGTA-AAGTCATGTGTGTTAGTGATGTTACTCGTGGTATTGGGCTGACCCACAGGGTAGG---CAAGAGATTCTGTGTCA-AGTCC----GTTTATGTTTTGGGCAAGATTTGGATGGATGAGAACATCAAGACCAAGAATCATACGAATAGTGTGATGTTTTTTC-TAGTTAGAGACCGTAGACCAGTTGATAAA---CCTCAGGATTTTGGA-GAGGTTTTTAATATGTTTGATAATGAGCCCAGCACGGCGACTGTGAAGAATGTTCATCGTGATAGATACCAGGT-ATTAAGGA---AGTGGTATGCA-----ACTGTCACTGGTGGACAATATGCATCGAAGGAGCAGGCTCTCGTGAAGAAG-TTTATTAGGGTTA-ATAATTATGT-TGTGTACAACCAG--------CAA-GAGGCTGGGAAGTATGAGAATCATACTGAGAATGCGTTAATGTTGTATATGGCGTGTACTCACGCCTCTAACCCTGTGTATGCTACATTGAAGATACGGATCTACTTCTATGATTCAG-------TATCGAA------------------------TTAATAAATATTGAATTTTATTGAAGATGATTGTTTTACATATAC-----AATATGCTCTAATACATTCCATAATACATGATCAACTGCTCTAATTACATTGTTAATACTGATAACTCCTAGATTATCTAAATACTTAAGCAC-------TTGTGTCTTAAAG---------------ACCCTTAAGAAACGA-------------CCAGTCGGAG-GCTGTGAGGTC-----------ATCCAGATTC-GGAAGGCTAGGAAAC-ATTTGTGTATTCCC------AACGCTTT----------CCTCAGGTTGTGATTGAACTGTATCTGGACAGTGATGATGTCTTGGTTC--------ATGAGGAATGGCCTGTTGTGGTGCTCTGTTATCTTGAAATAGAGGGGATTTTGAACCTCCC--------AGATA------------AACACGCCATTCTGTGCTTGAGCT--GCAGTGATGAGTTCCCCTGTGCG--TGAATCCATGGTCGTGGCAGGCTAAGGCTATGAAG-------TATGAACACCCACACGGGAGATC-AACACGACGACGCC---TGGTCCCTTTCTTGGCTAGCCTGTGCTGCACTTTGA----TTGGAACCTGAGTAGAGTGGGCCTTCGAGGGTGACGAAGAT---------------------------CGCATTCTTTAATGC------CCAATTTTTGAGTGCAGAATTCTTCTCCTCATCCAAAAACTCTTTATAGCT-TGAGTTGGGTCCTGGATTGCAGAGGAAGATA-------------------------------------GTGGGAATTCCGCCTTTAA-TTTGAACTGGCTTTCCGTACTTTGTATTTGATTGCCAGTCCCTT-----------------------TGGGCCCCCATGAATTCTTTAAAGTG--------CTTTAGGTAGTGGGGATCGACGTCATCAATGACG-TTG----------------TACCAGGCCTC-GTTGCTGTAAAC---CTTTGGACTAAGGTCTAGATGACCACACAGATAATTGTGTGGACCCAGTGACCTGGCCCACATTGTCTTCCCCGT-------ACGACTATCACCCTCT-----------ATGACAATACTTTTAGGTCTCAATGG-CCGCGCAGCGGCACCCACGACGTTCTCAGCAGCCCATTCTTCAAGTTCTTCCGGAACTTGATCAAAAGAAGAAGAAGA------------------------------AAAAGGAGAAACATAAACCTCCATGGGAGGTGTAAAAAT-CCTATCTAAATTAGC---ATTTAAATTATGAAATTGAAGTACATAATCTTTTGGTGCTAATTCTTTAATTACT-CTAAG--AGCTTCTGACTTACTGCCTGCGTTAAGCGCTGCGGCGTAAGCGT-CGTTGGCTGACTGTT----GTCCCCCTCTTGCAGATCTTCCATCGATCTGAAACTCTCCCCAGTCGAGGGTGTCTCCGTCCTTCTCCAGATAGGACTTGACGTCTGAGCTTGA---TTTAGCT-----CCCTGAATGTTCGGATGGAAAT--GTGCTGACCTGGTTGGGGATACCAGGTCGAAGAATCG-CTGATTTTGGCACTTGTATTTTCCCTCGAACTGGAT-GAGCACGTGAAGAT---GAGGTTCCCCATTTTCATGAAACTCTC-TGCAGATTT--------TAATGTATTTTTTGTTTACTGG--GGTTTGTAGGTTTTGGAATTGGGAAAGTGCTTCCTCTTTGGTGAGAGA----GCACTTGGGATAAGTGAGGAAATAATTTTTCGAATTAATAAGGAAACGCTTTGGGGGCATG---------TTGAC--CAAGTG-AGAGGACCCGATTGACCGC------TCTTG-----CAACTCTCCCCTGTATATCGGGTCT------CAA----------TATATA--GTGAG-ACCCAAATGGCATTA-TTGTAA--------TTTG-GATAA-CAGATTC-----AAAATTCTGA----C------GCT--CCAAAAA--GC-GGCCAT--CCGTA-TAATATT

>PedLCV-PKNSSoybean08AM948961

-------ACCGGATGGCCGCGATTTTTTTTT------TTGTGGTCCCCAC--AAAGCACTAACGGACA----ATGACATGTTGACCAATGAGA-ATTGTTCCTC-----------GTAGCCTAATTGTTT-C-GTGGTCCCCCC----TATAA--ACTTAGTGCGCAAGT-TGTG---TTTTCCATTC-----ACT-ATGTGGGATCCGTT------ATTGAACGAGTTTCCCGAAACCGTTCACGGTTTTAGGTGTATGTTAGCAGTT--------AAATATCTGCA-------GTTAGTAGAAAATACGTA-TTCCCCAGATAC---TCTGGGATACGAT--TTAATTAGGGATTTGATTTCAGTAATAAGGGCTAGGAATTATGTCGAAGCGACCAGCAGATATAATCATTTCCACGCCCGTTTCGAAGGTACGCCGCCGTCTCAACTTCGACAGCCCATACGTGAGCCGTGCTGCTGCCCCCATT---GTCCGCG-----------------TCACCAAAGCAAA---AGCATGGGCGAACAGGCCCATGAACAGAAAGCCCAGGATGTAC-AGGATGTACAGAAGTCCAGATGTTCC-TAGAGGATGT----GAAGGCCCATGTAAGGTCCAGTCCTTTGAGTCCA-GACATGATATCCAGCATATA-GGTA-AAGTCATGTGTGTCAGTGATGTTACTCGTGGAACTGGGCTGACCCATAGAGTGGG---TAAGAGATTTTGTGTCA-AGTCT----GTGTATGTGTTGGGCAAGATTTGGATGGATGAGAACATCAAGACCAAGAATCATACGAATAGTGTTATGTTTTTTT-TAGTTAGAGACCGTAGACCAGTTGACAAG---CCTCAGGATTTTGGA-GAGGTTTTTAACATGTTTGATAATGAGCCCAGTACGGCGACTGTGAAGAATGTGCATCGTGATAGGTACCAGGT-TCTGCGCA---AATGGTATGCA-----ACTGTCACCGGTGGACAATATGCGTCGAAGGAACAAGCTCTCGTGAAGAAG-TTTATTAGAGTTA-ATAATTATGT-TGTGTATAACCAG--------CAA-GAAGCTGGCAAGTATGAGAATCATTCTGAGAATGCGTTAATGTTGTATATGGCGTGTACTCACGCCTCTAATCCAGTGTATGCTACTTTGAAGATACGGATCTACTTCTATGATTCCG-------TGACAAA------------------------TTAATAAATATTGAATTTTATTGAAGATGATTGGTTTACATATAC-----AACATGCTCTAATACATTCCATAATACATGATCAACTGCTCTAACTACATTATTAATACTGATAACTCCTAGATTATCTAAATACTTAAGCAC-------TTGGGTCTTAAAG---------------ACCCTTAAGAAACGA-------------CCAGTCGGAG-GCTGTGAGGTC-----------ATCCAGATTC-GGAAGGCTAGGAAAC-ATTTGTGTATCCCC------AACGCTTT----------CCTCAGGTTGTGATTGAACTGTATCTGGACAGTGATGATGTCTTGGTTC--------ATGAGGAATGGTCTGTTGTGGTGCTCTGTTATCTTGAAATAGAGGGGATTTTGAATCTCCC--------AGATA------------AATACGCCATTCTGTGCTTGAGCT--GCAGTGATGAGTTCCCCTGTGCG--TGAATCCATGGTTGTGGCAGGCTAATGCTATGAAG-------TATGAACACCCACACGGGAGATC-AACACGACGACGCC---TGGTCCCCTTCTTGGCTAGCCTGTGCTGCACTTTGA----TTGGAACCTGAGTAGAGTGGGCCTTCGAGGGTGATGAAGGT---------------------------CGCATTCTTTAATGC------CCAATTTTTAAGTGCAGAATTCTTCTCCTCATCCAAAAACTCTTTATAGCT-TGAGTTGGGTCCTGGATTGCAGAGGAAGATA-------------------------------------GCGGGAATTCCGCCTTTAA-TTTGAACTGGCTTTCCGTACTTTGTATTTGATTGCCAGTCCCTT-----------------------TGGGCCCCCATGAATTCTTTAAAGTG--------CTTTAGGTAGTGGGGATCGACGTCATCAATGACG-TTG----------------TACCAGGCCTC-GTTGCTGTAGAC---CTTTGGACTAAGGTCTAAATGACCACACAGATAATTGTGTGGACCCAGTGACCTGGCCCACATCGTCTTCCCCGT-------TCTACTATCACCCTCT-----------AAGACAATACTTTTAGGTCTCAATGG-CCGCGCAGCGGCATCCACCACGTTCTCAGCAGCCCAGACTTCAAGTTCTTCCGGAACTTGATCAAAAGAAGAAGAAGA------------------------------AAAAGGAGAGACATAAACCTCCATAGGAGGTGTAAAAAT-CCTATCTAAATTAGC---ATTTAAATTATGAAATTGAAGTACATAATCTTTTGGTGCTAATTCTTTAATTACT-CTAAG--AGCCTCTGACTTACTGCCTGCGTTAAGCGCTGCGGCGTAAGCGT-CGTTGGCTGACTGTT----GTCCCCCTCTTGCAGATCTTCCATCGATCTGAAACTCTCCCCAGTCGAGGGTGTCTCCGTCCTTCTCCAGATAGGACTTGACGTCGGAGCTTGA---CTTAGCT-----CCCTGAATGTTCGGATGGAAAT--GTGCTGACCTGGTTGGGGATACAAGGTCGAAGAATCG-CTGATTTTGGCACTTGTATTTGCCCTCGAACTGGAT-GAGCACGTGAAGAT---GAGGTTCCCCATTTTCATGAAACTCTC-TGCAGATTT--------TAATGTATTTTTTGTTTACTGG--GGTTTGTAGGTTTTGGATTTGGGAAAGTGCTTCCTCTTTGGTGAGAGA----GCACTTGGGATAAGTGATAAAGAAATTTTTGGCATATATTTGAAAACGCTTGGGAGGAGCC--ATTGAC-TTGGT--CAATCG-GTAC---TCAGC--ATTAG------TCCTA--TGGCAA---TC----GGTGATTGGTACT------CAA----------TATATA--GTGAG-TACCGAATGGCATTA-TCGTAA--------TTTG-GGAAA-GTAATTC-----AAAATCCTCA----C------GCT--CCAAAAA--GC-GGCCAT--CCGTA-TAATATT

>PedLCV-PKMnSpinach07HF568781

-------ACCGGATGGCCGCGA---TTTTTT------TTGTGGCCCCCAC--AAAGCACTAACTGACA----ATGACATATTGACCAATCAGA-ATCGTTCCTC-----------ATAGCCTAATTGTTT-C-GTGGTCCCCCT----TATAA--ACTTAGTGCGCAAGT-TGTG---TTTTCCATTC-----ACT-ATGTGGGATCCGTT------ATTGAACGAGTTTCCCGAAACCGTTCACGGTTTTAGGTGTATGTTAGCAGTT--------AAATATCTGCA-------GTTAGTAGAAAATACGTA-TTCCCCAGATAC---TCTGGGATACGAT--TTAATTAGGGATTTGATTTCAGTAATAAGGGCTAGGAATTATGTCGAAGCGACCAGCAGATATAATCATTTCCACGCCCGCTTCGAAGGTACGCCGCCGTCTCAACTTCGACAGCCCATACGCGTGCCGTGCTGCTGCCCCCATT---GTCCGCG-----------------TCACCAAAGCAAA---AGCATGGGCGAACAGGCCCATGAACAGAAAGCCCAGGATGTAC-AGGATGTACAGAAGTCCAGATGTTCC-TAGAGGATGT----GAAGGCCCATGTAAGGTCCAGTCCTTTGAGTCCA-GACATGATATCCAGCATATA-GGTA-AAGTCATGTGTGTCAGTGATGTTACTCGTGGAACTGGGCTGACCCATAGAGTGGG---TAAGAGATTTTGTGTCA-AGTCT----GTGTATGTGTTGGGCAAGATTTGGATGGATGAGAACATCAAGACCAAGAATCATACGAATAGTGTTATGTTTTTTT-TAGTTAGAGACCGTAGACCAGTTGACAAG---CCTCAGGATTTTGGA-GAGGTTTTTAACATGTTTGATAATGAGCCCAGTACGGCGACTGTGAAGAATGTGCATCGTGATAGGTACCAGGT-TCTGCGCA---AATGGTATGCA-----ACTGTCACCGGTGGACAATATGCATCGAAGGAACAAGCTCTCGTGAAGAAG-TTTATTAGAGTTA-ATAATTATGT-TGTGTATAACCAG--------CAA-GAAGCTGGCAAGTATGAGAATCATTCTGAGAATGCGTTAATGTTGTATATGGCGTGTACTCACGCCTCTAATCCAGTGTATGCTACTTTGAAGATACGGATCTATTTCTATGATTCCG-------TGACAAA------------------------TTAATAAATATTGAATTTTATTGAAGATGATTGGTCTACATATAC-----AACATGCTCTAATACATTCCATAATACATGATCAACTGCTCTAAGTACATTATTAATACTGATAACTCCTAGATTATCTAAATACTTAAGCAC-------TTGGGTCTTAAAG---------------ACCCTTAAGAAACGA-------------CCAGTCGGAG-GCTGTGAGGTC-----------ATCCAGATTT-GGAAGGCTAGGAAAC-ATTTGTGTATCCCC------AACGCTTT----------CCTCAGGTTGTGATTGAACTGTATCTGGACAGTGATGATGTCTTGGTTC--------ATGAGGAATGGTCTGTTGTGGTGCTCTGTTATCTTGAAATAGAGGGGATTTTGAATCTCCC--------AGATA------------AATACGCCATTCTGTGCTTGAGCT--GCAGTGATGAGTTCCCCTGTGCG--TGAATCCATGGTTGTGGCAGGCTAATGCTATGAAG-------TATGAACACCCACACGGGAGATC-AACACGACGACGCC---TGGTCCCCTTCTTGGCTAGCCTGTGCTGCACTTTGA----TTGGAACCTGAGTAGAGTGGGCCTTCGAGGGTGATGAAGGT---------------------------CGCATTCTTTAATGC------CCAATTTTTAAGTGCAGAATTCTTCTCCTCATCCAAAAACTCTTTATAGCT-TGAGTTGGGTCCTGTATTGCAGAGGAAGATA-------------------------------------GTGGGAATTCCGCCTTTAA-TTTGAACTGGCTTTCCGTACTTTGTATTTGATTGCCAGTCCCTT-----------------------TGGGCCCCCATGAATTCTTTAAAGTG--------CTTTAGGTAGTGGGGATCGACGTCATCAATGACG-TTG----------------TACCAGGCCTC-GTTGCTGTAGAC---CTTTGGACTAAGGTCTAAATGACCACATAGATAATTGTGTGGACCCAGTGACCTGGCCCACATCGTCTTCCCCGT-------TCTACTATCACCCTCT-----------AAGACAATACTTTTAGGCCTCAAAGG-CCGCGCAGCGGCACTGACGACATTCTCGGCAGCCCACTCTTCAAGTTCTTCCGGAACTTGATCAAAAGAAGAAGAAGA------------------------------AAAAGGAGAAATATAAACCTCCACAGGAGGTGTAAAAAT-CCTATCTAAATTAGC---ATTTAAATTATGAAATTGAAGTACATAATCTTTTGGAGCTAACTCCTTAATTACT-CTAAG--AGCCTCTGACTTACTGCCCGCGTTAAGCGCTGCGGCGTAAGCGT-CGTTGGCTGACTGTT----GTCCCCCTCTTGCAGATCGTCCGTCGATCTGAAACTCTCCCCAGTCGAGGGTGTCTCCGTCCTTCTCCAGATAGGACTTGACATCTGAGCTTGA---CTTAGCT-----CCCTGTATGTTCGGATGGAAAT--GTGCTGACCTGGTTGGGGATACCAGGTCGAAGAATCT-GTTATTTTGGCACTTGTATTTTCCTTCGAACTGGAT-GAGCACGTGAAGAT---GAGGTTCCCCATTTTCGTGGAGCTCTC-TGCAGATTT--------TAATGTATTTTTTGTTTACTGG--GGTTTGTAGGTTTTGGATTTGGGAAAGTGCTTCCTCTTTAGTGAGAGA----GCATTTGGGATAAGTGAGAAAGAAATTTTTGGCATATATTTGAAAACGCTTGGGAGGAGCC--ATTGAC-TTGGT--CAATCG-GTAC---TCAGC--ACTAG------TCCTA--TGGCAA---TC----GGTGATCAGTACT------CAA----------TATATA--GTGAG-TACCGAATGGCATTA-TCGTAA--------TTTG-GGAAA-GTAATTC-----AAAATCCTCA----C------GCT--CCAAAAA--GC-GGCCAT--CCGTA-TAATATT

>PedLCV-PKIslCestrum13LM645010

-------ACCGGATGGCCGCGC--AATTTTTA-----TAGTGGTCCCGCC------CATTAAACTTTGTCGGGTAACATGTGGACCAACGAAATGGC-CTCCTC-----------GTGGTTTAATTGTTT-C-GTGGTCCCTCC----TATAA--ACTTAGTGCGCAAGT-TGTGCACTCTTACCA-----------ATGTGGGATCCATT------AGTAAACGAGTTTCCTGAAACCGTTCACGGTTTTAGATGTATGTTAGCAATT--------AAATATCTGCA-------GCTAGTAGAAAATACGTA-TTCCCCAGACAC---TCTGGGCTACGAT--TTAATTAGGGATTTGATTTCAGTTATTAGGGCTAGAAATTATGTCGAAGCGACCAGCAGATATAATCATTTCCACGCCCGCCTCGAAGGTACGTCGTCGTCTCAACTTCGACAGCCCATATGCCAGCCGTGCTGCTGCCCCCATT---GTCCGCG-----------------TCACAAAGGCAAG---AGCATGGGCGAACAGGCCCATGAACCGAAAGCCCAGGATGTAC-AGGATGTACAGAAGCCCAGATGTTCC-GAGGGGATGT----GAAGGCCCGTGTAAGGTCCAGTCATTTGAGTCCA-GACATGATATCCAGCACATT-GGTA-AAGTCATGTGTGTTAGTGATGTTACTCGTGGTATTGGGCTGACCCACAGGGTTGG---CAAGAGGTTCTGTGTGA-AGTCC----GTTTATGTTTTGGGCAAGATTTGGATGGATGAGAACATCAAGACTAAGAATCATACGAATA--GTTATGTTTTTTCTTGGTTAGGGATCGTAGGCCCGTTGACAAG---CCTCAAGATTTTGGGTGAGGTTTTTAACATGTTTGATAATGAGCCCAGCACGGCGACTGTGAAGAATGTTCATCGTGATAGATACCAGGTATTTAAGGA---AGTGGCAC--------GCTGTGACAGGCGGTCAGTATGCATCGAAGGAGCAGGCTCTCGTGAAGAAGTTTTTTTAGGGTTA-ATAATTATGT-TGTGTACAACCAG--------CAAGGAGGCTGGCAAGTATGAGAATCATACTGAGAATGCGTTGATGTTGTATATGGCGTGTACGCACGCCTCTAATCCTGTGTATGCTACATTGAAGATACGGATCTACTTCTATGATTCAG-------TATCGAA------------------------TTAATAAATATTGAATTTTATTGAAGATGATTGGTCTACATATAC-----AATATGCTCTAATACATTCCATAATACATGATCAACTGCTCTAACTACATTATTAATACTGACAACTCCTAGATTATCTAAATACTTAAGCAC-------TTGTGTCTTAAAT---------------ACCCTTAAGAAACGA-------------CCAGTCGGAG-GCTGTGAGGTC-----------ATCCAGATTC-GGAAGGCTAGGAAAC-ATTTGTGTATCCCC------AATGCTTT----------CCTCAGGTTGTGATTGAACTGTATCTGGACAGTGATGATGTCTTGGTTC--------ATGAGGAATGGCCTGTTGTGGTGCTCTGTTATCTTGAAATAGAGGGGATTTTGAATCTCCC--------AGATA------------AACACGCCATTCTGTGCTTGAGTT--GCAGTGATGAGTTCCCCTGTGCG--TGAATCCATGGTTGTGGCAGGCTAATGTTATGAAG-------TAAGAACACCCACAAGGGAGATC-AACTCTCCGACGTC---TGGTTTTCTTCTTGGCTAACCTGTGCTGCACTTTGA----TTGGAACCTGAGTAGAGTGGGCCTTCGAGGGTGACGAAGAT---------------------------CGCATTCTTTAAAGC------CCAATTTTTGAGTGCAGAATTCTTCTCCTCATCCAAGAACTCTTTATAGCT-GGAATTGGGCCCTGGATTGCAGAGGAAGATA-------------------------------------GTGGGAATGCCACCTTTAA-TTTGAACTGGCTTTCCGTATTTTGTATTTGATTGCCAGTCCCTT-----------------------TGGGCCCCCATGAACTCTTTAAAGTG--------CTTTAGGTAGTGGGGATCGACGTCATCAATGACG-TTG----------------TACCAGGCCTC-GTTACTGTATAC---CTTTGGACTAAGGTCTAAATGACCACACAAATAATTATGTGGACCCAGTGACCTAGCCCACATTGTCTTTCCCGT-------CCTACTATCACCCTCG-----------ATGACAATACTTTTAGGTCTCAATGG-CCGCGCAGCGGCACCCACCACATTTTCAGCAGCCCATTCCTCAAGTTCTTCCGGAACTTGATCAAAAGAAGAAGAAGA------------------------------GAAAGGAGAAACATAAACCTCCACAGGAGGTGTAAAAAT-CCTATCTAAATTAGC---ATTTAAATTATGAAAATGTAATACATAATCTTTTGGAGCTAACTCCTTAATGACT-CTGAG--AGCCTCTGACTTACTGCCTGTGTTAAGCGCTGCGGCGTAAGCGT-CGTTGGCTGTCTGTT----GTCCCCCTCTAGCAGATCGTCCGTCGATCTGAAACTCTCCCCATTCGAGGGTGTCTCCGTCCTTCTCCAGATAGGACTTGACGTCCGAGCTTGA---TTTAGCT-----CCCTGAATGTTCGGATGGAAAT--GTGCTGATCTGGTTGGGGATACCAGGTCGAAGAATCT-GTTATTTTGGCACTTGTACTTCCCTTCGAACTGGAT-GAGCACGTGAAGAT---GAGGTTCCCCATTTTCGTGGAGCTCTC-TGCAGATCT--------TTATGTATTTTTTGTTTACTGG--GGTTTGTAGGTTTTGTAATTGGGAAAGTGCCTCCTCTTTAGTAAGAGA----GCATTTGGGATAAGTGAGGAAATAATTTTTGGCATATATTTGAAAACGCTTAGGAGGAGCC--ATTGAC-TTGGT--CAATCG-GTAC---TCAGC--ACTAG------TCTTA--TGTCTA---TC----GGTGAATGGTACT------CAA----------TATATA--GTGAG-TAC-AAATGGCATTA-TTGTAA--------TTTG-GATAA-GAAATTC-----AAAATCCTCC-----------GCT--CCAAAAA--GC-GGCCAT--CCGTA-TAATATT

>PedLCV-PKFaiPet12

-------ACCGGATGGCCGCGA-TTTTTTTT------TTGTGGCCCCCAC--AAAGCACTAACTGACA----ATGACATGTTGACCAATGAGA-ATCGTTCCTC-----------GTAGCCTAATTGTTT-C-GTGGTCCCCCC----TATAA--ACTTAGTGCGCAAGT-TGTG---TTTTCCATTC-----ACT-ATGTGGGATCCGTT------ATTGAACGAGTTTCCCGAAACCGTTCACGGTTTTAGGTGTATGTTAGCAGTT--------AAATATCTGCA-------GTTAGTAGAAAATACGTA-TTCCCCAGATAC---TCTGGGATACGAT--TTAATTAGGGATTTGATTTCAGTAATAAGGGCTAGGAATTATGTCGAAGCGACCAGCAGATATAATCATTTCCACGCCCGTCTCGAAGGTACGCCGCCGTCTCAACTTCGACAGCCCATACGTGAGCCGTGCTGCTGCCCCCATT---GTCCGCG-----------------TCACCAAAGCAAA---AGCATGGGCGAACAGGCCCATGAACAGAAAGCCCAGGATGTAC-AGGATGTACAGAAGTCCAGATGTTCC-TAGAGGATGT----GAAGGCCCATGTAAGGTCCAGTCCTTTGAGTCCA-GACATGATATCCAGCATATA-GGTA-AAGTCATGTGTGTCAGTGATGTTACTCGTGGAACTGGGCTGACCCATAGAGTGGG---TAAGAGATTTTGTGTCA-AGTCT----GTGTATGTGTTGGGCAAGATTTGGATGGATGAGAACATCAAGACCAAGAATCATACGAATAGTGTTATGTTTTTTT-TAGTTAGAGACCGTAGACCAGTTGACAAG---CCTCAGGATTTTGGA-GAGGTTTTTAACATGTTTGATAATGAGCCCAGTACGGCGACTGTGAAGAATGTGCATCGTGATAGGTACCAGGT-TCTGCGCA---AATGGTATGCA-----ACTGTCACCGGTGGACAATATGCGTCGAAGGAACAAGCTCTCGTGAAGAAG-TTTATTAGAGTTA-ATAATTATGT-TGTGTATAACCAG--------CAA-GAAGCTGGCAAGTATGAGAATCATTCTGAGAATGCGTTAATGTTGTATATGGCGTGTACTCACGCCTCTAATCCAGTGTATGCTACTTTGAAGATACGGATCTACTTCTATGATTCCG-------TGACAAAT-----------------------TTAATAAATATTAAATTTTATTGAATATGATTGTTCTATATTTAC-----AACATGATGTAATACATTCCATAATACATGATCAACTGCTCTAATTACATTGTTAATACTGATAACTCCTAAATTATCTAAATACTTAAGTAC-------TTGGGTCTTAAAG---------------ACCCTTAAGAAACGA-------------CCAGTCGGAG-GCTGTGAGGTC-----------ATCCAGATTC-TGAAGGCTAGGAAAC-ATTTGTGTATCCCC------AACGCTTT----------CCTCAGGTTGTGATTGAACTGTATCTGGACGGTTATGATGTCTTGGTTC--------ATGGTGAATGGCCTGTTGTGGTGCTCTGTTATCTTGAAATAGAGGGGATTTTGAATCTCCC--------AGATA------------AACACGCCATTCTCTGCTTGAGCT--GCAGTGATGAGTTCCCCGGTGCG--TGAATCCATGACTGTGACAGGCTAGTGCTATGAAA-------TAAGAACATCCACAAGGGAGATC-AACACGTCGACGCC---TGGTCCCCTTCTTGGCTAGCCTGTGCTGCACTTTGA----TTGGAACCTGGGTAGAGTGGGCCTTCGAGGGTGACGAAGAT---------------------------CGCATTCTTTAATGC------CCAATTTTTAAGTGCAGAATTCTTCTCCTCATCCAAAAACTCTTTATAGCT-TGAGTTGGGTCCTGGATTGCAGAGGAAGATA-------------------------------------GCGGGAATTCCGCCTTTAA-TTTGAACTGGCTTTCCGTACTTTGTATTTGATTGCCAGTCCCTT-----------------------TGGGCCCCCATGGGATTCTTTAAGTG--------CTTTAGGTAGTGGGGATCGACGTCATCAATGACG-TTG----------------TACCAGGCCTC-GTTGCTGTAGAC---CTTTGGACTAAGGTCTAAATGACCACACAGATAATTGTGTGGACCCAGTGACCTGGCCCACATCGTCTTCCCTGT-------TCTACTATCACCCTCT-----------AAGACAATACTTTTAGGCCTCAAAGG-CCGCGCAGCGGCACTGACGACATTCTCGGCAGCCCACTCTTCAAGTTCTTCCGGAACTTGATCAAAAGAAGAAGAAGA------------------------------AAAAGGAGAAATATAAACCTCCACAGGAGGTGTAAAAAT-CCTATCTAAATTAGC---ATTTAAATTATGAAATTGAAGTACATAATCTTTTGGAGCTAACTCCTTAATTACT-CTAAG--AGCCTCTGACTTACTGCCTGCGTTAAGCGCTGCGGCGTAAGCGT-CGTTGGCTGACTGTT----GTCCCCCTCTTGCAGATCGTCCGTCGATCTGAAACTCTCCCCAGTCGAGGGTGTCTCCGTCCTTCTCCAGATAGGACTTGACATCTGAGCTTGA---CTTAGCT-----CCCTGTATGTTCGGATGGAAAT--GTGCTGACCTGGTTGGGGATACCAGGTCGAAGAATCT-GTTATTTTGGCACTTGTATTTTCCTTCGAACTGGAT-GAGCACGTGAAGAT---GAGGTTCCCCATTTTCGTGGAGCTCTC-TGCAGATTT--------TAATGTATTTTTTGTTTACTGG--GGTTTGTAGGTTTTGGATTTGGGAAAGTGCTTCCTCTTTAGTGAGAGA----GCATTTGGGATAAGTGAGAAAGAAATTTTTGGCATATATTTGAAATCGCTTGGGAGGAGCC--ATTGAC-TTGGT--CAATCG-GTAC---TCAGC--ACTAG------TCCTA--TGGCAA---TC----GGTGATCAGTACT------CAA----------TATATA--GTGAG-TACCGAATGGCATTA-TCGTAA--------TTTG-GGAAA-GTAATTC-----AAAATCCTCA----C------GCT--CCAAAAA--GC-GGCCAT--CCGTA-TAATATT

>PedLCV-PKFaiEuphor07FM164938

-------ACCGGATGGCCGCGC----TTTTT------TTATGGCCCCCAC--AGAGCACTAACTGACA----ATGACATGTGGACCAATGAGA-ATCGTTCCTC-----------GTAGCCTAATTATTT-C-ATGGTCCCCCC----TATAA--ACTTAGTGCGCAAGT-TGTG---TTTCACATTC-----ATT-ATGTGGGATCCGTT------GTTGAACGAGTTTCCCGAAACCGTGCACGGTTTTAGGTGTATGCTAGCAGTT--------AAATATTTGCA-------GTTAGTAGAAAATACGTA-TTCTCCAGATAC---TTTGGGATACGAT--TTAATTAGGGATTTAATTTCAGTAATAAGGGCTAGGAATTATGTCGAAGCGACCAGCAGATATAATCATTTCCACGCCCGCCTCGAAGGTACGTCGCCGTCTCAATTTCGACAGCCCATATGCGAGCCGTGCTGCTGCCCCCATT---GTCCGCG-----------------TCACAAAGGCAAG---GGCATGGGCGAACAGGCCCATGAACAGAAAGCCCAGGATGTAC-AGGATGTACAGAAGTCCAGATGTTCC-TAGAGGATGT----GAAGGCCCATGTAAGGTCCAGTCATTTGAGTCCA-GACATGGTATCCAGCACATT-GGTA-AAGTCATGTGTGTTAGTGATGTTACTCGTGGTATTGGGCTTACCCACAGGGTAGG---CAAGAGATTTTGTGTTA-AGTCC----GTTTATGTTTTAGGCAAGATTTGGATGGATGAGAACATTAAGACCAAGAATCATACGAATAGTGTGATGTTTTTTC-TAGTTAGGGATCGAAGGCCCGTTGACAAG---CCTCAGGATTTTGGA-GAGGTTTTTAATATGTTTGATAATGAGCCCAGCACGGCGACTGTGAAGAATGTTCATCGTGATAGATACCAGGT-ATTAAGGA---AGTGGTACGCA-----ACTGTGACAGGCGGTCAATATGCATCGAAGGAGCAAGCTCTCGTGAAGAAG-TTTATTAGGGTTA-ATAATTATGT-AGTGTACAACCAG--------CAA-GAGGCTGGGAAGTATGAGAATCATACTGAGAATGCGTTGATGTTGTATATGGCGTGTACGCACGCCTCTAACCCTGTGTATGCTACATTGAAGATACGGATCTACTTCTATGATTCAG-------TATCGAA------------------------TTAATAAATATTGAATTTTATTGAAGATGATTGTTCTATATTTAC-----AATATGCTCTAATACATTCCATAATACATGATCAACTGCCCTAACTACATTATTTATACTGATAACTCCTAGATTATCTAAATACTTAAGCAC-------TTGGGTCTTAAAG---------------ACCCTTAAGAAACGA-------------CCAGTCGGAG-GCTGTGAGGTC-----------ATCCAGATTC-GGAAGGCTAGGAAAC-ATTTGTGTATCCCC------AAAGCTTT----------CCTCAGGTTGTGATTGAACTGTATCTGGACGGTGATGATGTCTTGGTTC--------ATGGTGAATGGCCTATTGTGTTGCTCTGTTATCTTGAAATAGAGGGGATTTTGAATCTCCC--------AGATA------------AACACGCCATTCTGTGCTTGAGCT--GCAGTGATGAGTTCCCCTGTGCG--TGAATCCATGGTCGTGGCAGGCTAATGCTATGAAG-------TATGAACACCCACACGGGAGATC-AACACGACGACGCC---TGGTCCCCTTCTTGGCTAGCCTGTGCTGCACTTTGA----TTGGAACCTGAGTAGAGTGGGCCTTCGAGGGTGATGAATGT---------------------------CGCATTCTTTAATGC------CCAATTTTTAAGTGCAGAATTCTTCTCCTCATTCAAAAACTCTTTATAGCT-TGAGTTGGGTCCTGGATTGCAGAGGAAGATA-------------------------------------GTGGGAATTCCGCCTTTAA-TTTGAACTGGCTTTCCGTACTTGGTGTTGCTTTGCCAGTCCCTT-----------------------TGGGCCCCCATGAATTCTTTAAAGTG--------CTTTAGGTAGTGGGGGTCGACGTCATCAATGACG-TTG----------------TACCAGGCCTG-GTTGCTGTAGAC---CTTTGGACTAAGGTCTAAATGACCACACAGATAATTATGTGGTCCAAGTGATCTGGCCCACATCGTCTTCCCCGT-------TCTACTATCACCCTCT-----------ATGACAATACTTTTAGGTCTCAATGG-CCGCGCAGCGGCATCCACCACATTCTCAGCAGCCCAGACTTCAAGTTCTTCCGGAACTTGATCAAAAGAAGAAGAAGA------------------------------AAAAGGAGAGACATAAACCTCCACAGGAGGTGTAAAAAT-CCTATCTAAATTAGT---ATTTAAATTATGAAATTGAAGTACATAATCTTTTGGTGCTAATTCTTTAATTACT-CTAAG--AGCCTCTGACTTACTGCCTGCGTTAAGCGCTGCGGCGTAAGCGT-CGTTGGCTGACTGTT----GTCCCCCTCTTGCAGATCTTCCATCGATCTGAAACTCTCCCCAGTCGAGGGTGTCTCCGTCCTTTTCCAGATAGGACTTGACGTCGGAGCTTGA---CTTAGCT-----CCCTGAATGTTCGGATGGAAAT--GTGCTGACCTGGTTGGGGATACCAGGTCGAAGAATCT-GTTATTTTGGCACTTGTATTTCCCCTCGAACTGGAT-GAGCACGTGAAGAT---GAGGTTCCCCATTTTCATGAAACTCTC-TGCAGATTT--------TAATGTATTTTTTGTTTACTGG--GGTTTGTAGGTTTTGGAATTGGGAAAGTGCTTCCTCTTTGGTGAGAGA----GCACTTGGGATAAGTGAGGAAATAATTTTTCGAATTAATAAGGAAACGCTTTGGAGGCATG---------TTGAC--CAAGTG-AGAGGACCCGATTGACCGC------TCTTG-----CAACTCTCCCCTGTATATCGGGTCT------CAA----------TATATA--GTGAG-ACCCAAATGGCATTA-TTGTAA--------TTTG-GATAA-CAGATTC-----AAAATTCTAA----C------GCT--CCAAAAA--GC-GGCCAT--CCGTA-TAATATT

>PedLCV-PKFaiChenopodium05KY937947

-------ACCGGATGGCCGCGC----TTTTT------TTATGGCCCCCAC--AGAGCACTAACTGACA----ATGACATGTGGACCAATGAGA-ATGGTTCCTC-----------ATAGCCTAATTATTT-C-ATGGTCCCCTC----TATAA--ACTTAGTGCGCAAGT-TGTG---TTTCACATTC-----ACT-ATGTGGGATCCGTT------ATTGAACGAGTTTCCCGAAACCGTTCACGGTTTTAGGTGTATGTTAGCAGTT--------AAATATTTGCA-------GTTAGTAGAAAGTACGTA-TTCCCCAGATAC---TCTGGGATTCGAT--TTAATTCGGGATTTAATTTCAGTGATAAGGGCTAGGAATTATGTCGAAGCGACCAGCAGATATAATCATTTCCACGCCCGCCTCGAANGTACGCCGCCGTCTGAACTTCGGCAGCCCATACGCGAGCCGTGCTGCTGCCCCCATT---GTCCGCG-----------------TCACAAAGGCAAG---GGCATGGGCGAACAGGCCCATGAACAGAAAGCCCAGGATGTAC-AGGATGTACAGAAGTCCAGATGTTCC-TAGAGGATGT----GAAGGCCCATGTAAGGTCCAGTCATTTGAGTCCA-GACATGATATCCAGCACATT-GGTA-AAGTCATGTGTGTTAGTGATGTTACTCGTGGTATTGGGCTTACCCACAGGGTAGG---CAAGAGATTTTGTGTTA-AGTCC----GTTTATGTTCTGGGTAAGATCTGGATGGATGAGAACATTAAAACTAAGAATCATACGAATAGTGTTATGTTTTTTC-TTGTTAGAGATCGTAGGCCCGTTGACAAG---CCTCAAGATTTTGGC-GAGGTTTTTAACATGTTTGATAATGAGCCCAGCACGGCGACTGTGAAGAATGTTCATCGTGATAGATACCAGGT-ATTAAGGA---AGTGGTACGCA-----ACTGTGACAGGCGGTCAATATGCATCTAAGGAGCAGGCTCTCGTGAAGAAG-TTTATTAGGGTTA-ATAATTATGT-TGTGTACAACCAG--------CAA-GAGGCTGGGAAGTATGAGAATCATACTGAGAATGCGTTGATGTTGTATATGGCGTGTACGCACGCCTCTAACCCTGTGTATGCTACATTGAAGATACGGATCTACTTCTATGATTCAG-------TATCGAA------------------------TTAATAAATATTAAATTTTATTGAATATGATTGTTCTATATTTAC-----AACATGATGTAATACATTCCATAATACATGATCAACTGCTCTAATTACATTGTTAATACTGATAACTCCTAAATTATCTAAATACTTAAGTAC-------TTGGGTCTTAAAG---------------ACCCTTAAGAAACGA-------------CCAGTCGGAG-GCTGTGAGGTC-----------ATCCAGATTC-TGAAGGCTAGGAAAC-ATTTGTGTATCCCC------AACGCTTT----------CCTCAGGTTGTGATTGAACTGTATCTGGACGGTTATGATGTCTTGGTTC--------ATGGTGAATGGCCTGTTGTGGTGCTCTGTTATCTTGAAATAGAGGGGATTTTGAATCTCCC--------AGATA------------AACACGCCATTCTCTGCTTGAGCT--GCAGTGATGAGTTCCCCGGTGCG--TGAATCCATGACTGTGACAGGCTAGTGCTATGAAA-------TAAGAACATCCACAAGGGAGATC-AACACGTCGACGCC---TGGTCCCCTTCTTGGCTAGCCTGTGCTGCACTTTGA----TTGGAACCTGGGTAGAGTGGGCCTTCGAGGGTGACGAAGAT---------------------------CGCATTCTTTAAAGC------CCAATTTTTGAGTGCAGAATTCTTCTCTTCGTCCAAGAACTCTTTATAGCT-TGAATTGGGTCCTGGATTGCAGAGGAAGATA-------------------------------------GTGGGAATTCCGCCTTTAA-TTTGAACTGGCTTTCCGTATTTTGTGTTGCTTTGCCAGTCCCTT-----------------------TGGGCCCCCATGAATTCTTTGAAGTG--------CTTTAGATAATGCGGATCGACGTCATCAATGACG-TTG----------------TACCAGGCCTC-ATTACTGTAGAC---CTTTGGACTAAGGTCTAAGTGACCACACAAATAATTATGTGGACCCAGTGACCTAGCCCACATCGTCTTCCCCGT-------ACGACTATCGCCCTCT-----------ATGACAATACTTTTAGGTCTCAATGG-CCGCGCAGCGGCATCCATCACGTTTTCAGCAGCCCAGACTTCAAGTTCTTCCGGAACTTGATCAAAAGAAGAAGAAGA------------------------------AAAAGGAGATACATAAACCTCCACAGGAGGTGTAAAAAT-CCTATCTAAATTAGC---ATTTAAATTATGAAATTGTAATACATAATCTTTTGGAGCTAACTCCTTAATGACT-CTAAG--AGCCTCTGACTTACTGCCTGCGTTAAGCGCTGCGGCGTAAGCGT-CGTTGGCTGATTGTT----GCCCCCCTCTAGCAGATCTTCCATCGATCTGAAACTCTCCCCATTCGAGGGTGTCTCCGTCCTTCTCCAGATAGGACTTGACGTCGGAGCTTGA---TTTAGCT-----CCCTGAATGTTCGGATGGAAAT--GTGCTGACCTGGTTGGGGAGACCAGGTCGAAGAATCG-CTGATTTTGGCACCTGTATTTCCCTTCGAACTGGAT-GAGCACGTGCAGAT---GAGGGCTCCCATCTTCGTGGAGTTCCC-TGCAGATCT--------TGATGAATTTTTTATTGGTGGG--TGTATCTAGGGCTTGTATTTGGGAAAGTGCTTCTTCTTTAGTAAGAGA----GCAGTGTGGGTAAGTGAGGAAATAATTTCTGGCATATATTTGAAAACGCTTAGGAGGAGCC--ATTGAC-TTGGT--CAAATG-GGTA---CCAATTG---GG------TTCTGGATGTTTA---TCACCTGTATATCGGTACT------CAA----------TATATA--GTGAG-TACCAAATGGCATAA-TTGTAA--------TAAA-AGAACTCTAATTT-----GAAATTCAAA----CGAAAAGGCT------AAA--GC-GGCCATGCCCGTC-TAATATT

>PedLCV-INNDCarrot16KX168427

-------ACCGGATGGCCGCGC----TTTTT------TTATGCCCCCCAC--AGAGCACTAACTGACA----ATGACATGTGGACCAATGAGA-ATGGTTCCTC-----------ATAGCCTAATTATTT-C-ATGGTCCCCCC----TATAA--ACTTAGTGCGCAAGT-TGTG---TTTCATATTC-----ACT-ATGTGGGATCCGTT------ATTGAACGAGTTTCCCGAAACCGTTCACGGTTTTAGGTGTATGTTAGCAGTT--------AAATATTTGCA-------GTTAGTAGAAAGTACGTA-TTCCCCAGATAC---TCTGGGATTCGAT--TTAATTCGTGATTTAATTTCAGTGATAAGGGCTAGGAATTATGTCGAAGCGACCAGCAGATATAATCATTTCCACGCCCGCCTCGAAGGTACGCCGCCGTCTCAACTTCGACAGCCCATATGCGAGCCGTGCTGCTGCCCCCATT---GTCCGCG-----------------TCACAAAGGCAAG---GGCATGGGCGAACAGGCCCATGAACAGAAAGCCCAGGATGTAC-AGGATGTACAGAAGTCCAGATGTTCC-TAGAGGATGT----GAAGGCCCATGTAAGGTCCAGTCATTTGAGTCCA-GACATGATATTCAGCACATT-GGTA-AAGTCATGTGTGTTAGTGATGTTACCCGTGGTATTGGGCTTACCCACAGGGTAGG---CAAGAGATTTTGTGTTA-AGTCC----GTTTATGTTCTGGGTAAGATCTGGATGGATGAGAACATTAAAACTAAGAATCATACGAATAGTGTTATGTTTTTTC-TTGTTAGAGATCGTAGGCCCGTTGACAAG---CCTCAAGATTTTGGC-GAGGTTTTTAACATGTTTGATAATGAGCCTAGCACGGCGACTGTGAAGAATGTTCATCGTGATAGATACCAGGT-ATTAAGGA---AGTGGTACGCA-----ACTGTGACAGGCGGTCAATATGCATCGAAGGAGCAGGCTCTCGTGAAGAAG-TTTATTAGGGTTA-ATAATTATGT-TGTGTACAACCAG--------CAA-GAGGCTGGGAAGTATGAGAATCATACTGAGAATGCGTTGATGTTGTATATGGCGTGTACGCACGCCTCTAACCCTGTGTATGCTACATTGAAGATACGGATCTACTTCTATGATTCAG-------TATCGAA------------------------TTAATAAATATTAAATTTTATTGAAGATGATTGTTCTATATTTAC-----AACATGATGTAATACATTCCATAATACATGATCAACTGCTCTAATTACATTGTTAATACTGATAACTCCTAAATTATCTAAATACTTAAGCAC-------TTGGGTCTTAAAG---------------ACCCTTAAGAAACGA-------------CCAGTCGGAG-GCTGTGAGGTC-----------ATCCAGATTC-TGAAGGCTAGGAAAC-ATTTGTGTATCCCC------AACGCTTT----------CCTCAGGTTGTGATTGAACTGTATCTGGACGGTTATGATGTCTTGGTTC--------ATGGTGAATGGCCTGTTGTGGTGCTCTGTTATCTTGAAATAGAGGGGATTTTGAATTTCCC--------AGATA------------AACACGCCATTCTCTGCTTGAGCT--GCAGTGATGAGTTCCCCGGTGCG--TGAATCCATGATCGTGACAGGCTAGTGCTATGAAA-------TAAGAACATCCACAAGGGAGATC-AACACGTCGACGCC---TGGTCCCCTTCTTGGCTAGCTTGTGCTGCACTTTGA----TTGGAACCTGAGTAGAGTGGGCCTTCGAGGGTGACGAAGAT---------------------------CGCATTCTTTAAAGC------CCAATTTTTGAGTGCAGAATTTTTTTCTTCGTCCAAGAACTCTTTATAGCT-TGAATTGGGTCCTGGATTGCAGAGGAAGATA-------------------------------------GTGGGAATTCCGCCTTTAA-TTTGAACTGGCTTTCCGTATTTCGTGTTGCTTTGCCAGTCCCTT-----------------------TGGGCCCCCATGAATTCCTTAAAGTG--------CTTTAGGTAGTGGGGATCGACGTCATCAATGACG-TTG----------------TACCAGGCCTC-ATTACTGTAGAC---CTTTGGACTAAGGTCTAAGTGACCACACAAATAATTATGTGGACCCAGTGACCTAGCCCACATCGTCTTCCCCGT-------ACGACTATCGCCCTCT-----------ATGACAATACTTTTAGGTCTCAATGG-CCGCGCAGCGGCATCCATCGCGTTTTCAGCAGCCCAGACTTCAAGTTCTTCCGGAACTTGATCAAAAGAAGAAGAAGA------------------------------AAAAGGAGATACATAAACCTCCACAGGAGGTGTAAAAAT-CCTATCTAGATTAGC---ATTTAAATTATGAAATTGTAATACATAATCTTTTGGAGCTAACTCCTTAATGACT-CTAAG--AGCCTCTGACTTACTGCCTGCGTTAAGCGCTGCGGCGTAAGCGT-CGTTGGCTGATTGTT----GCCCTCCTCTAGCAGATCTTCCATCGATCTGAAACTCTCCCCATTCGAGGGTGTCTCCGTCCTTCTCCAGATAGGACTTGACGTCGGAGCTTGA---TTTAGCT-----CCCTGAATGTTCGGATGGAAAT--GTGCTGACCTGGTTGGGGAGACCAGGTCGAAGAATCG-CTGATTTTGGCACCTGTATTTCCCTTCGAACTGGAT-GAGCACGTGCAGAT---GAGGGCTCCCATCTTCGTGGAGTTCCC-TGCAGATCT--------TGATGAATTTTTTATTGGTGGG--TGTATCTAGGGCTTGTATTTGGGAAAGTGCTTCTTCTTTAGTAAGAGA----GCAGTGTGGGTAAGTGAGGAAATAATTTCTGGCATTTATTTGAAAACGCTTAGGAGGAGCC--ATTGAC-TTGGT--CAAATG-GGTA---CCAATTG---GG------TTCTGGATGTTTA---TCACCTGTATATCGGTACT------CAA----------TATATA--GTGAG-TACCAAATGGCATAA-TTGTAA--------TAAA-AGAACTCTAATTT-----GAAATTCAAA----CGAAAAGGCT------AAA--GC-GGCCAT--CCGTC-TAATATT

>PedLCV-INLucCrape11JN807764

-------ACAGGACGGCCGCGC----CATTT------TTATGGCCCCCCC--ACCGCATTAACTGTCT----GTAACATATGGGCCCATGAGA-ATGGTCCCTC-----------CTAGCCTAATTATTT-A-ATGGTCCCCCC----TATAA--ACTTAGTGCGCAAGT-TGTG---TTTCACGCTT-----ACCAATGTGGGATCCATT------AGTG-ACGAGTTTCCCGAAACCGTTCACGGTTTTAGATGTATGTTAGCAGTT--------AAATATTTGCA-------GCTCGTAGAAAATACGTA-TTCCCCAGAGAC---TAAGGGCTACGAT--TTAATTAGGGATTTAATTTCAGTAATAAGGGCTAGGAATTATGTCGAAGCGACCAGCAGATATAATCATTTCCACGCCCGCTTCGAAGGTACGTCGCCGTCTCAACTTCGACAGCCCATATGCGAGCCGTGCTGCTGCCCCCATT---GTCCGCG-----------------TCACCAAGGCAAG---GGCATGGGCGAACAGGCCCATGAACAGAAAGCCCAGGATGTAC-AGGATGTACAGAAGTCCAGATGTTCC-TAGAGGATGT----GAAGGCCCATGTAAGGTCCAGTCATTTGAGTCCA-GACATGATATCCAGCACATT-GGTA-AAGTCATGTGTGTTAGTGATGTTACTCGTGGTATTGGGCTTACCCACAGGGTAGG---CAAGAGATTTTGTGTTA-AGTCC----GTTTATGTTCTGGGTAAGATCTGGATGGATGAGAACATTAAAACTAAGAATCATACGAATAGTGTTATGTTTTTCC-TTGTTAGGGATCGTAGGCCTGTTGATAAG---CCTCAAGAGTTTGGT-GAGGTTTTTAATATGTTTGATAATGAGCCCAGTACGGCGACAGTGAAGAATGTGCATCGTGATAGGTACCAGGT-GCTCAGGA---AGTGGCACGCC-----ACTGTGACAGGCGGTCAATATGCATCGAAGGAGCAGGCTCTCGTGAAGAAG-TTTATTAGGGTTA-ATAATTATGT-TGTGTATAACCAG--------CAA-GAGGCTGGCAAGTATGAGAATCATACTGAGAATGCATTGATGTTGTATATGGCGTGTACGCACGCCTCTAACCCTGTGTATGCTACATTGAAGATACGGATCTATTTCTATGATTCAG-------TATCGAA------------------------TTAATAAAGATTATATTTTATTGAAGACGATTGTTCTACATTCAC-----AATATGATGTAATACATTCCATAATACATGATCAACTGCTCTAAGTACATTATTAATACTGATAATTCCTATATTATCTAAATACTTAAGAAC-------TTGGGTCCTAAAG---------------ACAGTTAAGAAACGA-------------CCAGTCGGAG-TCTGTGAGGTC-----------ATCCAGATTC-GGAAGGCTAGGAAAC-ATTTGTGTATCCCC------AACGCTTT----------CCTCAGGTTGTGATTGAACTGTATCTGGACGGTGATGATGTCGTGGTTC--------ATTAGGAATGGCCGGTTGTGGTGCTCTGTTATCTTGAAATACAGGGGATTTGGAATCTCCC--------AGATA------------AACACGCCATTCTCTGCTTGAGCT--GCAGTGATGAATTCCCCTGTGCG--TAAATCCATGATTGTGGCAGGCTAGTGCTATGAAG-------TACGAGCAGCCGCAGTGTAGATC-AACTCTCCGACGCC---TGGTCCCCTTCTTGGCTAGCCTGTGCTGCACTTTGA----TTGGAACCTGAGTAGAGTGGCCCTTCGAGGGTGATGAAGGT---------------------------CGCATTCTTTAAAGC------CCAATTTTTGAGTGCAGAGTGCTTCTCATCATCCAAGAACTCTTTATAGCT-TGAATTGGGTCCTGGATTGCAGAGGAAGATA-------------------------------------GTGGGAATTCCACCTTTAA-TTTGAACTGGCTTCCCGTACTTTGTATTTGATTGCCAGTCCCTT-----------------------TGGGCCCCCATGAATTCCTTAAAGTG--------CTTTAGGTAATGCGGATCGACGTCATCAATGACG-TTG----------------TACCAGGCATC-ATTATTGTAGAC---CTTTGGGCTTAGGTCGAGGTGTCTACACAAATAATTATGTGGACCCAGTGACCTAGCCCACATCGTCTTCCCCGT-------ACGACTATCGCCCTCT-----------ATCACTATACTTATGGGTCTCAATGG-CCGCGCAGCGGAACCTACCACATTTTCAGCAGCCCATTCCTCAAGTTCATCTGGAACTTGATCAAAAGAAGAAGAACA------------------------------GAAAGGAGAAACATAAACCTCTACCGGAGGTGTAAAAAT-CCTATCTAAATTAGC---ATTTAAATTATGAAATTGTAATACATAATCTTTGGGAGCTATCTCCTTAATGACT-CTGAG--AGCCTCTGATTTACTGCCTGTGTTAATTGCTGCGGCGTATGCGT-CGTTGGCTGACTGTT----GACCCCCTCTAGCAGATCTTCCGTCGATCTGAAACTCTCCCCAATCAAGGGTGTCTCCGTCCTTCTCCATATAGGACTTGACATCGGAACTTGA---TTTAGCT-----CCCTGAATGTTCGGATGGAAAT--GTGCTGACCTGGTTGGGGAAACCAGGTCGAAGAATCT-GTTATTCTGGCATTTGTATTTCCCTTCGAACTGGAT-AAGCACGTGGAGAT---GAGGAGACCCATCTTCGTGAAGCTCTC-TGCAGATTT--------TGATGAATTTTTTATTGGTGGG--TGTGGAGAGGTTTTGAAATTGGGAAAGTGCTTCTTCTTTGGTGAGTGA----GCACTGTGGATATGTGAGGAAATAATTTTTGGCATATATTAAAAAACGTTTTGGAGGAGCC--ATTGAC-TTGGT--CAAATG-GGTA---CCAATTG---GG------TTCTGGATGTTTA---TCACCTGTATATCGGTACT------CAA----------TATATA--GTGAG-TACCAAATGGCATAA-TTGTAA--------TAAA-AGAACTCTAATTT-----GAAATTCAAA----CGAAAAGGCT------AAA--GC-GGCCAT--CCGTC-TAATATT

>PedLCV-INLucCestrum11JQ012790

-------ACCGGACGGCCGCGC----CATTT------TTATGGCCCCCCC--ACCGCATTAACTGTCT----GTAACATATGGGCCCATGAGA-ATGGTCCCTC-----------CTAGCCTAATTATTT-A-ATGGTCCCCCC----TATAA--ACTTAGTGCGCAAGT-TGTG---TTTCACGCTT-----ACCAATGTGGGATCCATT------AGTG-ACGAGTTTCCCGAAACCGTTCACGGTTTTAGATGTATGTTAGCAGTT--------AAATATTTGCA-------GCTCGTAGAAAATACGTA-TTCCCCAGAGAC---TAAGGGCTACGAT--TTAATTAGGGATTTAATTTCAGTAATAAGGGCTAGGAATTATGTCGAAGCGACCAGCAGATATAATCATTTCCACGCCCGCTTCGAAGGTACGTCGCCGTCTCAACTTCGACAGCCCATATGCGAGCCGTGCTGCTGCCCCCATT---GTCCGCG-----------------TCACCAAGGCAAG---GGCATGGGCGAACAGGCCCATGAACAGAAAGCCCAGGATGTAC-AGGATGTACAGAAGTCCAGATGTTCC-TAGAGGATGT----GAAGGCCCATGTAAGGTCCAGTCATTTGAGTCCA-GACATGATATCCAGCACATT-GGTA-AAGTCATGTGTGTTAGTGATGTTACTCGTGGTATTGGGCTTACCCACAGGGTAGG---CAAGAGATTTTGTGTTA-AGTCC----GTTTATGTTCTGGGTAAGATCTGGATGGATGAGAACATTAAAACTAAGAATCATACGAATAGTGTTATGTTTTTTC-TTGTTAGGGATCGAAGGCCCGTTGATAAG---CCTCAGGATTTTGGT-GAGGTTTTTAATATGTTTGATAATGAGCCCAGCACGGCGACTGTGAAGAATGTTCATCGTGATAGGTACCAAGT-GTTAAGGA---AGTGGCACGCA-----ACTGTGACTGGCGGTCAATATGCATCGAAGGAGCAGGCTCTCTTGAAGAAG-TTTATTAGGGTTA-ATAATTATGT-TGTGTACAACCAG--------CAA-GAGGCTGGGAAGTATGAGAATCATACTGAGAATGCTTTGATGTTGTATATGGCGTGTACGCACGCCTCTAACCCTGTGTATGCTACATTGAAGATACGGATCTACTTCTACGATTCAG-------TATCGAA------------------------TTAATAAATATTGAATTTTATTGAAGATGATTGGTCTACATATAC-----AACATGCTCTAATACATTCCATAATACATGATCAACTGCCCTAATTACATTGTTAATACAGATAACTCCTAAGTTATCTAAATATTTAATAAC-------TTGAGTCTTAAAG---------------ACCCTTAAGAAATGA-------------CCAGTCGGAG-GCTGTGAGTTC-----------GTCCAGATTC-GGAAGACTAGGAAGC-ATTTGTGTATCCCC------AACGCTTT----------CCTCAGGTTGTGATTGAACTGTATCTGGACTGTGATGATGTCTTGGTTC--------ATCAGGAATGGCCTGTTGTGGTGCTCGGATATCTTGAAATACAGGGGATTTCGTATCTCCC--------AGATA------------TACACGCCACTCTCTGCTTGAGCT--GCAGTGATGATATCCCCTGTGCG--TGAATCCATGGTTGTGGCAGGCTAATGCTATGAAG-------TATGAACACCCACACGGGAGATC-AACTCGTCGACGCC---TGGTCCCCCTCTTGGCCAGCCTGTGCTGCACTTTGA----TTGGAATCTGAGTAGAGTGGGCCTTCGAGGGTGATGAAGGT---------------------------GGCATTCTTTAAAGC------CCAATTTTTAAGTGCAGCATTTTTCTCTTCATCCAAGAACTCTTTATAGCT-GGAATTGGGTCCTGGATTGCAGAGGAAGATA-------------------------------------GTGGGAATGCCACCTTTAA-TTTGAACTGGCTTTCCGTATTTTGTGTTTGATTGCCAGTCTCTT-----------------------TGGGCCCCCATGAATTCTTTAAAGTG--------CTTTAGGTAGTGGGGATCGACGTCTTCAATGACG-TTG----------------TACCAGACATC-ATTATTGTAGAC---CTTTGGGCTAAGGTCCAGATGACCACACATATAATTATGTGGACCCAGTGACCTAGCCCACATCGTCTTCCCCGT-------ACGACTATCGCCCTCT-----------ATCACTATACTTATGGGTCTCAATGG-CCGCGCAGCGGAACCTACCACATTTTCAGCAGCCCATTCCTCAAGTTCATCTGGAACTTGATCAAAAGAAGAAGAACA------------------------------GAAAGGAGAAACATAAACCTCTACCGGAGGTGTAAAAAT-CCTATCTAAATTAGC---ATTTAAATTATGAAATTGTAATACATAATCTTTGGGAGCTATCTCCTTAATGACT-CTGAG--AGCCTCTGATTTACTGCCTGTGTTAATTGCTGCGGCGTATGCGT-CGTTGGCTGACTGTT----GACCCCCTCTAGCAGATCTTCCGTCGATCTGAAACTCTCCCCAATCAAGGGTGTCTCCGTCCTTCTCCATATAGGACTTGACATCGGAACTTGA---TTTAGCT-----CCCTGAATGTTCGGATGGAAAT--GTGCTGACCTGGTTGGGGAAACCAGGTCGAAGAATCT-GTTATTCTGGCATTTGTATTTCCCTTCGAACTGGAT-AAGCACGTGGAGAT---GAGGAGACCCATCTTCGTGAAGCTCTC-TGCAGATTT--------TGATGAATTTTTTATTGGTGGG--TGTGGAGAGGTTTTGAAATTGGGAAAGTGCTTCTTCTTTGGTGAGTGA----GCACTGTGGATATGTGAGGAAATAATTTTTGGCATATATTAAAAAACGTTTTGGAGGAGCC--ATTGAC-TTGGT--CAAATG-GGTA---CCAATTG---GG------TTCTGGATGTTTA---TCACCTGTATATCGGTACT------CAA----------TATATA--GTGAG-TACCAAATGGCATAA-TTGTAA--------TAAA-AGAACTCTAATTT-----GAAATTCAAA----CGAAAAGGCT------AAA--GC-GGCCAT--CCGTC-TAATATT

>PedLCV-INBihTb10GU732204

-------ACCGGATGGCCGCGC----TTTTT------TTGTGGCCCCCAC--AAAGCACTAACTGACA----ATGACATGTGGTCCAATGAGA-ATCGTTCCTC-----------GTAGCCTAATTGTTT-T-GTGGTCCCCTC----TATAA--ACTTAGTGCGCAAGT-TGTG---CTTCACATTC-----AAT-ATGTGGGATCCGTT------ATTGAATGAGTTTCCCGAAACCGTTCACGGTTTTAGGTGTATGTTAGCAGTT--------AAATATCTGCA-------GTTAGTAGAAAATACGTA-TTCCCCAGATAC---TCTGGGATACGAT--TTAATTAGGGATTTGATTTCAGTAATAAGGGCCAGGAATTATGTCGAAGCGACCAGCAGATATAATCATTTCCACGCCCGCTTCGAAGGTACGTCGCCGTCTCAACTTCGACAGCCCATATGTGAGCCGTGCTGCTGCCCCCATT---GTCCGCG-----------------TCACAAAGGCAAA---AGCATGGGCGAACAGACCCATGAACAGAAAGCCCAGGATGTAC-AGGATGTACAGAAGTCCAGATGTTCC-TAGAGGATGT----GAAGGCCCATGTAAGGTTCAGTCCTTTGAGTCCA-GACATGATATCCAGCATATT-AGTA-AAGTCATGTGTGTCAGTGATGTTACTCGTGGAACTGGGCTTACCCATAGAGTGGG---TAAGAGATTTTGTGTGA-AGTCT----GTGTATGTTTTGGGTAAGATATGGATGGATGAGAACATTAAGACCAAGAATCACACGAATAGTGTGATGTTTTTTC-TAGTTAGAGATCGTAGACCAGTTGATAAA---CCTCAAGATTTTGGA-GAGGTGTTTAACATGTTTGATAATGAGCCCAGTACGGCGACTGTGAAGAATGTTCATCGTGATAGGTATCAAGT-GCTGCGCA---AATGGTATGCA-----ACTGTCACCGGTGGACAATACGCTTCAAAGGAACAAGCTCTCGTGAAGAAG-TTTATTAGGGTTA-ATAATTATGT-TGTGTATAACCAG--------CAA-GAAGCTGGCAAGTATGAGAATCATTCTGAGAATGCTTTAATGTTGTATATGGCGTGTACTCACGCCTCTAACCCAGTGTATGCTACTTTGAAAATACGGATCTACTTCTATGATTCCG-------TATCAAA------------------------TTAATAAATATTGAATTTTATTGAAGATGATTGGTCTACATATAC-----AACATGCTCTAATACATTCCATAATACATGATCGACTGCTCTAATTACATTATTAATACTGACAATTCCTAGATTATTTAAATACTTAAGCAC-------TTGGGTCTTAAAG---------------ACCCTTAAGAAACGA-------------ACAGTCGGAG-GGTGTGAGGTC-----------ATCCAGATTC-GGAAGGCTAGGAAAC-ATTTGTGTATCCCC------AACGCTTT----------CCTCAGGTTGTGATTGAACTGTATCTGGACGGTGATGATGTCTTGGTTC--------ATTAGGAATGGCCTGTTGTGGTGCTCTGTTATCTTGAAATACAGGGGATTTTGAATCTCCC--------AGATA------------AACATGCCATTCTCTGCTTGAGCT--GCAGTGATGAGTTCCCCTGTGCG--TGAATCCATGGTCGTGGCAGGCTAATGCTATGAAG-------TATGAACACCCACAAGGTAGATC-AACTCTCCGACGTC---TGGTCCCCTTCTTGGCTAGCCTGTGCTGCACTTTGA----TTGGAACCTGAGTAGAGTGGGCCTTCGAGGGTGACGAAGAT---------------------------CGCATTCTTTAAAGC------CCAATTTTTGAGTGCAGAATTCTTCTCTTCGTCTAAATACTCTTTATAGCT-TGAATTGGGTCCTGGATTGCAGAGGAAGATA-------------------------------------GTGGGAATGCCACCTTTAA-TTTGAACTGGCTTTCCGTACTTTGTATTTGATTGCCAGTCTTTT-----------------------TGGGCCCCCATGAATTCTTTAAAGTG--------CTTTAAGTAGTGGGGATCGACGTCATCAATGACG-TTG----------------TACCAGGCCTC-ATTACTGTACAC---CTTTGGACTAAGGTCTAAATGACCACACAAATAATTATGTGGACCCAGTGACCTGGCCCACATTGTCTTCCCCGT-------CCTACTATCACCCTCA-----------ATCACTACACTTTTAGGTCTCATTGGCCCGCGCAGCGGCACTGACGACGTTCTCGGCAGCCCATTCCTCAAGTTCTTCCGGAACTTGATGTAAAGAAGAAGAAGA------------------------------AAAAGGAGAAACATAAACCTCCATGGGAGGTGTATAAAT-CCTATGTAAATTAGC---ATATAAATTATGATATTGAAGTACATAATCTTTTGGGGCGAATCCTTT-ATGATT-CTAAG--AGCCTCTGACTTACTGCCTGCGTTAAGCGCTGCGGCGTAAGCGT-CGTTGGCTGTCTGTT----GTCCCCCTCTAGCAGATCGTCCGTCGATCTGAAACTCTCCCCAGTCGAGGGTGTCTCCGTCCTTCTCCATATAGGACTTGACATCGGAGCTGGA---TTTAGCT-----CCCTGGATGTTCGGATGGAAAT--GTGCTGACCTGGTTGGGGATACGAGGTCGAAGAATCG-TTGATTTTTGCACTTGTATTTGCCTTCGAACTGGAT-GAGCACGTGAAGAT---GAGGTTCCCCATTTTCATGAAGCTCTC-TGCAGATTT--------TAATGTATTTTTTGTTTACTGG--GGTTTGTAGGTTTTGTAGTTGGGAAAGTGCTTCCTCTTTAGTAAGGGA----GCATTTGGGATAAGTGAGGAAATAATTTTTGGCATTAATCTGGAAATGCTTTGGGGGCA-T--GTTGAC-TTGGT--CAG--A-GGAC---CCGATTGAACTA------TCTTG--CAACTC---TCCCCTGTATATCGGGTCC------CTA----------TATATA--GTGGG-ACTGAAATGGCAATA-TTGTAA--------TTAC-GATAA-GAAATTC-----AAAATCCTCA----C------GCT--CCAATCA--GC-GGCCAT--CCGTA-TAATATT

>PaLCV-PKMI07FM955601

-------ACCGGATGGCCGCGA-TTTTTTTT------TTGTGGCCCCCAC--AAAGCACTAACTGACA----ATGACATGTTGACCAATGAGA-ATCGTTCCTC-----------GTAGCCTAATTGTTT-C-GTGGTCCCCCC----TATAA--ACTTAGTGCGCAAGT-TGTG---TTTTCCATTC-----ACT-ATGTGGGATCCGTT------ATTGAACGAGTTTCCCGAAACCGTTCACGGTTTTAGGTGTATGTTAGCAGTT--------AAATATCTGCA-------GTTAGTAGAAAATACGTA-TTCCCCAGATAC---TCTGGGATACGAT--TTAATTAGGGATTTGATTTCAGTAATAAGGGCTAGGAATTATGTCGAAGCGACCAGCAGATATAATCATTTCCACGCCCGTTTCGAAGGTACGCCGCCGTCTCAACTTCGACAGTCCATACGTGAGCCGTGCTGCTGCCCCCATT---GTCCGCG-----------------TCACCAAAGCAAA---AGCATGGGCGAACAGGCCCATGAACAGAAAGCCCAGGATGTAC-AGGATATACAGAAGTCCAGATGTTCC-TAGAGGATGT----GAAGGCCCATGTAAGGTCCAGTCCTTTGAGTCCA-GACATGATATCCAGCATATA-GGTA-AAGTCATGTGTGTCAGTGATGTTACTCGTGGAACTGGGCTGACCCATAGAGTGGG---TAAGAGATTTTGTGTCA-AGTCT----GTGTATGTGTTGGGCAAGATTTGGATGGATGAGAACATCAAGACCAAGAATCATACGAATAGTGTTATGTTTTTTT-TAGTTAGAGACCGTAGACCAGTTGACAAG---CCTCAGGATTTTGGA-GAGGTTTTTAACATGTTTGATAATGAGCCCAGTACGGCGACTGTGAAGAATGTGCATCGTGATAGGTACCAGGT-TCTGCGCA---AATGGTATGCA-----ACTGTCACCGGTGGACAATATGCGTCGAAGGAACAAGCTCTCGTGAAGAAG-TTTATTAGAGTTA-ATAATTATGT-TGTGTATAACCAG--------CAA-GAAGCTGGCAAGTATGAGAATCATTCTGAGAATGCGTTAATGTTGTATATGGCGTGTACTCACGCCTCTAATCCAGTGTATGCTACTTTGAAGATACGGATCTACTTCTATGATTCCG-------TGACAAA------------------------TTAATAAATATTGAATTTTATTGAAGATGATTGGTTTACATATAC-----AACATGCTCTAATACATTCCATAATACATGATCAACTGCTCTAACTACATTATTAATACTGATAACTCCTAGATTATCTAAATACTTAAGCAC-------TTGGGTCTTAAAG---------------ACCCTTAAGAAACGA-------------CCAGTCGGAG-GCTGTGAGGTC-----------ATCCAGATTC-GGAAGGCTAGGAAAC-ATTTGTGTATCCCC------AACGCTTT----------CCTCAGGTTGTGATTGAACTGTATCTGGACAGTGATGATGTCTTGGTTC--------ATGAGGAATGGTCTGTTGTGGTGCTCTGTTATCTTGAAATAGAGGGGATTTTGAATCTCCC--------AGATA------------AATACGCCATTCTGTGCTTGAGCT--GCAGTGATGAGTTCCCCTGTGCG--TGAATCCATGGTTGTGGCAGGCTAATGCTATGAAG-------TATGAACACCCACACGGGAGATC-AACACGACGACGCC---TGGTCCCCTTCTTGGCTAGCCTGTGCTGCACTTTGA----TTGGAACCTGAGTAGAGTGGGCCTTCGAGGGTGATGAAGGT---------------------------CGCATTCTTTAATGC------CCAATTTTTAAGTGCAGAATTCTTCTCCTCATCCAAAAACTCTTTATAGCT-TGAGTTGGGTCCTGGATTGCAGAGGAAGATA-------------------------------------GCGGGAATTCCGCCTTTAA-TTTGAACTGGCTTTCCGTACTTTGTATTTGATTGCCAGTCCCTT-----------------------TGGGCCCCCATGAATTCTTTAAAGTG--------CTTTAGGTAGTGGGGATCGACGTCATCAATGACG-TTG----------------TACCAGGCCTC-GTTGCTGTAGAC---CTTTGGACTAAGGTCTAAATGACCACACAGATAATTGTGTGGACCCAGTGACCTGGCCCACATCGTCTTCCCCGT-------TCTACTATCACCCTCT-----------AAGACAATACTTTTAGGTCTCAATGG-CCGCGCAGCGGCATCCACCACGTTCTCAGCAGCCCAGACTTCAAGTTCTTCCGGAACTTGATCAAAAGAAGAAGAAGA------------------------------AAAAGGAGAAACATAAACCTCCACAGGAGGTGTAAAAAT-CCTATCTAAATTACA---TTTTAAATTATGATATTGAAAAATAAAATCTTTAGGGAGTTTTTCCCTAATTATTGCTAAAGCAGCGTCAGCCGAA---CCTGCATTTAGGGCCTCTGCTGCTGCAT-CATTAGCTGTCTGTT----GACCTCCTCTAGCAGATCTTCCATCGATCTGAAACTGACCCCAGTCGATGTAATCACCGTCCTTCTCGATGTAGGACTTGACATCTGAGCTGGA---CTTAGCT-----CCCTGGAAGTTTGGGTGGAATT--GGGTGGAGGTATTAGGGTGAGTGACATCGAAATGTCT-GGGGTTTCTGAACTTGGATTTACCTTTGAACTGGAT-GAGGGCATGGATAT---GCAGAGACCCATCTTGGTGTTTTTCTT-GTGATACTC--------TGATAAACAATTTATCAGAAGG--ACAGGTAATGTTTTTAAGTAGTTCGAGCATTTGCTCTTTCGGTATTGG----GCATTTTGGATAAGTAAGGAAGATATTTTTGGCATTTATACAGAAAGAGTTATTACGAGGCATATTGAA-TTGGGGACACTCA-AAAC---TCTGAGGAATGG-----------------------------------GGGACT------CGGGGGACGCATTTATATG--GTG-T-CCCCAAATGGCAATT-TGGTAA--------TTCA-GAAAG-AAATTTC-----AAAATCTCCA----C------GCT--CCAAAAA--GC-GGCCAT--CCGTA-TAATATT

>PaLCuV-PKMI07FM955602

-------ACCGGATGGCCGCGA-TTTTTTTT------TTGTGGCCCCCAC--AAAGCACTAACTGTCA----ATGACATGTTGACCAATGAGA-ATCGTTCCTC-----------GTAGCCTAATTGTTT-C-GTGGTCCCCCC----TATAA--ACTTAGTGCGCAAGT-TGTG---TTTTCCATTC-----ACT-ATGTGGGATCCGTT------ATTGAACGAGTTTCCCGAAACCGTTCACGGTTTTAGGTGTATGTTAGCAGTT--------AAATATCTGCA-------GTTAGTAGAAAATACGTA-TTCCCCAGATAC---TCTGGGATACGAT--TTAATTAGGGATTTGATTTCAGTAATAAGGGCTAGGAATTATGTCGAAGCGACCAGCAGATATAATCATTTCCACGCCCGTTTCGAAGGTACGCCGCCGTCTCAACTTCGACAGTCCATACGTGAGCCGTGCTGCTGCCCCCATT---GTCCGCG-----------------TCACCAAAGCAAA---AGCATGGGCGAACAGGCCCATGAACAGAAAGCCCAGGATGTAC-AGGATGTACAGAAGTCCAGATGTTCC-TAGAGGATGT----GAAGGCCCATGTAAGGTCCAGTCCTTTGAGTCCA-GACATGATATCCAGCATATA-GGTA-AAGTCATGTGTGTCAGTGATGTTACTCGTGGAACTGGGCTGACCCATAGAGTGGG---TAAGAGATTTTGTGTCA-AGTCT----GTGTATGTGTTGGGCAAGATTTGGATGGATGAGAACATCAAGACCAAGAATCATACGAATAGTGTTATGTTTTTTT-TAGTTAGAGACCGTAGACCAGTTGACAAG---CCTCAGGATTTTGGA-GAGGTTTTTAACATGTTTGATAATGAGCCCAGTACGGCGACTGTGAAGAATGTGCATCGTGATAGGTACCAGGT-TCTGCGCA---AATGGTATGCA-----ACTGTCACCGGTGGACAATATGCGTCGAAGGAACAAGCTCTCGTGAAGAAG-TTTATTAGAGTTA-ATAATTATGT-TGTGTATAACCAG--------CAA-GAAGCTGGCAAGTATGAGAATCATTCTGAGAATGCGTTAATGTTGTATATGGCGTGTACTCACGCCTCTAATCCAGTGTATGCTACTTTGAAGATACGGATCTACTTCTATGATTCCG-------TGACAAA------------------------TTAATAAATATTGAATTTTATTGAAGATGATTGGTTTACATATAC-----AACATGCTCTAATACATTCCATAATACATGATCAACTGCTCTAACTACATTATTAATACTGATAACTCCTAGATTATCTAAATACTTAAGCAC-------TTGGGTCTTAAAG---------------ACCCTTAAGAAACGA-------------CCAGTCGGAG-GCTGTGAGGTC-----------ATCCAGATTCGGGAAGGCTAGGAAAC-ATTTGTGTATCCCC------AACGCTTT----------CCTCAGGTTGTGATTGAACTGTATCTGGACAGTGATGATGTCTTGGTTC--------ATGAGGAATGGTCTGTTGTGGTGCTCTGTTATCTTGAAATAGAGGGGATTTTGAATCTCCC--------AGATA------------AATACGCCATTCTGTGCTTGAGCT--GCAGTGATGAGTTCCCCTGTGCG--TGAATCCATGGTTGTGGCAGGCTAATGCTATGAAG-------TATGAACACCCACACGGGAGATC-AACACGACGACGCC---TGGTCCCCTTCTTGGCTAGCCTGTGCTGCACTTTGA----TTGGAACCTGAGTAGAGTGGGCCTTCGAGGGTGATGAAGGT---------------------------CGCATTCTTTAATGC------CCAATTTTTAAGTGCAGAATTCTTCTCCTCATCCAAAAACTCTTTATAGCT-TGAGTTGGGTCCTGGATTGCAGAGGAAGATA-------------------------------------GCGGGAATTCCGCCTTTAA-TTTGAACTGGCTTTCCGTACTTTGTATTTGATTGCCAGTCCCTT-----------------------TGGGCCCCCATGAATTCTTTAAAGTG--------CTTTAGGTAGTGGGGATCGACGTCATCAATGACG-TTG----------------TACCAGGCCTC-GTTGCTGTAGAC---CTTTGGACTAAGGTCTAAATGACCACACAGATAATTGTGTGGACCCAGTGACCTGGCCCACATCGTCTTCCCCGT-------TCTACTATCACCCTCT-----------AAGACAATACTTTTAGGTCTCAATGG-CCGCGCAGCGGCATCCACCACGTTCTCAGCAGCCCAGACTTCAAGTTCTTCCGGAACTTGATCAAAAGAAGAAGAAGA------------------------------AAAAGGAGAAACATAAACCTCCACAGGAGGTGTAAAAAT-CCTATCTAAATTACA---TTTTAAATTATGATATTGAAAAATAAAATCTTTAGGGAGTTTTTCCCTAATTATTGCTAAAGCAGCGTCAGCCGAA---CCTGCATTTAGGGCCTCTGCTGCTGCAT-CATTAGCTGTCTGTT----GACCTCCTCTAGCAGATCTTCCATCGATCTGAAACTGACCCCAGTCGATGTAATCACCGTCCTTCTCGATGTAGGACTTGACATCTGAGCTGGA---CTTAGCT-----CCCTGGAAGTTTGGGTGGAATT--GGGTGGAGGTATTAGGGTGAGTGACATCGAAATGTCTAGGGGTTTCTGAACTTGGATTTACCTTTGAACTGGAT-GAGGGCATGGATAT---GCAGAGACCCATCTTGGTGTTTTTCTT-GTGATACTC--------TGATAAACAATTTATCAGAAGG--ACAGGTAATGTTTTTAAGTAGTTCGAGCATTTGCTCTTTCGGTATTGG----GCATTTTGGATAAGTAAGGAAGATATTTTTGGCATTTATACAGAAAGAGTTATTACGAGGCATATTGAA-TTGGGGACATTCA-AAAC---TCTGAGGAATGG-----------------------------------GGGACT------CGGGGGACGCATTTATATG--GTG-T-CCCCAAATGGCAATT-TGGTAA--------TTCA-GAAAG-AAATTTC-----AAAATCTCCA----C------GCT--CCAAAAA--GC-GGCCAT--CCGTA-TAATATT

>CLCuKoV-PK:Sak:05FN552006

-------ACCGGATGGCCGCGC--GATTTTT------TTGTGGGTCCTAC------CATTAATT------------CTTGTCGGCCAACCATATGACGC-GCTC-----------AAAGCTTAAATAATT-------TTCCCGCTTATTATAAGTACTTCGTTGCTAAGTATGCG---TTTGAAAA-----------ATGTGGGATCCACT------GTTAAATGAGTTCCCCGACACCGTTCACGGTTTTAGGTGTATGTTAGCAGTT--------AAATATTTGCA-------GTTAGTAGAGAAAACTTA-CTCTCCTGATAC---ATTGGGTTACGAT--TTGATAAGGGATTTAATCCTGGTAATAAGGGCTAGGAATTATGTCGAAGCGACCAGCAGATATAATCATTTCTACGCCCGCTTCGAAGGTACGCCGCCGTCTCAACTTCGACAGCCCATATGTGAGCCGTGCTGCTGCCCCCATT---GTCCGCG-----------------TCACCAAAGCAAA---AGCATGGGCGAACAGGCCCATGAACAGAAAGCCCAGGATGTAC-AGGATGTACAGAAGTCCAGATGTTCC-TAGAGGATGT----GAAGGTCCATGTAAGGTTCAGTCGTTTGAGTCCA-GACATGATATTCAGCATATA-GGTA-AAGTAATGTGTGTTAGTGATGTTACTCGTGGTACTGGGCTGACCCATAGAGTTGG---TAAGAGATTTTGTGTTA-AGTCT----GTTTATGTGTTGGGTAAGATCTGGATGGATGAGAACATTAAGACGAAGAATCACACGAATAGTGTGATGTTTTCCT-TGGTTAGAGATCGTAGACCTGTTGATAAA---CCTCAAGATTTTGGA-GAGGTATTTAATATGTTTGATAATGAGCCCAGTACGGCGACTGTGAAGAATGTTCATCGTGATAGGTATCAAGT-TCTGCGCA---AATGGTATGCA-----ACTGTCACCGGTGGACAATACGCTTCAAAGGAACAAGCTCTCGTGAAGAAA-TTTATTAGAGTTA-ATAATTATGT-TGTGTATAACCAG--------CAG-GAAGCTGGCAAGTATGAGAATCATTCTGAGAATGCTTTAATGTTGTATATGGCGTGTACTCACGCCTCTAACCCAGTGTATGCTACCTTGAAGATACGGATCTACTTCTATGATTCCG-------TGACAAA------------------------TTAATAAATATTGAATTTTATTGAAGATGATTGGTCTACAAATAC-----AACATGTTGTAATACATTCCATAATACATGATCAACTGCTCTAACTACATTATTAATACTGACAATTCCTAAGTTATTTAAATATTTAAGCAC-------TTGAGTCCTAAAG---------------ACCCTTAAGAAACGA-------------CCAGTCGGAG-GCTGTGAGGTC-----------ATCCAGATTC-GGAAAGCTATGAAAC-ATTTGTGTATCCCC------AACGCTTT----------CCTCAGGTTGTGATTGAACTGTATCTGGACGGTGATGATGTCTCTGTTC--------ATTAGGAATGGTCGATTGTGGTGCTCTGTTATCTTGAAATACAGGGGATTTTGAATCTCCC--------AGATA------------AACAGGCCATTCTCTGCTTGAGCT--GCAGTGATGAGTTCCCCTGTGCG--TGAATCCATGGTTGTGGCAGGCTAATGCTATGAAG-------TACGAACACCCACAAGGGAGATC-AACTCTCCGACGTC---TGGTCCCCTTCTTGGCTAGCCTGTGCTGCACTTTGA----TTGGAACCTGAGTAGAGTGGGCTCTCGAGGGTGATGAAGAT---------------------------TGCATTCTTTAAAGC------CCAATTTTTGAGTGCATAATTCTTCTCTTCATCCAAAAATTCTTTATAGCT-TGGATTGGGTCCTGGATTGCAGAGGAAGATA-------------------------------------GTGGGAATTCCGCCTTTAA-TTTGAACTGGCTTCCCGTATTTTGTATTTGATTGCCAGTCCTTT-----------------------TGGGCCCCCATGAACTCCTTAAAGTG--------CTTTAGGTAATGCGGGTCGACGTCATCAATGACG-TTA----------------AACCAGGCGTC-ATTACTGTATAC---CCTTGGGCTCAGATCTAGATGTCCACACAGATAATTATGTGGACCTAATGATCTGGCCCACATCGTCTTCCCCGT-------CCTACTGTCACCCTCA-----------ATCACTACACTTATTGGTCT-ATTGGCCCGCGCAGCGGCACTGACGACGTTCTCGGCAGCCCACACTTCAAGTTCTTCTGGAACTTGATCGAAAGAAGAAGAGGA------------------------------AAAAGGAGAAACATAAGGAGCTGGTGGCTCCTGAAAGAT-TCTGTCTAGATTTGC---ATTTAAATTATGAAATTGCAGTACAAAATCCTTAGGAGCTAGTTCCTTAATGACT-CTAAG--AGCCTCCGACTTACTTCCCGCGTTAAGTGCTGCGGCGTAAGCGT-CATTGGCTGTCTGTT----GCCCTCCTCTTGCTGACCTTCCGTCGATCTGAAACTCTCCCCACTCGAGAGTGTCCCCGTCCTTGTCGATGTAGGATTTGACGTCGGAGCTGGA---TTTAGCT-----CCCTGTATGTTCGGATGGAAAT--GTGCTGACCTGCTTGGGGAGACCAAGTCGAAGAATCG-CATATTCTGGCACTTGAATTTGCCTTCGAACTGGAT-GAGAACATGCAAGT---GAGGAGTCCCATCTTCGTGAAGCTCTC-TGCAGATTC--------TAATATATTTTTTTGAAGTTGG--GGTTTGTATATTTAATAATTGGGAAAGTGCTTCCTCTTTGGTGAGAGA----ACATTTGGGATAAGTGATGAAATAGTTTTTGGAATAAATACCGTTCCGCTTTGGAGGCATG---------TTGACTAAAATTG-GTCA---CCGATTGACCGC------TCTTG-----CAACTCTCCCCGGTATATCGGTGAT------CAA----------TATATA--GTGAT-CACCAAATGGCATAA-TGGTAA--------TAAA-AAAACTTTAATTT-----GAAATTCAAA----CCAAAAGGCT------AAA--GC-GGCCAT--CCGTT-TAATATT

>CLCuKoV-PK:Mul:08HM468427

-------ACCGGATGGCCGCGC--GATTTCT------TTGTGGGCCCTAC------CATTAACT------------CTTGTCGGCCAATCATATGACGC-GCTC-----------AAAGCTTAAATAATT-------CTCCCGCTTATTATAAGTACTTCGTTGCTAAGTATGCG---TTTGAAAA-----------ATGTGGGATCCACT------GTTAAATGAGTTCCCCGACACCGTTCACGGTTTTAGGTGTATGTTAGCAGTT--------AAATATTTGCA-------GTTAGTAGAGAAAACTTA-CTCTCCTGATAC---ATTGGGTTACGAT--TTGATAAGGGATTTAATCCTGGTAATAAGGGCTAGGAATTATGTCGAAGCGACCAGCAGATATAATCATTTCCACGCCCGCTTCGAAGGTACGCCGCCGTCTGAACTTCGACAGCCCATACGCGTGCCGTGCTGCTGCCCCCATT---GTCCGCG-----------------TCACCAAAGCAAA---AGCATGGGCGAACAGGCCCATGAACAGAAAGCCCAGGATGTAC-AGGATGTACAGAAGTCCAGATGTTCC-TAGAGGATGT----GAAGGTCCATGTAAGGTTCAGTCGTTTGAGTCCA-GACATGATATTCAGCATATA-GGTA-AAGTAATGTGTGTTAGTGATGTTACTCGTGGTACTGGGCTGACCCATAGAGTTGG---TAAGAGATTTTGTGTTA-AGTCT----GTTTATGTGTTGGGTAAGATCTGGATGGATGAGAACATTAAGACGAAGAATCACACGAATAGTGTGATGTTTTTCT-TGGTTAGAGATCGTAGACCTGTTGATAGA---CCTCAAGATTTTGGA-GAGGTATTTAATATGTTTGATAATGAGCCCAGTACGGCGACTGTGAAGAATGTTCATCGTGATAGGTATCAAGT-TCTGCGCA---AATGGTATGCA-----ACTGTCACCGGTGGACAATACGCTTCAAAGGAACAAGCTCTCGTGAAGAAA-TTTATTAGAGTTA-ATAATTATGT-TGTGTATAACCAG--------CAT-GAAGCTGGCAAGTATGAGAATCATTCTGAGAATGCTTTAATGTTGTACATGGCGTGTACTCACGCCTCTAACCCAGTGTATGCTACCTTGAAGATACGGATCTACTTCTATGATTCCG-------TGACAAA------------------------TTAATAGATATTGAATTTTATTGAAGATGATTGGTCTACAAATAC-----AACATGTTGTAATACATTCCATAATACATGATCAACTGCTCTAACTACATTATTAATACTGACAATTCCTAAGTTATTTAAATATTTAAGCCC-------TTGAGTCCTAAAG---------------ACTCTTAAGAAACGA-------------CCAGTCGGAG-GCTGTGAGGTC-----------ATCCAGATTC-GGAAAGCTATGAAAC-ATTTGTGTATCCCC------AACGCTTT----------CCTCAGGTTGTGATTGAACTGTATCTGGACGGTGATGATGTCTCTGTTC--------ATTAGGAATGCTCGATTGTGGTGCTCTGTTATCTTGAAATACAGGGGATTTTGAATCTCCC--------AGATA------------AACACGCCATTCTCTGCTTGAGCT--GCAGTGATGAGTTCCCCTGTGCG--TGAATCCATGGTTGTGGCAGGCTAATGCTATGAAG-------TACGAACACCCACAAGGGAGATC-AACTCTCCGACGTC---TGGTCCCCTTCTTGGCTAGCCTGTGCTGCACTTTGA----TTGGAACCTGAGTAGAGTGGGCTCTCGAGGGTGATGAAGAT---------------------------TGCATTCTTTAAAGC------CCAATTTTTGAGTGCAGAATTCTTCTCTTCATCCAAAAACTCTTTATAGCT-TGAATTGGGTCCTGGATTGCAGAGGAAGATA-------------------------------------GTGGGAATTCCGCCTTTAA-TTTGAACTGGCTTCCCGTATTTTGTATTTGATTGCCAGTCCTTT-----------------------TGGGCCCCCATGAACTCCTTAAAGTG--------CTTTAGGTAATGCGGGTCGACGTCATCAATGACG-TTA----------------AACCAGGCGTC-ATTACTGTATAC---CCTTGGGCTCAGATCTAGATGTCCACACAGATAATTATGTGGACCTAATGATCTGGCCCACATCGTCTTCCCCGT-------CCTACTGTCACCCTCA-----------ATCACTACACTTATTGGTCT-ATTGGCCCGCGCAGCGGCACTGACGACGTTCTCGGCAGCCCACACTTCAAGTTCTTCTGGAACTTGATCGAAAGAAGAAGAGGA------------------------------AAAAGGAGAAACATAAGGAGCTGGTGGCTCCTGAAAGAT-TCTGTCTAGATTTGC---ATTTAAATTATGAAATTGCAGTACAAAATCCTTAGGAGCTAGTTCCTTAATGACT-CTAAG--AGCCTCCGACTTACTTCCCGCGTTAAGTGCTGCGGCGTAAGCGT-CATTGGCTGTCTGTT----GCCCTCCTCTTGCTGACCTTCCGTCGATCTGAAATTGCCCCCAGTCGAGAATGTCCCCGTCCTTCTCGATGTAGGATTTGACGTCGGAGCTGGA---TTTAGCT-----CCCTGTATGTTCGGATGGAAAT--GTGCTGACCTGCTTGGGGAGACCAAGTCGAAGAATCG-CATATTCTGGCACTTGAATTTCCCTTCGAACTGGAT-GAGAACATGCAAGT---GAGGAGTCCCATCTTCGTGAAGCTCTC-TGCAGATTC--------TAATATATTTTTTTGAAGTTGG--GGTTTGTATATTTAATAATTGGGAAAGTGCTTCCTCTTTGGTGAGAGA----ACATTTGGGATAAGTTATGAAATAGTTTTTGGAATAAATACCGTTCCGCTTTGGAGGCATG---------TTGACTAAAATTG-ATCA---CCGATTGACCGC------TCTTG-----CAACTCTCCCCGGTATATCGGTGAT------CAA----------TATATA--GTGAT-CACCAAATGGCATAA-TGGTAA--------TAAA-AAAACTTTAATTT-----GAAATTCAAA----CCAAAAGGCT------AAA--GC-GGCCAT--CCGTT-TAATATT

>CLCuKoV-PK:Fai:96AJ002449

-------ACCGGATGGCCGCGC--GATTTTT------TTGTGGGCCCTAC------CATTAACT------------CTTGTCGGCCAATCATATGACGC-GCTC-----------AAAGCTTAAATAATT-------CTCCCGCTTATTATAAGTACTTCGTTGCTAAGTATGCG---TTTGAAAA-----------ATGTGGGATCCACT------GTTAAATGAGTTCCCCGACACCGTTCACGGTTTTAGGTGTATGTTAGCAGTT--------AAATATTTGCA-------GTTAGTAGAGAAAACTTA-CTCTCCTGATAC---ATTGGGTTACGAT--TTGATAAGGGATTTAATCCTGGTAATAAGGGCTAGGAATTATGTCGAAGCGACCAGCAGATATAATCATTTCCACGCCCGCTTCGAAGGTACGCCGCCGTCTCAACTTCGACAGCCCATATGTGAGCCGTGCTGCTGCCCCCATT---GTCCGCG-----------------TCACCAAAGCAAA---AGCATGGGCGAACAGGCCCATGAACAGAAAGCCCAGGATGTAC-AGGATGTACAGAAGTCCAGATGTTCC-TAGAGGATGT----GAAGGTCCATGTAAGGTTCAGTCGTTTGAGTCCA-GACATGATATTCAGCATATA-GGTA-AAGTAATGTGTGTTAGTGATGTTACTCGTGGTACTGGGCTGACCCATAGAGTTGG---TAAGAGATTTTGTGTTA-AGTCT----GTTTATGTGTTGGGTAAGATCTGGATGGATGAGAACATTAAGACGAAGAATCACACGAATAGTGTGATGTTTTTCT-TGGTTAGAGATCGTAGACCTGTTGATAAA---CCTCAAGATTTTGGA-GAGGTATTTAATATGTTTGATAATGAGCCCAGTACGGCGACTGTGAAGAATGTTCATCGTGATAGGTATCAAGT-TCTGCGCA---AATGGTATGCA-----ACTGTCACCGGTGGACAATACGCTTCAAAGGAACAAGCTCTCGTGAAGAAA-TTTATTAGAGTTA-ATAATTATGT-TGTGTATAACCAG--------CAG-GAAGCTGGCAAGTATGAGAATCATTCTGAGAATGCTTTAATGTTGTATATGGCGTGTACTCACGCCTCTAACCCAGTGTATGCTACCTTGAAGATACGGATCTACTTCTATGATTCCG-------TGACAAA------------------------TTAATAAATATTGAATTTTATTGAAGATGATTGGTCTACAAATAC-----AACATGTTGTAATACATTCCATAATACATGATCAACTGCTCTAACTACATTATTAATACTGACAATTCCTAAGTTATTTAAATATTTAAGCAC-------TTGAGTCCTAAAG---------------ACCCTTAAGAAACGA-------------CCAGTCGGAG-GCTGTGAGGTC-----------ATCCAGATTC-GGAAAGCTATGAAAC-ATTTGTGTATCCCC------AACGCTTT----------CCTCAGGTTGTGATTGAACTGTATCTGGACGGTGATGATGTCTCTGTTC--------ATTAGGAATGCTCGATTGTGGTGCTCTGTTATCTTGAAATACAGGGGATTTTGAACCTCCC--------AGATA------------AACACGCCATTCTCTGCTTGAGCT--GCAGTGATGAGTTCCCCTGTGCG--TGAATCCATGGTTGTGGCAGGCTAATGCTATGAAG-------TACGAACACCCACAAGGGAGATC-AACTCTCCGACGTC---TGGTCCCCTTCTTGGCTAGCCTGTGCTGCACTTTGA----TTGGAACCTGAGTAGAGTGGGCTCTCGAGGGTGATGAAGAT---------------------------TGCATTCTTTAAAGC------CCAATTTTTGAGTGCAGAATTCTTCTCTTCATCCAAAAACTCTTTATAGCT-TGAATTGGGTCCTGGATTGCAGAGGAAGATA-------------------------------------GTGGGAATTCCGCCTTTAA-TTTGAACTGGCTTCCCGTATTTTGTATTTGATTGCCAGTCCTTT-----------------------TGGGCCCCCATGAACTCCTTAAAGTG--------CTTTAGGTAATGCGGGTCGACGTCATCAATGACG-TTA----------------AACCAGGCGTC-ATTACTGTATAC---CCTTGGGCTCAGATCTAGATGTCCACACAGATAATTATGTGGACCTAATGATCTGGCCCACATCGTCTTCCCCGT-------CCTACTGTCACCCTCA-----------ATCACTACACTTATTGGTCT-ATTGGCCCGCGCAGCGGCACTGACGACGTTCTCGGCAGCCCATACTTCAAGTTCTTCTGGAACTTGATCGAAAGAAGAAGAGGA------------------------------AAAAGGAGAAACATAAGGAGCTGGTGGCTCCTGAAAGAT-TCTGTCTAGATTTGC---ATTTAAATTATGAAATTGCAGCACAAAATCCTTAGGAGCTAGTTCCTTAATGACT-CTAAG--AGCCTCCGACTTACTTCCCGCGTTAAGTGCTGCGGCGTAAGCGT-CATTGGCTGTCTGTT----GCCCTCCTCTTGCTGACCTTCCGTCGATCTGAAATTGCCCCCAGTCGAGAATGTCCCCGTCCTTCTCGATGTAGGATTTGACGTCGGAGCTGGA---TTTAGCT-----CCCTGTATGTTCGGATGGAAAT--GTGCTGACCTGCTTGGGGAGACCAAGTCGAAGAATCG-CATATTCTGGCACTTGAATTTCCCTTCGAACTGGAT-GAGAACATGCAAGT---GAGGAGTCCCATCTTCGTGAAGCTCTC-TGCAGATTC--------TAATATATTTTTTTGAAGTTGG--GGTTTGTATATTTAATAATTGGGAAAGTGCTTCCTCTTTGGTGAGAGA----ACATTTGGGATAAGTGATGAAATAGTTTTTGGAATAAATACCGTTCCGCTTTGGAGGCATG---------TTGACTAAAATTG-ATCA---CCGATTGACCGC------TCTTG-----CAACTCTCCCCGGTATATCGGTGAT------CAA----------TATATA--GTGAT-CACCAAATGGCATAA-TGGTAA--------TAAA-AAAACTTTAATTT-----GAAATTCAAA----CCAAAAGGCT------AAA--GC-GGCCAT--CCGTT-TAATATT

>CLCuKoV-PK:Fai:96AJ002448

-------ACCGGATGGCCGCGC--GATTTTT------TTGTGGGCCCTAC------CATTAACT------------CTTGTCGGCCAATCATATGACGC-GCTC-----------AAAGCTTAAATAATT-------CTCCCGCTTATTATAAGTACTTCGTTGCTAAGTATGCG---TTTGAAAA-----------ATGTGGGATCCACT------GTTAAATGAGTTCCCCGACACCGTTCACGGTTTTAGGTGTATGTTAGCAGTT--------AAATATTTGCA-------GTTAGTAGAGAAAACTTA-CTCTCCTGATAC---ATTGGGTTACGAT--TTGATAAGGGATTTAATCCTGGTAATAAGGGCTAGGAATTATGTCGAAGCGACCAGCAGATATAATCATTTCCACGCCCGCTTCGAAGGTACGCCGCCGTCTCAACTTCGACAGCCCATATGTGAGCCGTGCTGCTGCCCCCATT---GTCCGCG-----------------TCACCAAAGCAAA---AGCATGGGCGAACAGGCCCATGAACAGAAAGCCCAGGATGTAC-AGGATGTACAGAAGTCCAGATGTTCC-TAGAGGATGT----GAAGGTCCATGTAAGGTTCAGTCGTTTGAGTCCA-GACATGATATTCAGCATATA-GGTA-AAGTAATGTGTGTTAGTGATGTTACTCGTGGTACTGGGCTGACCCATAGAGTTGG---TAAGAGATTTTGTGTTA-AGTCT----GTTTATGTGTTGGGTAAGATCTGGATGGATGAGAACATTAAGACGAAGAATCACACGAATAGTGTGATGTTTTTCT-TGGTTAGAGATCGTAGACCTGTTGATAAA---CCTCAAGATTTTGGA-GAGGTATTTAATATGTTTGATAATGAGCCCAGTACGGCGACTGTGAAGAATGTTCATCGTGATAGGTATCAAGT-TCTGCGCA---AATGGTATGCA-----ACTGTCACCGGTGGACAATACGCTTCAAAGGAACAAGCTCTCGTGAAGAAA-TTTATTAGAGTTA-ATAATTATGT-TGTGTATAACCAG--------CAG-GAAGCTGGCAAGTATGAGAATCATTCTGAGAATGCTTTAATGTTGTATATGGCGTGTACTCACGCCTCTAACCCAGTGTATGCTACCTTGAAGATACGGATCTACTTCTATGATTCCG-------TGACAAA------------------------TTAATAAATATTGAATTTTATTGAAGATGATTGGTCTACAAATAC-----AACATGTTGTAATACATTCCATAATACATGATCAACTGCTCTAACTACATTATTAATACTGACAATTCCTAAGTTATTTAAATATTTAAGCAC-------TTGAGTCCTAAAG---------------ACCCTTAAGAAACGA-------------CCAGTCGGAG-GCTGTGAGGTC-----------ATCCAGATTC-GGAAAGCTATGAAAC-ATTTGTGTATCCCC------AACGCTTT----------CCTCAGGTTGAGATTGAACTGTATCTGGACGGTGATGATGTCTCTGTTC--------ATTAGGAATGCTCGATTGTGGTGCTCTGTTATCTTGAAATACAGGGGATTTTGAATCTCCC--------AGATA------------AACACGCCATTCTCTGCTTGAGCT--GCAGTGATGAGTTCCCCTGTGCG--TGAATCCATGGTTGTGGCAGGCTAATGCTATGAAG-------TACGAACACCCACAAGGGAGATC-AACTCTCCGACGTC---TGGTCCCCTTCTTGGCTAGCCTGTGCTGCGCTTTGA----TTGGAACCAGAGTAGAGTGGGCTCTCGAGGGTGATGAAGAT---------------------------TGCATTCTTTAAAGC------CCAATTTTTGAGTGCAGAATTCTTCTCTTCATCCAAAAACTCTTTATAGCT-TGAATTGGGTCCTGGATTGCAGAGGAAGATA-------------------------------------GTGGGAATTCCGCCTTTAA-TTTGAACTGGCTTCCCGTATTTTGTATTTGATTGCCAGTCCTTT-----------------------TGGGCCCCCATGAACTCCTTAAAGTG--------CTTTAGGTAATGCGGGTCGACGTCATCAATGACG-TTA----------------AACCAGGCGTC-ATTACTGTATAC---CCTTGGGCTCAGATTTAGATGTCCACACAGATAATTATGTGGACCTAATGATCTGGCCCACATCGTCTTCCCCGT-------CCTACTGTCACCCTCA-----------ATCACTACACTTATTGGTCT-ATTGGCCCGCGCAGCGGCACTGACGACGTTCTCGGCAGCCCACACTTCAAGTTCATCTGGAACTTGATCGAAAGAAGAAGAGGA------------------------------AAAAGGAGAAACATAAGGAGCTGGTGGCTCCTGAAAGAT-TCTGTCTAGATTTGC---ATTTAAATTATGAAATTGCAGTACAAAATCCTTAGGAGCTAGTTCCTTAATGACT-CTAAG--AGCCTCCGACTTACTTCCCGCGTTAAGTGCTGCGGCGTAAGCGT-CATTGGCTGTCTGTT----GCCCTCCTCTTGCTGACCTTCCGTCGATCTGAAATTGCCCCCAGTCGAGAATGTCCCCGTCCTTCTCGATGTAGGATTTGACGTCGGAGCTGGA---TTTAGCT-----CCCTGTATGTTCGGATGGAAAT--GTGCTGACCTGCTTGGGGAGACCAAGTCGAAGAATCG-CATATTCTGGCACTTGAGTTTCCCTTCGAACTGGAT-GAGAACATGCAAGT---GAGGAGTCCCATCTTCGTGAAGCTCTC-TGCAGATTC--------TAATATATTTTTTTGAAGTTGG--GGTTTGTATATTTAATAATTGGGAAAGTGCTTCCTCTTTGGTGAGAGA----ACATTTGGGATAAGTGATGAAATAGTTTTTGGAATAAATACCGTTCCGCTTTGGAGGCATG---------TTGACTAAAATTG-ATCA---CCGATTGACCGC------TCTTG-----CAACTCTCCCCGGTATATCGGTGAT------CAA----------TATATA--GTGAT-CACCAAATGGCATAA-TGGTAA--------TAAA-AAAACTTTAATTT-----GAAATTCAAA----CCAAAAGGCT------AAA--GC-GGCCAT--CCGTT-TAATATT

>CLCuKoV-PK:Fai:02AJ496286

-------ACCGGATGGCCGCGC--GATTTTT------TTGTGGGCCCTAC------CATTAACT------------CTTGTCGGCCAATCATATGACGC-GCTC-----------AAAGCTTAAATAATT-------CTCCCGCTTATTATAAGTACTTCGTTGCTAAGTATGCG---TTTGAAAA-----------ATGTGGGATCCACT------GTTAAATGAGTTCCCCGACACCGTTCACGGTTTTAGGTGTATGTTAGCAGTT--------AAATATTTGCA-------GTTAGTACAGAAAACTTA-CTCTCCTGATAC---ATTGGGTTACGAT--TTGATAAGGGATTTAATCCTGGTAATAAGGGCTAGGAATTATGTCGAAGCGACCAGCAGATATAATCATTTCCACGCCCGCTTCGAAGGTACGCCGCCGTCTCAACTTCGACAGCCCATATGTGAGCCGTGCTGCTGCCCCCATT---GTCCGCG-----------------TCACCAAAGCAAA---AGCATGGGCGAACAGGCCCATGAACAGAAAGCCCAGGATGTAC-AGGATGTACAGAAGTCCAGATGTTCC-TAGAGGATGT----GAAGGTCCATGTAAGGTTCAGTCGTTTGAGTCCA-GACATGATATTCAGCATATA-GGTA-AAGTAATGTGTGTTAGTGATGTTACTCGTGGTACTGGGCTGACCCATAGAGTTGG---TAAGAGATTTTGTGTTA-AGTCT----GTTTATGTGTTGGGTAAGATCTGGATGGATGAGAACATTAAGACGAAGAATCACACGAATAGTGTGATGTTTTTCT-TGGTTAGAGATCGTAGACCTGTTGATAAA---CCTCAAGATTTTGGA-GAGGTATTTAATATGTTTGATAATGAGCCCAGTACGGCGACTGTGAAGAATGTTCATCGTGATAGGTATCAAGT-TCTGCGCA---AATGGTATGCA-----ACTGTCACCGGTGGACAATACGCTTCAAAGGAACAAGCTCTCGTGAAGAAA-TTTATTAGAGTTA-ATAATTATGT-TGTGTATAACCAG--------CAG-GAAGCTGGCAAGTATGAGAATCATTCTGAGAATGCTTTAATGTTGTATATGGCGTGTACTCACGCCTCTAACCCAGTGTATGCTACCTTGAAGATACGGATCTACTTCTATGATTCCG-------TGACAAA------------------------TTAATAGATATTGAATTTTATTGAAGATGATTGGTCTACAAATAC-----AACATGTTGTAATACATTCCATAATACATGATCAACTGCTCTAACTACATTATTAATACTGACAATTCCTAAGTTATTTAAATATTTAAGCAC-------TTGAGTCCTAAAG---------------ACCCTTAAGAAACGA-------------CCAGTCGGAG-GCTGTGAGGTC-----------ATCCAGATTC-GGAAAGCTATGAAAC-ATTTGTGTATCCCC------AACGCTTT----------CCTCAGGTTGTGATTGAACTGTATCTGGACGGTGATGATGTCTCTGTTC--------ATTAGGAATGCTCGATTGTGGTGCTCTGTTATCTTGAAATACAGGGGATTTTGAATCTCCC--------AGATA------------AACACGCCATTCTCTGCTTGAGCT--GCAGTGATGAGTTCCCCTGTGCG--TGAATCCATGGTTGTGGCAGGCTAATGCTATGAAG-------TACGAACACCCACAAGGGAGATC-AACTCTCCGACGTC---TGGTCCCCTTCTTGGCTAGCCTGTGCTGCACTTTGA----TTGGAACCTGAGTAGAGTGGGCTCTCGAGGGTGATGAAGAT---------------------------TGCATTCTTTAAAGC------CCAATTTTTGAGTGCAGAATTCTTCTCTTCATCCAAAAACTCTTTATAGCT-TGAATTGGGTCCTGGATTGCAGAGGAAGATA-------------------------------------GTGGGAATTCCGCCTTTAA-TTTGAACTGGCTTCCCGTATTTTGTATTTGATTGCCAGTCCTTT-----------------------TGGGCCCCCATGAACTCCTTAAAGTG--------CTTTAGGTAATGCGGGTCGACGTCATCAATGACG-TTA----------------AACCAGGCGTC-ATTACTGTATAC---CCTTGGGCTCAGATCTAGATGTCCACACAGATAATTATGTGGACCTAATGATCTGGCCCACATCGTCTTCCCCGT-------CCTACTGTCACCCTCA-----------ATCACTACACTTATTGGTCT-ATTGGCCCGCGCAGCGGCACTGACGACGTTCTCGGCAGCCCACACTTCAAGTTCTTCTGGAACTTGATCGAAAGAAGAAGAGGA------------------------------AAAAGGAGAAACATAAGGAGCTGGTGGCTCCTGAAAGAT-TCTGTCTAGATTTGC---ATTTAAATTATGAAATTGCAGTACAAAATCCTTAGGAGCTAGTTCCTTAATGACT-CTAAG--AGCCTCCGACTTACTTCCCGCGTTAAGTGCTGCGGCGTAAGCGT-CATTGGCTGTCTGTT----GCCCTCCTCTTGCTGACCTTCCGTCGATCTGAAATTGCCCCCAGTCGAGAATGTCCCCGTCCTTCTCGATGTAGGATTTGACGTCGGAGCTGGA---TTTAGCT-----CCCTGTATGTTCGGATGGAAAT--GTGCTGACCTGCTTGGGGAGACCAAGTCGAAGAATCG-CATATTCTGGCACTTGAATTTCCCTTCGAACTGGAT-GAGAACATGCAAGT---GAGGAGTCCCATCTTCGTGAAGCTCTC-TGCAGATTC--------TAATATATTTTTTTGAAGTTGG--GGTTTGTATATTTAATAATTGGGAAAGTGCTTCCTCTTTGGTGAGAGA----ACATTTGGGATAAGTTATGAAATAGTTTTTGGAATAAATACCGTTCCGCTTTGGAGGCATG---------TTGACTAAAATTGAATCA---CCGATTGACCGT------TCTTG----CAAACTCTCCCCGGTATATCGGTGAT------CAA----------TATATA--GTGAT-CACCAAATGGCATAA-TGGTAA--------TAAA-AAAACTTTAATTT-----GAAATTCAAA----CCAAAAGGCT------AAA--GC-GGCCAT--CCGTT-TAATATT

>CLCuKoV-IN:SriBt:10HQ257374

-------ACCGGATGGCCGCGC--GATTTTT------TTGTGGGCCCTAC------CATTAACT------------CTTGTCGGCCAATCATATGACGC-GCTC-----------AAAGCTTAAATAATT-------CTCCCGCGTATTATAAGTACTTCGTTGCTAAGTATGCG---TTTGAAAA-----------ATGTG--ATCCACT------GTTAAATGAGTTCCCCGACACCGTTCACGGTTTTAGGTGTATGTTAGCAGTT--------AAATATTTGCA-------GTTAGTAGAGAAAACTTA-CTCTCCTGATAC---ATTGGGTTACGAT--TTGATAAGGGATTTAATCCTGGTAATAAGGGCTAGGAATTATGTCGAAGCGACCAGCAGATATAATCATTTCCACGCCCGCTTCGAAGGTACGCCGCCGTCTCAACTTCGACAGCCCATATGTGAGCCGTGCTGCTGCCCCCATT---GTCCGCG-----------------TCACCAAAGCAAA---ATCATGGGCGAACAGGCCCATGAACAGAAAGCCCAGGATGTAC-AGGATGTACAGAAGTCCAGATGTTCC-TAGAGGATGT----GAAGGTCCATGTAAGGTTCAGTCGTTTGAGTCCA-GACATGATATTCAGCATATA-GGTA-AAGTAATGTGTGTTAGTGATGTTACTCGTGGTACTGGGCTGACCCATAGAGTTGG---TAAAAGATTTTGTGTTA-AGTCT----GTTTATGTGTTGGGTAAGATCTGGATGGATGAGAACATTAAGACGAAGAATCACACGAATAGTGTGATGTTTTTCT-TGGTTAGAGATCGTAGACCTGTTGATAAA---CCTCAAGATTTTGGA-GAGGTATTTAATATGTTTGATAATGAGCCCAGTACGGCGACTGTGAAGAATGTTCATCGTGATAGGTATCAAGT-TCTGCGCA---AATGGTATGCA-----ACTGTCACCGGTGGACAATACGCTTCAAAGGAACAAGCTCTCGTGAAGAAA-TTTATTAGAGTTA-ATAATTATGT-TGTGTATAACCAG--------CAG-GAAGCTGGCAAGTATGAGAATCATTCTGAGAATGCTTTAATGTTGTATATGGCGTGTACTCACGCCTCTAACCCAGTGTATGCTACCTTGAAGATACGGATCTACTTCTATGATTCCG-------TGACAAA------------------------TTAATAAATATTGAATTTTATTGAAGATGATTGGTCTACAAATAC-----AACATGTTGTAATACATTCCATAATACATGATCAACTGCTCTAACTACATTATTAATACTGACAATTCCTAAGTTATTTAAATATTTAAGCAC-------TTGAGTCCTAAAT---------------ACCCTTAAGAAACGA-------------CCAGTCGGAG-GCTGTGAGGTC-----------ATCCAGATTC-GGAAAGCTATGAAAC-ATTTGTGTATCCCC------AACGCTTT----------CCTCAGGTTGTGATTGAACTGGATCCTGATCGTGAGTATATCCATATTC--------GTCGTGAATGGACGGTTGACGTGGCTGATGATCTTGAAATAAAGGGGATGTGGAACCTCCC--------AGATA------------TATGCGACATTCCCTGCTAGAGCT--GAAGTGATGGGTTCCCTTGTGCG--TGAATCCATGGTTGTGGCAGTTGATTGACAGATTA-------TAATAACACGCGCATTCAAGATC-TATTCTCCTCCTCC---TGTTGCGTCTCTTCGCTTCCCTGTGCTGTACTTTGA----TTGGAAGCTGAGTACAGTGGTCCTTCGAGAGTGATGAAGAT---------------------------TGCATTCTTTAAAGC------CCAATTTTTTAGTGCAGAATTCTTCTCTTCATCCAAAAACTCTTTATAGCT-TGAATTGGGTCCTTGATTGCAGAGGAAGATA-------------------------------------GTGGGAATTCCGCCTTTAA-TTTGAACTGGCTTCCCGTATTTTGTATTTGATTGCCAGTCCTTT-----------------------TGGGCCCCCATGAACTCCTTAAAGTG--------CTTTAGGTAATGCGGGTCGACGTCATCAATGACG-TTA----------------AACCAGGCGTC-ATTACTGTATAC---CCTTGGGCTCAGATCTAGATGTCCACACAGATAATTATGTGGACCTAATGATCTGGCCCACATCGTCTTCCCCGT-------CCTACTGTCACCCTCA-----------ATCACTAAACTTATTGGTCT-ATTGGGCCGCGCAGCGGCACTGACGACGTTCTCGGCAGCCCACACTTCAAGTTCTTCTGGAACTTGATCGAAAGAAGAAGAGGA------------------------------AAAAGGAGAAACATAAGGAGCTGGTGGCTCCTGAAAGAT-TCTGTCTAGATTTGC---ATTTAAATTATGAAATTGCAGTACAAAATCCTTAGGAGCTAGTTCCTTAATGACT-CTAAG--AGCCTCCGACTTATTTCCCGCGTTAAGTGCTGCGGCGTAAGCGT-CATTGGCTGTCTGTT----GCCCTCCTCTTGCTGACCTTCCGTCGATCTGAAATTGCCCCCAGTCGAGAATGTCCCCGTCCTTCTCGATGTAGGATTTGACGTCGGAGCTGGA---TTTAGCT-----CCCTGTATGTTCGGATGGAAAT--GTGCTGACCTGCTTGGGGAGACCAAGTCGAAGAATCG-CATATTCTGGCACTTGAATTTCCCTTCGAACTGGAT-GAGAACATGCAAGT---GAGGAGTCCCATCTTCGTGAAGCTCTC-TGCAGATTC--------TAATATATTTTTTTGAAGTTGG--GGTTTGTATATTTAATAATTGGGAAAGTGCTTCCTCTTTGGTGAGAGA----ACATTTGGGATAAGTGATGAAATAGTTTTTGGAATAAAAACCGTTCCGCTTTGGAGGCATG---------TTGACTAAAATTG-ATCA---CCGATTGACCGC------TCTTG-----CAACTCTCCCCGGTATATCGGTGAT------CAA----------TATATA--GTGAT-CACCAAATGGCATAA-TGGTAA--------TAAA-AAAACTTTAATTT-----GAAATTCAAA----CCAAAAGGCT------AAA--GC-GGCCAT--CCGTT-TAATATT

>CLCuKoV-In:Dab:04AY456683

-------ACCGGATGGCCGCGC--GATTCTT------TTGTGGGTCCCGC------CACTAACT------------CTTGTCTGCCAATCACATGGCGCGGCTCAAAGCTTAAATAAAGCTTAAATAATT-------CTCCCGCTTATTATAAGTACTTCGTTGCTAAGTATGCG---TTTGAAAA-----------ATGTGGGATCCACT------GTTAAATGAGTTCCCCGACACCGTACTCGGATTCAGGTGTATGCTTGCTATA--------AGATACCTGCA-------GTTGGTAGAAAATGCGTA-TTCCCCCGATTC---GTTGGGATACGAC--CTAATACGTGATTTAATTTCTGTCGTCAGGGCCAAAAGCTATGTCGAAGCGACCAGCAGATATAATCATTTCCACGCCCGCTTCGAAGGTACGCCGCCGTCTCAACTTCGACAGCCCATATGTGAGCCGTGCTGCTGCCCCCATT---GTCCGCG-----------------TCACCAAAGCAAA---AGCATGGGCGAACAGGCCCATGAACAGAAAGCCCAGGATGTAC-AGGATGTACAGAAGTCCAGATGTTCC-TAGAGGATGT----GAAGGTCCATGTAAGGTTCAGTCGTTTGAGTCCA-GACATAATATTCAGCATATA-GGTA-AAGTAATGTGTGTTAGTGATGTTACTCGTGGTACTGGGCTGACCCATAGAGTTGG---TAAGAGATTTTGTGTTA-AGTCT----GTTTATGTGTTGGGTAAGATCTGGATGGATGAGAACATTAAGACGAAGAATCACACGAATAGTGTGATGTTTTTCT-TGGTTAGAGATCGTAGACCTGTTGATAAA---CCTCAAGATTTTGGA-GAGGTATTTAATATGTTTGATAATGAGCCCAGTACGGCGACTGTGAAGAATGTTCATCGTGATAGGTATCAAGT-TCTGCGCA---AATGGTATGCA-----ACTGTCACCGGTGGACAATACGCTTCAAAGGAACAAGCTCTCGTGAAGAAA-TTTATTAGAGTTA-ATAATTATGT-TGTGTATAACCAG--------CAG-GAAGCTGGCAAGTATGAGAATCATTCTGAGAATGCTTTAATGTTGTATATGGCGTGTACTCACGCCTCTAACCCAGTGTATGCTACCTTGAAGATACGGATCTACTTCTATGATTCCG-------TGACAAA------------------------TTAATAAATATTGAATTTTATTGAAGATGATTGGTCTACAAATAC-----AACATGTTGTAATACTTTCCATAATACATGATCAACTGCTCTAACTACATTATTAATACTGACAATTCCTAAGTTATTTAAATATTTAAGCAC-------TTGAGTCCTAAAG---------------ACCCTTAAGAAACGA-------------CCAGTCGGAG-GCTGTGAGGTC-----------ATCCAGATTC-GGAAAGCTATGAAAC-ATTTGTGTATCCCC------AACGCTTT----------CCTCAGGTTGTGATTGAACCGTATCTGCACGGTGATGATGTCTCTGTTC--------ATTAGGAATGGTCGATTGTGGTGCTCTGTTATCTTGAAATACAGGGGATTTTGAATCTCCC--------AGATA------------AACACGCCATTCTCTGCTTGAGCT--GCAGTGATGGGTTCCCCTGTGCG--TGAATCCATGGTTGTGGCAGGCTAATGCTATGAAG-------TACGAACACCCACAAGGGAGATC-AACTCTCCGACGTC---TGGTCCCCTTCTTGGCTAGCCTGTGCTGCACTTTGA----TTGGAACCTGAGTAGAGTGGGCTCTCGAGGGTGATGAAGAT---------------------------TGCATTCTTTAAAGC------CCAATTTTTGAGTGCAGAATTCTTCTCTTCATCCAAAAACTCTTTATAGCT-TGAATTGGGTCCTGGATTGCAGAGGAAGATA-------------------------------------GTGGGAATTCCGCCTTTAA-TTTGAACTGGCTTCCCGTATTTTGTATTTGATTGCCAGTCCTTT-----------------------TGGGCCCCCATGAACTCCTTAAAGTG--------CTTTAGGTAATGCGGGTCGACGTCATCAATGACG-TTA----------------AACCAGGCGTC-ATTGCTGTATAC---CCTTGGGCTCAGATCTAGATGTCCACACAGATAATTATGTGGACCTAATGATCTGGCCCACATCGTCTTCCCCGT-------CCTACTGTCACCCTCA-----------ATCACTACACTTATTGGTCT-ATTGGCCCGCGCAGCGGCACTGACGACGTTCTCGGCAGCCCACACTTCAAGTTCTTCTGGAACTTGATCGAAAGAAGAAGAGGA------------------------------AAAAGGAGAAACATAAGGAGCTGGTGGCTCCTGAAAGAT-TTTGTCTAGATTTGC---ATTTAAATTATGAAATTGCAGTACAAAATCCTTAGGAGCTAGTTCCTTAATGACT-CTAAG--AGCCTCCGACTTACTTCCCGCGTTAAGTGCTGCGGCATAAGCGT-CATTGGCTGTCTGTT----GCCCTCCTCTTGCTGACCTTCCGTCGATCTGAAATTGCCCCCAGTCGAGAATGTCCCCGTCCTTCTCGATGTAGGATTTGACGTCGGAGCTGGA---TTTAGCT-----CCCTGTATGTTCGGATGGAAAT--GTGCTGACCTGCTTGGGGAGACCAAGTCGAAGAATCT-CTGATTCTGGCACTGGTACTTCCCTTCGAACTGGAT-GAGCACGTGAATAT---GAGGTTCCCCATTTTCGTGAAGCTCTC-TGCAGATTT--------TGATGAATTTTTTATTTGTTGG--GGTTTGTAGGTTTTGAAGTTGGGAAAGTGCTTCCTCTTTTGTGAGAGA----GCATTTTGGATAAGTGAGGAAATAATTTTTGGAATTAATAANAAAACGTTTNGGATGCATG---------TTGACCAAAAAAA-AGAC---CCGATTGACCAG------CTCTT----ACAACTCTCCCCTGTATATTGGGTCT------CAA----------TATATA--GTGAG-ACCCCAATGGCATTG-CCGTAA--------TTTT-GGAAC-GAAAATC-----AAAATCCTCA----C------CCT--CCAAAAA--GC-GGACAT--CCGCA-TAATATT

>PedLCV-PKMulPed04AM712436

AATATTAGACGGATGGCCGCTTTAGCCTTTT---CGTTTGAATTT---------------------------------------CAAATTAGAGTTCTTTTATT-----------ACAATTATGCCATTT-----GGTACTCAC----TATAT--ATTGAGTACCGATATACAGG---TGATAAACATCCAGAACCCAATTGGTACCCATTTGACCAAGTCAATGGCTCCTCCTAAGC-------GTTTTCAAATATATGCCAGAAATTATTTCCTCACTTACCCACACTGCTCTCTTACTAAAGAAGAAGCACTTTCCCAAATACAAGCCCTAGATACACCCACCAATAAAAAATT------CATCAAGATCTGCAGGGAACTCCA-CGAAGATGGGAGCCCTCATCTGCACGTGCTCATCCAGTTCGAAGGGAAATACAGGTGCCAAAATCAGCGATTCTT--------CGACCTGGTCTCCCCAACCAGGTCAGCACATTTCCATCCGAACATTCAGGGAGCTAAATCAAGCTCCGACGTCAAGTCCTATCTGGAGAAGGACGGAGACACCCTCGAATGGGGAGAGTTTCAGAT---CGATGGAAGATCTGCTAGAGGG---GGGCAACAATCAGCCAACGACGCTTACGCCGCAGCGCTTAACGCAGGCAGTA--AGTCA-----------GAGGCTCTTAGAGTCATTAAG----------GAGTTAGCTCCAAAAGATTATGTATTACAATTTCATAATTTAAATGCTAATTTAGAT-----------AGGATTTTTACACC-----TCCTGTGGA--GGTTTATGTATCTCC-TTTTTCTTCTTCTT----CTTTTGATCAAGTTCCGGAAGAACTTGAA---------------GTCTG----------------GGCTGCTGAAAA----------CGTGATGGATGCC-----GCTGCGCGGCCATTGAGACCTAAAAGTATTGTCATAGA-GGGCGATAGTCGTACGGGGAAGACGAT---GTGGGCTAG-GTCACTGGGTCCACATAATTATTTGTGTGGTCACTTAGACCTTAGTCCA-AAGGTCTACAGTAATGAG-----------GCCTGGTACAACGTCATTGATGACGTCGATCCGCATT---------ATCTAAAGCACTTCAAAGA---------ATTCATGGGGGCCCAAAGGGACTGGCAAAGCA---------------------ACACAAAATACGGAAAGCCAGT--------TCAAATTAAAGGCGG-----AATTCCCACTATCTTCCTCTGCAATCCAGGACCCAAT--TCAAGCTATA-----------AAGAGTTCTTGGACGAAGAGAAGAATTCTGCACTCAAAAATTGGGCTTTAAAGAATGCGATCTTCGTCACCCTCGAAGGCCCACTCTACCCAGGTTCCAATCAAAGTGCAGCACAGGC---------TAGCCAAGA----AGGGGACCAGGCGTCGACGTGTTGATCTCCCTTGTGGATGTTCTTATTTCATAGCACTAGCCTGTCACAGTCATGGATTCACGCACCGGGGAACTCATCACTGCAGCTCAAGCAGAGAATGGCGTGTT------------TATCT--------GGGAGATTCAAAATCCCCTCTATTTCAAGATAACAGAGCACCACAACAGGCCATTCAC--CATGAACCAAGACATCATAACCGTCCAGATACAGTTCAATCACAACCTGAGGAAAGCGTTGGGGATACACAAATGTTTCCTAGCCTTCAGAATCTGGATG-ACCTCACAGCCTCCGACTGGTCGTTTCTTAAG------------GGTCTTTAAGACCCAAGTACTTAAGTATTTAGATAATTTAGGAGTTATCAGTATTAACAATGTAATTAGAGCAGTTGATCATGTATTATGGAATGTATTACATCATGTTGTAAATATAGAACAATCAT----ATTCAATAAAATTTAATATTTATTAATTCGATACTGAATCATAGAAGTAGATCCGTATCTTCAATGTAGCATACACAGGGTTAGAGGCGTGCGTACACGCCATATACAA-CATCAACGCATTCTCAGTATGATTCTCATACTTCCCAGCCTCTTGCTGGTTGTACACAACATAATTATTAACCCTAATAAACTTCTTCACGAGAGCCTGCTCCTTAG---ATGCATATTGAC--CGCCTGTCACAGTTGCGTACCACTTCCTTAATACCTGGTATCTATCACGATGAACATTCTTCACAGTC--GCCGTGCTGGGCTCATTATCAAACATGT-----TAAAAACCTCGCCAAAATCTTGAGGCTTGTCAACGGGCCTACGATCTCTAACAAGAAAAAACATAACACTATTCGTATGATTCTTAGTTT------------------TAATGTTCTCATCCATCCAGATCTTA------CCCAGAACATAAACGGACTTAACACAAAATCTCTTGCCTACCCTGTGGGTAAGCCCAATACCACGAGTAACATCA----CTAA--------CACACATGACTTTACCAATGTGCTGGATATCATGTCTGGACTCAAATGACTGGACCTTACATGGG-----CCTTCACATCCTCTAGG--AACATCTGGACTTCTG--------------TACATCCTGTACAT-CCTGGGCTTTCTGTTCATGGGCCTGTTCGCCCATGCCCT-TGCCTTTGTGA-------CGCGGACAATGGGGGCAGCAGCACGGCTCGCATATGGGCTGTC----GAAGTTGA---GACGGCGGCGTACCTTCGAGGCGGGCGTGGAAAT--GATTATATCTGCTGGTCGCTTCGACAT-----AATTCCTAGCCCTTATCACTGAAATTAAATCCCGAATTAAATCGAATCCCAGAGTATCTGGGGAATACGTACTTTC----TACTAAC-TGCAAATATTTAACTGCTAACATACACCTAAAACCGTGAACGGTT--------------TCGGGAAA-------CTCGTCAATAACGGATCCCACATATG---TGAATGTGAAACACAACTTGCGCACTAAGTTTA-------TAGGGGGGACC--ATGAAA--------TAATTA------------------------------------------------GGCTATGAGGAG-------CCA----------TTCTCA--TTGGT---CCACATGTCATTGTCAGTTAGTGCTC--TGTG-GGGGCCATAA------------------------------------AAAAAGCGC-GGCCAT--CCGGT--------

>BYVMV-IN:Mad:00aazAF241479

TAATATTACCGGATGGCCGCGCGATTTTTTAAG----TGGTGGGT-CCAG--AACGCAC-----------------------GACGATGCAGA--------CTC-----------AAAGCTTAGATAACG--------CTCCTTCGGCTATAAGTACGTGCGCACTAAGTTTCAA---TTCAAAAA-----------ATGTGGGATCCACT------ATTAAACGAATTTCCGGATACGGTTCACGGGTTTCGTTGTATGCTATCTCTA--------AAATATTTGCA-------ACTTTTGTCGCAGGATTA-TTCTCCAGATAC---GCTTGGGTACGAG--TTAATACGGGATTTAATTTGTATTTTACGTTCCCGTAATTATGTCGAAGCGAGCTGCCGATATCGTCATTTCTACGCCCGCGTCGAAAGTACGCCGGCGTCTGAACTTCGGCAGCCCATACACCAGCCGTGCTGCTGCCCCCATT---GTCCGCG-----------------TCACAAAACAACA---GGCATGGACAAACAGGCCTATGAACAGGAAACCCAGAATGTAC-CGGATGTACAGAAGTCCGGATGTTCC-AAGGGGATGT----GAGGGTCCCTGTAAGGTACAGTCGTTTGAGTCTC-GACACGATGTCGTTCATATT-GGTA-AGGTAATGTGTATTTCGGATGTTACGCGTGGAGTCGGTTTGACCCATCGTATAGG---TAAGCGTTTTTGTGTCA-AGTCA----GTTTATGTTTTAGGTAAGATATGGATGGACGAGAACATCAAGACGAAGAACCATACGAATTCGGTGATGTTTTTCC-TTGTTCGTGATCGACGACCGGTAGATAAA---CCACAAGATTTTGGT-GAAGTATTTAATATGTTTGATAATGAGCCCAGTACGGCGACCGTGAAGAACATGCATAGGGATCGTTACCAGGT-GTTGAGGA---AATGGCATGCA-----ACCGTTACTGGTGGTCAATATGCAGCGAGGGAACAGGCGTTGGTTAAGAAA-TTTGTCAGGGTTA-ACAATTATGT-TGTTTACAACCAG--------CAG-GAGGCAGGAAAATACGAGAATCACACCGAGAATGCATTGATGCTTTATATGGCTTGTACCCATGCTAGTAACCCAGTGTATGCTACGCTTAAGATTCGGATTTATTTTTATGACTCTG-------TAACGAATTGAA-------------------TTAATAAAGTTTGAATTTTATATCTGAATATTGGTCTACATACAT-----TGTCTGATTAACTACATTGTACAATACATGTTCGACGGCTTTAATAACTAAATTAAGTGAGATTACACCTAGATTATTGAGATATTTGAGGAC-------TTGGGTTTTGAAT---------------ACCCTTAAGAAAAGA-------------CCAGTCGGAG-GGTGTAAGGTC-----------GTCCAGATTC-GGAAGGTTAGAAAAC-ACTTGTGTATTCCC------AGAGCTTT----------CCGTAGGTTGTAGTTGAAATGGATCCTGAGTGTTATTATGTCCATGTTC--------GTCGTGAATGGACGGTTGTCGTGGTTGAGGATCTTGAAATAGAGGGGATTTGGAACCTTCC--------AGATA------------TAGACGCCATTCTTTGCTTGAGCT--GCAGTGATGCGTTCCCCTGTGCG--AGAATCCATGGTTGTGGCAGTTGATGCTAAGATAA-------TAACTGCATCCGCATTCAAGGTC-CACTCGTCTCCTCC---TGTGCACTCGCTTCGCTTCCTTGTGTTGAACTTTGA----TTGGTACCCGAGTACAACGGTTGGGTGAGAAAGACGAATGC---------------------------TGCATTTTTTAAAGC------CCACGCTTTCAAAGCTGAGTTCTTTTCCTCGTCTAAAAACTCTTTATAGCT-TGCGTTGGGGCCTGGATTGCAGAGGAAGATT-------------------------------------GTTGGAATGCCTCCTTTAA-TTTGAACTGGCTTCCCGTACTTTGTGTTTGATTGCCAGTCCCTT-----------------------TGGGCCCCCATGAATTCTTTAAAGTG--------TTTCAGATAATGCGGGTCAACATCATCTATAATG-TTG----------------AACCACGCATC-GTTTGAATACAC---TTTAGGGCTTAGATCCAAGTGCCCACATAAATAATTATGTGGCCCCAAGGAACGGGCCCATTGTGTTTTCCCAGT-------TCTAGACTCCCCCTCT-----------AGAACTAAACTCAAAGGTCTCTGAGG-CCGCGCAGCGGCGTCCATGACGTTCTCCGACGCCCACTCTTCAAGTTCTTCTGGAACTTGATCAAAAGAAGAAGATAA------------------------------AAATGGACAAACATAAACCTCCTGAGGAGGAGTAAAAAT-CCTATCTAAATTTGA---ATTTAAATTATGATATTGTAAGACATAATCTTTGGGGGCTAATTCTTTAAGAACT-CTAAG--AGCCTCTGCCTTACTTCCTGTGTTAAGTGCTGCCGCGTAAGCGT-CATTGGCTGATTGTT----GTCCTCCCCTTGCAGATCTGCCGTCGATCTGGAATTCCCCCCATTCAAGGGTATCTCCGTCCTTGTCGATATAGGACTTGACGTCGGAGCTGGA---TTTAGCT-----CCCTGAATGTTTGGATGGAAAT--GTGCTGACCTGTTTGTGGCGACCAAGTCGAAGAATCT-CTGATTCTGGCACTTGTATTTTCCTTCGAACTGGAT-GAGCACGTGAAGAT---GAGGTTCCCCATTTTCATGAAGCTCAC-TGCAGATCT--------TTATGTATTTTTTATTTGTTGG--GGTTTGTATATTTTGTAGTTGGGATAATGCCTCTTCTTTATTAAGAGA----GCATTGTGGATAAGTAAGGAAATAATTTTTGGAATTGATGACAAAACGCCTTGGAGGCATG---------TTGACTATTTTTG-AGAC---CCGATTGACCGC------TCTTA-----CAACTCTCCCCAGTATATCGGGTCC------CTA----------TATATA--GTGAG-ACCCAAATGGCATAA-TTGTAA--------TAAA-ACAACTTTAATTT-----GAAATTCAAA----CGAAAAGGCT------AAA--GC-GGCCAT--CCGTA--------

>BYVMV-IN:Kar:06aazGU112023

-------ACCGGATGGCCGCGCGATTTTTTTAAG---TGGTGGGT-CCAG--AACGCAC-----------------------GACGATGCAGA--------CTC-----------AAAGCTTAGATAACG--------CTCCTTCGGCTATAAGTACGTGCGCACTAAGTTTCAA---TTCAAAAA-----------ATGTGGGATCCACT------ATTAAACGAGTTTCCGGATACGGTTCACGGGTTTCGTTGTATGCTATCTGTA--------AAATATTTGCA-------ACTTTTGTCGCAGGATTA-TTCTCCAGATAC---GCTTGGGTACGAT--TTAATACGGGATTTAATTTGTATTTTACGCTCCCGTAATTATGTCGAAGCGAGCTGCCGATATCGTCATTTCTACGCCCGCGTCGAAAGTACGCCGGCGTCTGAACTTCGGCAGCCCATACACCAGCCGTGCTGCTGCCCCCATT---GTCCGCG-----------------TCACAAAACAACA---GGCATGGACAAACAGGCCTATGAACAGGAAACCCAGAATGTAC-CGGATGTACAGAAGTCCGGATGTTCC-AAGGGGATGT----GAGGGTCCCTGTAAGGTACAGTCGTTTGAATCTC-GACACGATGTCGTTCATATT-GGTA-AGGTAATGTGTATTTCGGATGTTACGCGTGGAGTCGGTTTGACCCATCGTATAGG---TAAGCGTTTTTGTGTCA-AGTCA----GTTTATGTTTTAGGTAAGATATGGATGGACGAGAACATTAAGACCAAGAACCATACGAATTCGGTGATGCCTTTCC-TCGTTCGTGATCGACGACCGACAGATAAA---CCACAAGATTTTGGT-GAAGTGTTTAATATGTTTGATAACCAGCCCGATATGGCCACCGTTAAGAACATGCATAGGGATCGGTACCAGGT-GTTGAGGA---AATGGCATGCA-----ACCGTTACTGGTGGTCAATATGCATCTAAGGAACAGGCGTTGGTTAAGAAG-TTTATCAGGGTTA-ACAATTATGT-TGTTTACAACCAG--------CAG-GAGGCAGGAAAATACGAGAATCACACCGAGAATGCATTGATGCTTTATATGGCTTGTACCCATGCTAGTAACCCAGTTTATGCTACTCTTAAGATTCGGATATATTTTTATGACTCTG-------TAACGAATTGAAA------------------TTAATAAAGTTTTAATTTTATATCTGAATATTGGTCTACATACAT-----TGTTTGATTAATTACATTGTACAATACATGTTCAACGGCTTTAATAACTAAATTAAGTGAGATTACACCTAGATTGTTGAGATACTTGAATAC-------TTGGGTTTTGAAT---------------ACCCTTAAGAAAAGA-------------CCAGTCTGAG-GGTGTAAGGTC-----------GTCCAGATTC-GGAAGGTTAGAAAAC-ACTTGTGCACTCCC------AGAGCTTT----------CCGTAGGTTGTAGTTGAATTGGATCCTCATTGTTATGATGTCCATGTTC--------GTCGTGAATGGACGGTTGTCGTGGCTGAGGATTTTGAAATAAAGGGGATTTGGAACCTTCC--------AGATA------------TAGACGCCATTCGTTGAATGAGCT--GCAGTGATGCGTTCCCCTTTGCG--AGAATCCATGGTTGTGGCAGTTGATACTTAGGTAA-------TAGCTGCATCCACACTCAAGGTC-CACTCGTTTCCTCA---TGTGCGCTCTCTTCGCTTCCCTGTGTTGAACTTTGA----TTGGTACCGGAGTAGAGCGGTTGGGTGAGAGAGACGAATGT---------------------------TGCATTCTTTAAAGC------CCAGGATTTTAATGCTGAGTTCTTATCCTCGTCTAAGAACTCTTTATAGCT-TGCATTGGGCCCTGGATTGCAGAGGAAGATT-------------------------------------GTTGGTATGCCGCCTTTAA-TTTGAACTGGCTTCCCGTATTTTGTGTTTGATTGCCAGTCCCTT-----------------------TGGGCCCCCATGAATTCTTTAAAGTG--------TTTTAGGAAGTGTGGATCGACGTCATCAATGACG-TTA----------------TACCAAGCGTC-GTTACTGTACAC---CTTTGGGCTAAGGTCCAGATTCCCGCATAAATAGTTATGTGGGCTTAAAGACCTAGCCCACATTGTTTTTCCAGT-------ACGACTATCTTCCTCA-----------ATTACTATACTTTGAGGTCTCAGGGG-CCGCGCAGCGGCATCGACAACGTTCTCGCACGCCCACTCTTCAAGTTCTTCTGGAACTTGATCAAATGAAGAAGAAGA------------------------------AAAAGGAGAAACATAAGGAGCTGGAGGCTCC-TGAAAATATCTATCTAAATTAGA---ATTTAAATTATGAAATTGAAGTAGAAAGTCTCTTGGGGCTTTTTCCTTCAGTATA-TTGAG--GGCCTGAGCTTTGGACCCTGCGTTGATTGCCTCGGCATATGCGT-CGTTGGCAGTTTGGC----AACCTCCTGTAGCTGATCTTCCATCGACTTGGAAAACTCCATGATCAAGGATGTCTCCGTCTTTCTCCATGTAGGTTTGGACATC---GCTTGAGCTTTTAGCT-----CCCTGAATGTTCGGATGGAAAT--GTGCTGACCTAGTTGGGGAGGTGAGGTCGAAGAATCT-ATTGTTCCTGCACTGGAACTTTCCTTCGAACTGGAT-GAGAACACGCAAGT---GAGGATTCCCATCTTCATGAAGTTCTC-TGCAGATTC--------TAATGAATTTTTTGTTTACTGG--GGTTTGGAGATTTAAGAATTGGGAAAGTGCTTCTTCTTTAGTCAGGGA----GCACTTGGGATAAGTGAGAAAATAATTTTTGGAATATATTTGAAAGCGTTTGGAAGGCATG---------TTGACTACATTGA-GTAC----CGATTGACTCG------CTTTG----GCAACTCTCTCTGGTATATCGGTACC------CAA----------TATATA--GTGAG-CACCAAATGGCATAT-GTGTAA--------TTTT-GCAAT-GAAATTC-----AGAATCCTCA----C------GCT--CCAGGA---GC-GGCCAT--CCGTA-CAACATT

>BYVMV-IN:Har:05aazGU112081

-------ACCGGATGGCCGCGCGATTTTTTTAAG---TGGTGGGT-CCAG--AACGCAC-----------------------GACGATGCAGA--------CTC-----------AAAGCTTAGATAACG--------CTCCTTAGACTATCAGTACGTGCCCACTAAGTTTCAA---TTCAAAAA-----------ATGTGGGATCCACT------ATTAAACGAGTGCCCGGATACGGTTCACGGTTTTCGTTGTATGCTTTCTGTG--------AAATATTTGCA-------ACTTTTGTCGCAGGAGTA-TTCACCAGATAC---GCTTGGTTACGAT--TTAATACGGGATTTAATTTGTATTGTCCGTTCTCGTAATTATGTCGAAGCGAGCTGCAGATATCGTCATTTCTACGCCCGAATCGAAAGTACGCCGGCGTCTGAACTTCGGCAGCCCATACACCAGCCGTGCTGCTGCCCCCATT---GTCCGCG-----------------TCACAAAACAACA---GGCATGGACAAACAGGCCTATGAACAGGAAACCCAGAATGTAC-CGGATGTACAGAAGTCCGGATGTTCC-AAGGGGATGT----GAGGGTCCCTGTAAGGTACAGTCGTTTGAATCTC-GACACGATGTCGTTCATATT-GGTA-AGGTAATGTGTATTTCGGATGTTACGCGTGGAGTCGGTTTGACCCATCGTATAGG---TAAGCGTTTTTGTGTCA-AGTCA----GTTTATGTTTTAGGTAAGATCTGGATGGACGAGAACATCAAGTCCAAGAACCATACGAATTCGGTGATGTTTTTCC-TTGTTCGTGATCGACGACCGGTAGATAAA---CCACAAGATTTTGGT-GAAGTATTTAATATGTTTGATAACGAGCCCAGTACGGCGACCGTGAAGAACATGCATAGGGATCGGTACCAGGT-GTTGAGGA---AATGGCATGCA-----ACCGTTACTGGTGGACAATATGCGAGTAAGGAGCAGGCTTTGGTCAAGAAG-TTTGTTAGGGTTA-ATAACTACGT-TGTTTACAACCAG--------CAG-GAAGCAGGAAAATACGAGAATCACACCGAGAATGCATTGATGCTTTACATGGCTTGTACTCATGCTAGCAACCCAGTGTATGCTACGCTTAAGATTCGTATTTATTTTTATGACTCTG-------TAACGAACTAATA------------------TTAATAAAGTTTGAATTTTATATCTGAATATTGGTCTACATACAT-----TGTTTGATTAATTACATTGTACAATACATGTTCAACGGCTTTAATAACTAAATTAAGTGAGATTACACCTAGATTGTTGAGATACTTGAGGAC-------TTGGGTTTTGAAT---------------ACCCTTAAGAAAAGA-------------CCAGTCGGAG-GGTGTAAGGTC-----------GTCCAGATTC-GGAAGGTTAGAAAAC-ACTTGTGTATTCCC------AGAGCTTT----------CCGTAGGTTGTAGTTGAAATGGATCCTGAGTGTTATTATGTCCATGTTC--------GTCGTGAATGGACGGTTGTCGTGGTTGAGGATTTTGAAATAGAGGGGATTTGGAACCTTCC--------AGATA------------TAGACGCCATTCTTTGCTTGAGCT--GCAGTGATGAGTGCCCCTGTGCG--AGAATCCATGGTTGTGGCAGTTGATTGACAGATAA-------TAAGAACACCCGCATTCAAGATC-TACTCTCCTCCTCC---TGATGCGCCTCTTCGCTTCCCTGTGCTGTACTTTGA----TTGGTACCTGAGTACAGGGGTCCTTCAAGTGTGATGAAGAT---------------------------CGCATTCTTTACTGC------CCAGTTCTTTAGTGCGGTGTTCTTTTCCTCGTCTAGGAATTCTTTATAACT-GCTGTTGGGACCAGGATTGCAGAGGAAGATT-------------------------------------GTTGGTATCCCGCCTTTAA-TTTGAACTGGCTTCCCGTACTTTGTGTTGGATTGCCAGTCCCTT-----------------------TGGGCCCCCATGAACTCTTTAAAGTG--------TTTGAGGAAATGCGGGTCGACGTCATCAATGACA-TTA----------------TACCAGGCGTC-GTTACTGTAGAC---CTTGGGACTCAGGTCCAGATGTCCACACAAATAGTTATGTGGTCCCAGTGATCTAGCCCACATCGTCTTGCCGGT-------CTGACTGTCTCCCTCA-----------ATGTCCAAACTTTGAGGTCTCAGAGG-CCGCGCAGCGGCATCCATGACATTCTCGGAGACCCACTCTTCAAGTTCTTCAGGAACTTGATTAAAAGAAGAAGATAA------------------------------AAAAGGAGAAACATAAACCTCCAACGGAGGAGTGAAAAT-CCTATCTAAATTACT---ATTTAAATTATGATATTGAAAAATAAATTTTTCTGGGAGTTTTTCCCTTATTATAGCCAT---AGCTGCTTCTTTAGAACCTGCATTTAGGGCTTCTGCAGCAGCAT-CATTAGCTGTCTGTT----GACCTCCTCTTGCAGATCTTCCATCGATCTGAAATGTGCCCCAGTCGATGTAATCTCCGTCCTTCTCGATGTAGGACTTAACATCAGAGCTGGA---CTTAGCT-----CCCTGGAAGTTTGGATGGAATT--GGGTGGAGGTATGAGGGTGAGTGACATCGAAATGTCT-GGGGTTTCTGAACTTGGCTTTACCTTTGAATTGGAT-GAGTGCATGGATAT---GCAGAGACCCATCTTGGTGTTTTTCTTGTGCA-ACCC--------TAATGAATAATTTGTCTGAAGG--GCATGAAATGTTAGATAATATATCTAACATCTGTTCTTTTGGAATTGG----GCATTTTGGGAAAGTAAGAAAAATATTTTTTGCATTTATGCAAAAAGAATTATTTCTGGGC--ATT----TTTG---CTATCGGGGAC---ACTTAAAACTCA------TATCAATTGG------------GGACACTGGGGAC------TCA----------TTTATA--CTGCGTCCCTAAATGGCATAA-ATGTAAATATTTGCCTTT-ATGTTTGAATTTC-----AAATTCCAAA----C------GCT--CCAAAAA--GC-GGCCAT--CCGTA-TAATATT

>CLCuMuV-IN:Del:05aazAY765256

-------ACCGGATGGCTGCGC---GATTTT------TTGTGGGCCTTAC------CATTAAC------------ACTTGTCGGCCAATCATA-TGACTCCCTC-----------AAAGCTAAA-------T-AACGCTCCCGCACACTATAAGTACTTGCGCACTAAGTTTCAA---ATTCAAAC-----------ATGTGGGATCCACT------ATTAAACGAATTCCCTGATACGGTTCACGGGTTTCGGTGTATGCTTTCTGTG--------AAATATTTGCA-------ACTTTTGTCGCAGGATTA-TTCACCGGATAC---GCTTGGGTACGAG--TTAATACGGGATTTAATTTGTATTTTGCGCTCCCGTAATTATGTCGAAGCGAGCTGCCGATATCGTCATTTCTACGCCCGCGTCGAAAGTACGCCGGCGTCTGAACTTCGGCAGCCCATACACCAGCCGTGCTGCTGCCCCCATT---GTCCGCG-----------------TCACAAAACAACA---GGCATGGACAAACAGGCCTATGAACAGGAAGCCCAGGATGTAC-AGGATGTACAGAAGTCCAGATGTTCC-TAGAGGATGT----GAAGGTCCATGTAAGGTTCAGTCGTTTGAGTCCA-GACATGATATTCAGCATTTA-GGTA-AAGTAATGTGTGTTAGTGATGTTACTCGTGGTACTGGGCTGACCCATAGAGTTGG---TAAGAGATTTTGTGTCA-AGTCT----GTTTATGTGTTGGGTAAGATATGGATGGATGAGAACATTAAGACGAAGAATCACACGAATAGTGTGATGTTTTTCT-TGGTTAGAGATCGTAGACCTGTTGATAAA---CCTCAAGATTTTGGA-GAGGTATTTAATATGTTTGATAATGAGCCCAGTACGGCGACTGTGAAGAATGTTCATCGTGATAGGTATCAAGT-TCTGCGTA---AATGGTATGCA-----ACTGTCACCGGTGGACAATACGCTTCAAAGGAACAGGCTTTGGTCAAGAAG-TTTGTCAGAGTTA-ACAATTATGT-TGTTTACAATCAA--------CAG-GAAGCAGGAAAATACGAGAATCATACGGAAAATGCGTTAATGCTTTATATGGCTTGTACTCACGCTAGCAACCCTGTTTATGCTACGTTGAAGATTAGGATATATTTTTATGACTCTG-------TAACGAATTGATA------------------TTAATAAAGTTTGAATTTTATTTCTGAATATTGATCTACATACAT-----AGTTTGTTGGATTACATTGTACAATACATGTTCTACAGCTTAAATAACTAAATTAATTGAAATTACACCGAGATTGTTCAGATATTTGAGGAC-------TTGGGTTTTGAAT---------------ACCCTTAAGAAAAGA-------------CCAGTCTGAG-GGTGTAAGGTC-----------GTCCAGATTC-GGAAGGTTAGAAAAC-ACTTGCGCAGTCCC------AGAGCTTT----------CCGAGTGTTGTAGTTGAACTGGATCCTGATCGTGAGTATATCCATATTC--------GTCGTGAATGGACGGTTGACGTGGCTGATGATCTTGAAATAGAGGGGATTTGGAACCTCCC--------AGATA------------TATGCGCCATTCCCTGCTTGAGCT--GCAGTGATGGGTTCCCCTGTGCG--TGAATCCATGGTTGTGGCAGTTGATTGACAGATAA-------TAAGAACACCCGCATTCAAGATC-TACTCTCCTCCTCC---TGTTGCGTCTCTTCGCTTTCCTGTGCTGTACTTTGA----TTGGTACCTGAGTACAGGGGTCCTTCGAGAGTGATGAAGAT---------------------------CGCATTCTTTACTGC------CCAGTTTTTTAGTGCGGTGTTCTTTTCCTCGTCTAGGAATTCTTTATAACT-GCTGTTGGGACCAGGATTGCAGAGGAAGATT-------------------------------------GTTGGTATCCCGCCTTTAA-TTTGAACTGGCTTCCCGTACTTTGTGTTGGATCGCCAGTCCCTT-----------------------TGGGCCCCCATGAACTCTTTAAAGTG--------TTTGAGGAAATGCGGGTCGACGTCATCAATGACG-TTA----------------TACCAGGCGTC-GTTACTGTAGAC---CTTGGGACTCAGATCCAGATGTCCGCACAAATAGTTATGTGGTCCCAGTGATCTAGCCCACATCGTCTTTCCCGT-------TCGACTATCTCCTTCA-----------ATTACTATACTCCGAGGTCT-AAGGGGCCGCGCAGCGGGATCGACAACGTTATCGATGGCCCAAACTTCAAGTTCTTCTGGAACTTGATCGAAAGAAGAAGAAGA------------------------------AAAAGGAGAAATATAGGGAGCCGGTGGCTCCTGAAAGAT-TCTGTCTAGATTTGC---ATTTAAATTATGAAATTGTAGTATAAAATCTTTAGGAGCTAGTTCCTTAATGACT-CTAAG--AGCCTCTGACTTATTGCCTGCGTTAAGTGCTGCGGCGTAAGCGT-CGTTGGCTGTCTGTT----GTCCTCCTCTTGCTGATCTTCCATCGATCTGAAACTCTCCCCACTCGAGAGTGTCCCCGTCCTTGTCGATGTAGGCCTTGACATCTGAGCTTGA---TTTAGCT-----CCCTGAATGTTCGGATGGAAAT--GTGCTGACCTGGTTGGGGATACCAGGTCGAAGAATCT-TTTATTCGTGCATTGGTACTTCCCTTCGAACTGGAT-GAGCACGTGAAGAT---GAGGTTCCCCATTTTGGTGAAGCTCTC-TGCAGATCT--------TGATGTATTTTTTGTTTACTGG--TGTATGTAGGTTTAGTAATTGGGAGAGGGTTTCTTCTTTAGTTAGAGA----GCATTTGGGATAAGTGAGGAAGTAGTTTTTGGCGTTTGTTTTTAAAT------CACGTGGC--ATT----TTGG---CAATCG-GTGT---ACACT---CTAA------TTCTA--TGGCAA---TCG---GTGTAACGGGGTG------CAA----------TATATA-GGTGTA-CCCCAAATGGCATTA-TCGTAA--------TTTG-AGAAA-TCATTTC-----AAAATCCTCA----C------GCT--CCAAAAA--GC-GGCCAT--CCGTT-TAATATT

>CLCuMuV-IN:Bha:05aazDQ191160

-------ACCGGATGGCCGCGC--GATTTTT------TTGTGGGCCTTAC------CATTAAC------------ACTTGTCGGCCAATCATA-TGACTCCCTC-----------AAAGCTAAAT--------AACGCTCCCGCACACTATAAGTACTTGCGCACTAAGTTTCAA---ATTCAAAC-----------ATGTGGGATCCACT------ATTAAACGAATTCCCTGATACGGTTCACGGGTTTCGGTGTATGCTTTCTGTG--------AAATATTTGCA-------ACTTTTGTTGCAGGATTA-TTCACCGGATAC---GCTTGGGTACGAG--TTAATACGGGATTTAATTTGTATTTTACGCTCCCGTAATTATGTCGAAGCGAGCTGCCGATATCGTCATTTCTACGCCCGCGTCGAAAGTACGCCGGCGTCTGAACTTCGGCAGCCCATACACCAGCCGTGCTGCTGCCCCCATT---GTCCGCG-----------------TCACAAAACAACA---GGCATGGACAAACAGGCCTATGAACAGGAAGCCCAGGATGTAC-AGGATGTACAGAAGTCCAGATGTTCC-TAGAGGATGT----GAAGGTCCATGTAAGGTTCAGTCGTTTGAGTCCA-GACATGATATTCAGCATATA-GGTA-AAGTAATGTGTATTAGTGATGTTACTCGTGGTACTGGGCTGACCCATAGAGTTGG---TAAGAGATTTTGTGTCA-AGTCT----GTTTATGTGTTGGGTAAGATATGGATGGATGAGAACATTAAGACGAAGAATCACACGAATAGTGTGATGTTTTTCT-TGGTTAGAGATCGTAGACCTGTTGATAAA---CCTCAAGATTTTGGA-GAGGTATTTAATATGTTTGATAATGAGCCCAGTACGGCGACTGTGAAGAATGTTCATCGTGATAGGTATCAAGT-TCTGCGCA---AATGGTATGCA-----ACTGTCACCGGTGGACAATACGCTTCAAAGGAACAGGCTTTGGTCAAGAAG-TTTGTCAGAGTTA-ACAATTATGT-TGTTTACAATCAA--------CAG-GAAGCAGGAAAATACGAGAATCATACGGAAAATGCGTTAATGCTTTATATGGCTTGTACTCACGCTAGCAACCCTGTTTATGCTACGTTGAAGATTAGGATATATTTTTATGACTCTG-------TAACGAATTGATA------------------TTAATAAAGTTTGAATTTTATTTCTGAATATTGATCTACATACAT-----AGTTTGTTGGATTACATTGTACAATACATGTTCTACAGCTTTAATAACTAAATTAATTGAAATTACACCGAGATTGTTCAGATATTTGAGGAC-------TTGGGTTTTGAAT---------------ACCCTTAAGAAAAGA-------------CCAGTCTGAG-GGTGTAAGGTC-----------GTCCAGATTC-GGAATGTTAGAAAAC-ACTTGTGCAGTCCC------AGAGCTTT----------CCGAGTGTTGTAGTTGAACTGGATCCTGATCGTGAGTATATCCATATTC--------GTCGTGAATGGACGGTTGACGTGGCTGATGATCTTGAAATAGAGGGGATTTGGAACCTCCC--------AGATA------------TATGCGCCATTCCCTGCTTGAGCT--GCAGTGATGGGTTCCCCTGTGCG--TGAATCCATGATTGTGGCAGTTGATTGACAGATAA-------TAAGAACACCCGCATTCAAGATC-TACTCTCCTCCTCC---TGTTGCGTCTCTTCGCTTCCCTGTGCTGTACTTTGA----TTGGTACCTGAGTACAGGGGTCCTTCGAGAGTGATGAAGAT---------------------------CGCATTCTTTACTGC------CCAGTTTTTTAGTGCGGTGTTCTTTTCCTCGTCTAGGAACTCTTTATAACT-GCTGTTGGGACCAGGATTGCAGAGGAAGATT-------------------------------------GTTGGTATCCCGCCTTTAA-TTTGAACTGGCTTCCCGTACTTTGTGTTGGATTGCCAGTCCCTT-----------------------TGGGCCCCCATGAACCCTTTAAAGTG--------TTTGAGGAAATGCGGGTCGACGTCATCAATGACG-TTA----------------TACCAGGCGTC-GTTACTGTAGAC---CTTGTGACTCAGATCCAGATGTCCGCACAAATAGTTATGTGGTCCCAGTGATCTAGCCCACATCGTCTTTCCCGT-------TCGACTATCTCCTTCA-----------ATTACTATACTCCGAGGTCT-AAGGGGCCGCGCAGCGGGATCGACAACGTTATCGATGGCCCAAACTTCAAGTTCTTCTGGAACTTGATCGAAAGAAGAAGAAGA------------------------------AAAAGGAGAAATATAGGGAGCCGGTGGCTCCTGAAAGAT-TCTGTCTAGATTTGC---ATTTAAATTATGAAATTGTAGTACAAAATCTTTAGGAGCTAGTTCCTTAATGACT-CTAAG--AGCCTCTGACTTATTGCCTGCGTTAAGTGCTGCGGCGTAAGCGT-CGTTGGCTGTCTGTT----GTCCTCCTCTTGCTGATCTTCCATCGATCTGAAACTCTCCCCACTCGAGAGTGTCCCCGTCCTTGCCGATGTAGGCCTTGACATCTGAGCTTGA---TTTAGCT-----CCCTGAATGTTCGGATGGAAAT--GTGCTGACCTGGTTGGGGATACCAGGTCGAAGAATCT-TTTATTCGTGCATTGGTACTTCCCCTCGAACTGGAT-GAGCACGTGAAGAT---GAGGTTCCCCATTTTGGTGAAGCTCTC-TGCAGATCT--------TGATGTATTTTTTGTTTACTGG--TGTATGTAGGTTTAGTAATTGGGAGAGGGTTTCTTCTTTAGTTAGAGA----GCATTTGGGATAAGTGATGAAATAGTTTTTGGAATAAAAACCGTTCCGCTTTGGAGGCATG---------TTGACTAAAATTG-ATCA---CCGATTGACCGC------TCTTG-----CAACTCTCCCCGGTATATCGGTGAT------CAA----------TATATA--GTGAT-CACCAAATGGCATAA-TGGTAA--------TAAA-AAAACTTTAATTT-----GAAATTCAAA----CCAAAAGGCT------AAA--GC-GGCCAT--CCGTT-TAATATT

>CLCuMuV-CN:Gua:08aazGQ503175

-------ACCGGATGGCCGCGC--GATTTTT------TTGTGGGCCCCCT---------------------------------ATTTATGAGA-TTGCTCCCTC-----------AAAGTTAAAT--------AACGCTCCCGCCCACTATAAGTACTTGCGCACTAAGTTTCAA---ATTCAAAC-----------ATGTGGGATCCATT------GTTAAACGAATTTCCTGATACGGTGCACGGGTTTCGGTGTATGCTTTCTGTC--------AAATATTTGCA-------ACTTTTGTCGCAGGATTA-TTCACCGGATAC---CCTAGGTTACGAT--TTAATACGGGATTTAATCTGTATTCTACGTTCCCGTAATTATGTCGAAGCGAGCTGCAGATATCGTCATTTCAACGCCCGCGTCGAAAGTACGTCGGCGTCTGAACTTCGGCAGCCCATACACCAACCGTGTTGCTGTCCCCATT---GTCCGCG-----------------TCACAAAACAACA---GGCATGGACAAACAGGCCTATGAACAGGAAGCCCAGAATATAT-CGGATGTACAGAAGTCCGGATGTTCC-AAAGGGTTGT----GAAGGCCCATGTAAGGTACAGTCTTTTGAGTCCA-GACATGATGTTGTTCATATT-GGTA-AGGTAATGTGTATTTCTGATGTTACTCGTGGTGTCGGTTTGACCCATCGTATTGG---TAAACGTTTTTGTGTCA-AGTCA----GTTTATGTTTTAGGTAAGATATGGATGGATGAAAATATAAAGACCAGGAATCACACGAATTCGGTCATGTTCTTTT-TAGTTCGCGATCGACGACCTGTTGACAAA---CCTCAGGATTTTGGT-GAGGTATTCAATATGTTTGATAACGAACCCAGTACAGCAACTGTGAAGAATAGTCATAGGGACCGTTATCAGGT-GTTGAGGA---AATGGCATGCA-----ACCGTTACGGGTGGTCAATATGCGAGTAAGGAACAGGCTTTGGTCAAGAAG-TTTGTCAGAGTTA-ACAATTATGT-TGTTTACAATCAA--------CAG-GAAGCAGGAAAATACGAGAATCATACGGAAAATGCGTTAATGCTTTATATGGCTTGTACCCACGCTAGCAACCCTGTTTATGCTACGTTGAAGATTAGGATATATTTTTATGACTCTG-------TAACGAATTGATG------------------TTAATAAAGTTTGAATTCTATTTCTGAATATTGATCTACATACAT-----AGTTTGTTGGATTACATTGTACAATACATGTTCTACAGCTTTAATAACTAAATTAATTGAAATTACACCGAGATTGTTCAGATATTTGAGGAC-------TTGGGTTTTGAAT---------------ACCCTTAAGAAAAGA-------------CCAGTCTGAG-GGTGTAAGGTC-----------GTCCAGATTC-GGAAGGTTAGAAAAC-ACTTGTGCAGTCCC------AGAGCTTT----------CCGAGTGTTGTAGTTGAATTGGATTCGGATCGTGAGTATGTCCATATTT--------GTCGTGAATGGACGGTTGAAGTGGCTGATTATCTTGAAATAAAGGGGATTTGGAACCTCCC--------AGATA------------TATGCGCCATTCCCTGCTTGAGCT--GCAGTGATGGGTTCCCCTGTGCG--TGAATCCATGGTTGTGGCAGTTGATTGACAGATAA-------TAAGAACACCCGCATTCAAGATC-TACTCTCCTCCTCC---TGTTGCGTCTCTTCGCTTCCCTGTGCTGTACTTTGA----TTGGAAGCTGAGTACAGTGGTCCTTCGAGAGTGATGAAGAT---------------------------CGCATTTTTTAGGGC------CCAATTCTTTAATGCTGTGTTTTTTTCCTCGTTGAGGAATTCCTTATAACT-GCTGTTGGGACCAGGATTGCACAAGAAGATT-------------------------------------GTCGGTATTCCGCCTTTAA-TTTGAACTGGCTTACCGTATTTTGTGTTGGACTGCCAGTCTCTT-----------------------TGGGCCCCCATGAACTCTTTAAAGTG--------TTTGAGGAAATGCGGGTCGACGTCATCAATGACG-TTG----------------TACCAGGCGTC-GTTACTGTATAC---TTTGGGACTCAGGTCCAGATGTCCGCACAAATAGTTATGTGGTCCCAATGACCTAGCCCACATCGTCTTTCCCGT-------ACGACTATCTCCCTCA-----------ATTACTATACTCCGAGGTCT-AAGGGGCCGCGCAGCGGCATCGACAACGTTATCCAACGCCCAAACTTGAAGTTCTTCTGGAACTTGATCGAACGAAGAAAAAGA------------------------------AAAAGGAGAAACATAAGGAGCTGGTGGCTCCTGAAAGAT-CCTGTCTAGATTTGC---ATTTAAATTATGAAATTGTAGTACAAAATCTTTAGGAGCTAGTTCCTTAATGACT-CTAAG--AGCCTCTGACTTACTTCCCGCGTTAAGCGCTGCGGCGTAAGCGT-CGTTGGCTGTCTGTT----GTCCTCCTCTTGCTGATCTTCCATCGATCTGAAACTCTCCCCACTCGAGAGTGTCCCCGTCCTTGTCGATGTAGTCCTTGACATCTGAGCTTGA---TTTAGCT-----CCCTGAATGTTCGGATGGAAAT--GTGCTGACCTGGTTGGGGATACCAGGTCGAAGAATCT-GTTATTCGTGCAGATGAGTTTGCCCTCGAACTGGAT-GAGCACATGGAGAT---GAGGGCTCCCATCTTCGTGTAACTCTC-TGCAGACTT--------TGATGTATTTTTTATTCGAGGG--TGTGTTGATGGCTTGAATTTGGGAAAGTGCTTCCTCTTTAGTGAGTGA----GCACTGTGGATAAGTGAGGAAATAATTTTTGGCTTGTATTTTAAAACGTTTGGGGGGAGCC--ATTGACTTTGGT--CAATTG-GAGA----CAACTGATTGG----ATTTTACTCTGGCAA---TT----GGAGACTGGAGA-------CAA----------TTTATA--GTGT--CTCCAAATGGCATTA-TCGTAA--------TTTGAGAAA--TCATTTC-----AAAATCCTCA----C------GCT--CCAAAAA--GC-GGCCAT--CCGTA-TAATATT

>CLCuMuV-CN:G6:08aazEF465535

-------ACCGGATGGCCGCGC--GATTTTT------TTGTGGGCCCCCT--ATT-------------------------------TATGAGA-TTGCTCCCTC-----------AAAGCTAAA-------T-AACGCTCCCGCCCACTATAAGTACTTGCGCACTAAGTTTCAA---ATTCAAAC-----------ATGTGGGATCCATT------GTTAAACGAATTTCCTGATACGGTGCACGGGTTTCGGTGTATGCTTTCTGTC--------AAATATTTGCA-------ACTTTTGTCGCAGGATTA-TTCACCGGATAC---CCTAGGTTACGAT--TTAATACGGGATTTAATCTGTATTCTACGTTCCCGTAATTATGTCGAAGCGAGCTGCAGATATCGTCATTTCAACGCCCGCGTCGAAAGTACGTCGGCGTCTGAACTTCGGCAGCCCATACACCAACCGTGTTGCTGTCCCCATT---GTCCGCG-----------------TCACAAAACAACA---GGCATGGACAAACAGGCCTATGAACAGGAAGCCCAGAATATAT-CGGATGTACAGAAGTCCGGATGTTCC-AAAGGGTTGT----GAAGGCCCATGTAAGGTACAGTCTTTTGAGTCCA-GACATGATGTTGTTCATATT-GGTA-AGGTAATGTGTATTTCTGATGTTACTCGTGGTGTCGGTTTGACCCATCGTATTGG---TAAACGTTTTTGTGTCA-AGTCA----GTTTATGTTTTAGGTAAGATATGGATGGATGAAAATATAAAGACCAGGAATCACACGAATTCGGTCATGTTCTTTT-TAGTTCGCGATCGACGACCTGTTGACAAA---CCTCAGGATTTTGGT-GAGGTATTCAATATGTTTGATAACGAACCCAGTACAGCAACTGTGAAGAATAGTCATAGGGACCGTTATCAGGT-GTTGAGGA---AATGGCATGCA-----ACCGTTACGGGTGGTCAATATGCGAGTAAGGAACAGGCTTTGGTCAAGAAG-TTTGTCAGAGTTA-ACAATTATGT-TGTTTACAATCAA--------CAG-GAAGCAGGAAAATACGAGAATCATACGGAAAATGCGTTAATGCTTTATATGGCTTGTACCCACGCTAGCAACCCTGTTTATGCTACGTTGAAGATTAGGATATATTTTTATGACTCTG-------TAACGAATTGATG------------------TTAATAAAGTTTGAATTCTATTTCTGAATATTGATCTACATACAT-----AGTTTGTTGGATTACATTGTACAATACATGTTCTACAGCTTTAATAACTAAATTAATTGAAATTACACCGAGATTGTTCAGATATTTGAGGAC-------TTGGGTTTTGAAT---------------ACCCTTAAGAAAAGA-------------CCAGTCTGAG-GGTGTAAGGTC-----------GTCCAGATTC-GGAAGGTTAGAAAAC-ACTTGTGCAGTCCC------AGAGCTTT----------CCGAGTGTTGTAGTTGAACTGGATTCGGATCGTGAGTATGTCCATATTC--------GTCGTGAATGGACGGTTGAAGTGGCTGATTATCTTGAAATAAAGGGGATTTGGAACCTCCC--------AGATA------------TATGCGCCATTCCCTGCTTGAGCT--GCAGTGATGGGTTCCCCTGTGCG--TGAATCCATGGTTGTGGCAGTTGATTGACAGATAA-------TAAGAACACCCGCATTCAAGATC-TACTCTCCTCCTCC---TGTTGCGTCTCTTCGCTTCCCTGTGCTGTACTTTGA----TTGGAAGCTGAGTACAGTGGTCCTTCGAGAGTGATGAAGAT---------------------------CGCATTTTTTAGGGC------CCAATTCTTTAATGCTGTGTTTTTTTCCTCGTTGAGGAATTCCTTATAACT-GCTGTTGGGACCAGGATTGCACAAGAAGATT-------------------------------------GTCGGTATTCCGCCTTTAA-TTTGAACTGGCTTACCGTATTTTGTGTTGGACTGCCAGTCTCTT-----------------------TGGGCCCCCATGAACTCTTTAAAGTG--------TTTGAGGAAATGCGGGTCGACGTCATCAATGACG-TTG----------------TACCAGGCGTC-GTTACTGTATAC---TTTGGGACTCAGGTCCAGATGTCCGCACAAATAGTTATGTGGTCCCAATGACCTAGCCCACATCGTCTTTCCCGT-------ACGACTATCTCCCTCA-----------ATTACTATACTCCGAGGTCT-AAGGGGCCGCGCAGCGGCATCGACAACGTTATCCAACGCCCAAACTTGAAGTTCTTCTGGAACTTGATCGAACGAAGAAAAAGA------------------------------AAAAGGAGAAACATAAGGAGCTGGTGGCTCCTGAAAGAT-CCTGTCTAGATTTGC---ATTTAAATTATGAAATTGTAGTACAAAATCTTTAGGAGCTAGTTCCTTAATGACT-CTAAG--AGCCTCTGACTTACTTCCCGCGTTAAGCGCTGCGGCGTAAGCGT-CGTTGGCTGTCTGCT----GTCCTCCTCTTGCTGATCTTCCATCGATCTGAAACTCTCCCCACTCGAGAGTGTCCCCGTCCTTGTCGATGTAGTCCTTGACATCTGAGCTTGA---TTTAGCT-----CCCTGAATGTTCGGATGGAAAT--GTGCTGACCTGGTTGGGGATACCAGGTCGAAGAATCT-GTTATTCGTGCAGATGAGTTTGCCCTCGAACTGGAT-GAGCACATGGAGAT---GAGGGCTCCCATCTTCGTGTAACTCTC-TGCAGACTT--------TGATGTATTTTTTATTCGAGGG--TGTGTTGATGGCTTGAATTTGGGAAAGTGCTTCCTCTTTAGTGAGTGA----GCACTGTGGATAAGTGAGGAAATAATTTTTGGCTTGTATTTTAAAACGTTTGGGGGGAGCC--ATTGACTTTGGT--CAATTG-GAGACAACTGATTGGATTT------TACTC--TGGCAA---TT----GGAGACTGGAGA-------CAA----------TTTATA--GTGT--CTCCAAATGGCATTA-TCGTAA--------TTTG-AGAAA-TCATTTC-----AAAATCCTCA----C------GCT--CCAAAAA--GC-GGCCAT--CCGTA-TAATATT

>CLCuBuV-IN:Pun:07aazFN645932

-------ACCGGATGGCCGCGC--GATTTTT------TTGTGGGCCCTAC------CATTAACT------------CTTGTCGGCCAATCATATGACGC-GCTC-----------AAAGCTTAAATAATT-------CTCCCGCCTATTATAAGTACTTCGTTGCTAAGTATGCG---TTTGAAAA-----------ATGTGGGATCCACT------GTTAAATGAGTTCCCCGACACCGTTCACGGTTTTAGGTGTATGTTAGCAGTT--------AAATATTTGCA-------GTTAGTAGAGAAAACTTA-CTCTCCTGATAC---ATTGGGTTACGAT--TTGATAAGGGATTTAATCCTGGTAATAAGGGCTAGGAATTATGTCGAAGCGACCAGCAGATATAATCATTTCCACGCCCGCTTCGAAGGTACGCCGCCGTCTCAACTTCGACAGCCCATATGTGAGCCGTGCTGCTGCCCCCATT---GTCCGCG-----------------TCACCAAAGCAAA---AGCATGGGCGAACAGGCCCATGAACAGAAAGCCCAGGATGTAC-AGGATGTACAGAAGTCCAGATGTTCC-TAGAGGATGT----GAAGGTCCATGTAAGGTTCAGTCGTTTGAGTCCA-GACATGATATTCAGCATATA-GGTA-AAGTAATGTGTGTTAGTGATGTTACTCGTGGTACTGGGCTGACCCATAGAGTTGG---TAAGAGATTTTGTGTTA-AGTCT----GTTTATGTGTTGGGTAAGATCTGGATGGATGAGAACATTAAGACGAAGAATCACACGAATAGTGTGATGTTTTTCT-TGGTTAGAGATCGTAGACCTGTTGATAAA---CCTCAAGATTTTGGA-GAGGTATTTAATATGTTTGATAATGAGCCCAGTACGGCGACTGTGAAGAATGTTCATCGTGATAGGTATCAAGT-TCTGCGCA---AATGGTATGCA-----ACTGTCACCGGTGGACAATACGCTTCAAAGGAACAAGCTCTCGTGAAGAAA-TTTATTAGAGTTA-ATAATTATGT-TGTGTATAACCAG--------CAG-GAAGCTGGCAAGTATGAGAATCATTCTGAGAATGCTTTAATGTTGTATATGGCGTGTACTCACGCCTCTAACCCAGTGTATGCTACCTTGAAGATACGGATCTACTTCTATGATTCCG-------TGACAAA------------------------TTAATAAATATTGAATTTTATTGAAGATGATTGGTCTACAAATAC-----AACATGTTGTAATACATTCCATAATACATGATCAACTGCTCTAACTACATTATTAATACTGACAATTCCTAAGTTATTTAAATATTTAAGCAC-------TTGAGTCCTAAAG---------------ACCCTTAAGAAACGA-------------CCAGTCGGAG-GCTGTGAGGTC-----------ATCCAGATTC-GGAAAGCTATGAAAC-ATTTGTGTATCCCC------AACGCTTT----------CCTCAGGTTGTGATTGAACTGGATCCTGATCGTGAGTATATCCATATTC--------GTCGTGAATGGACGGTTGACGTGGCTGATGATCTTGAAATAGAGGGGATTTGGAACTTCCC--------AGATA------------TATGCGCCATTCCCTGCTTGAGCT--GCAGTGATGGGTTCCCCTGTGCG--TGAATCCATGGTTGTGGCAGTTGATTGACAGATAC-------TAAGAACACCCTCATTCAAGATC-TACTATCCTCCTCC---TGTTGCGTCTCTTCGCTTCCCTGTGCTGTACTTTGA----TTGGAAGCTGAGTACAGTGGTCCTTCGAGAGTGATGAAGAT---------------------------TGCATTTTTTAAGGT------CCAATTCTTTAATGCTGTGTTTTTTTCCTCGTTGAGGAATTCATTATAACT-GCTGTTCGGACCAGGATTGCACAGGAAGATT-------------------------------------GTCGGTATCCCGCCTTTAA-TTTGAACTGGCTTTCCGTATTTTGTGTTGGACTGCCAGTCTCTT-----------------------TGGGCCCCCATGAACTCTTTAAAGTG--------TTTGAGGAAATGCGGGTCGACGTCATCAATGACG-TTG----------------TACCAGGCGTC-GTTACTGTATAC---TTTGGGACTCAGGTCCAGATGTCCGCACAAATAGTTATGTGGTCCCAATGATCTAGCCCACATCGTCTTCCCCGT-------TCGACTATCTCCCTCA-----------ATTACTATACTCCGAGGTCT-AAGGGGCCGCGCAGCGGCATCGACAACGTTATCGATGGCCCAAACTTCAAGTTCTTCTGGAACTTGATCGAAAGAAGAAGGAGA------------------------------AAAAGGAGAAATATAGGGAGCCGGTGGCTCCTGAAAGAT-TCTATCTAGATTTGC---ATTTAAATTATGAAATTGTAGTACAAAATCTTTAGGAGCTAGTTCCTTAATGACT-CTAAG--AGCCTCTGACTTACTGCCTGCGTTAAGTGCTGCGGCGTAAGCGT-CGTTGGCTGTCTGCT----GTCCTCCTCTTGCTGATCTTCCATCTATCTGAAACTCTCCCCACTCGAGAGTGTCCCCGTCCTTGTCGATGTAGGCCTTGACATCTGAGCTTGA---TTTAGCT-----CCCTGAATGTTCGGATGGAAAT--GTGCTGACCTGGTTGGGGATACCAGGTCGAAGAATCT-GTTATTCGTGCAGACGAATTTGCCCTCGAACTGGAT-GAGCACATGGAGAT---GAGGGCTCCCATCTTCGTGTAACTCTC-TGCAGAGTT--------TGATGTATTTTTTATTCGAGGG--TGTGTTGATGGCTTGAATTTGGGAAAGTGCTTCCTCTTTAGTGAGTGA----GCACTGTGGATAAGTGATGAAATAGTTTTTGGCTTGTACTTTAAAACGTTTGGGGGGAGCC--ATTGACTTTGGT--CAATTA-GAGA----CAACTGATGGG------CTTT------------TACTCTGGGAATTGGAGACTGGATACAA----------TTTATA--GTGT--CTCCAAATGGCATATTCTGTAAA-------TAAC-TAGAAGTTCGTTT-----GAAATTCAAATTCCCCTTTGGGGT--CCAAAA---GC-GGCCAT--CCGTA-TAATATT

>CLCuBuV-IN:Pun:07aazFN645929

-------ACCGGATGGCCGCGC--GATTTTT------TTGTGGGCCCTAC------CATTAACT------------CTTGTCGGCCAATCATATGACGC-GCTC-----------AAAGCTTAAAAAATT-------CTCCCGCGTATTATAAGTACTTCGTTGCTAAGTATGCG---TTTGAAAA-----------ATGTGGGATCCACT------GTTAAATGAGTTCCCCGACACCGTTCACGGTTTTAGGTGTATGTTAGCAGTT--------AAATATTTGCA-------GTTAGTAGAGAAAACTTA-CTCTCCTGATAC---ATTGGGTTACGAT--TTGATAAGGGATTTAATCCTGGTAATAAGGGCTAGGAATTATGTCGAAGCGACCAGCAGATATAATCATTTCCACGCCCGCTTCGAAGGTACGCCGCCGTCTCAACTTCGACAGCCCATATGTGAGCCGTGCTGCTGCCCCCATT---GTCCGCG-----------------TCACCAAAGCAAA---AGCATGGGCGAACAGGCCCATGAACAGAAAGCCCAGGATGTAC-AGGATGTACAGAAGTCCAGATGTTCC-TAGAGGATGT----GAAGGTCCATGTAAGGTTCAGTCGTTTGAGTCCA-GACATGATATTCAGCATATA-GGTA-AAGTAATGTGTGTTAGTGATGTTACTCGTGGTACTGGGCTGACCCATAGAGTTGG---TAAGAGATTTTGTGTTA-AGTCT----GTTTATGTGTTGGGTAAGATCTGGATGGATGAGAACATTAAGACGAAGAATCACACGAATAGTGTGATGTTTTTCT-TGGTTAGAGATCGTAGACCTGTTGATAAA---CCTCAAGATTTTGGA-GAGGTATTTAATATGTTTGATAATGAGCCCAGTACGGCGACTGTGAAGAATGTTCATCGTGATAGGTATCAAGT-TCTGCGCA---AATGGTATGCA-----ACTGTCACCGGTGGACAATACGCTTCAAAGGAACAAGCTCTCGTGAAGAAA-TTTATTAGAGTTA-ATAATTATGT-TGTGTATAACCAG--------CAG-GAAGCTGGCAAGTATGAGAATCATTCTGAGAATGCTTTAATGTTGTATATGGCGTGTACTCACGCCTCTAACCCAGTGTATGCTACCTTGAAGATACGGATCTACTTCTATGATTCCG-------TGACAAA------------------------TTAATAAATATTGAATTTTATTGAAGATGATTGGTCTACAAATAC-----AACATGTTGTAATACATTCCATAATACATGATCAACTGCTCTAACTACATTATTAATACTGACAATTCCTAAGTTATTTAAATATTTAAGCAC-------TTGAGTCCTAAAG---------------ACCCTTAAGAAACGA-------------CCAGTCGGAG-GCTGTGAGGTC-----------ATCCAGATTC-GGAAAGCTATGAAAC-ATTTGTGTATCCCC------AACGCTTT----------TCTCAGGTTGTGATTGAACTGGATCCTGATCGTGAGTATATCCATATTC--------GTCGTGAATGGACGGTTGACGTGGCTGATGATCTTGAAATAGAGGGGATTTGGAACTTCCC--------AGATA------------TATGCGCCATTCCCTGCTTGAGCT--GCAGTGATGGGTTCCCCTGTGCG--TGAATCCATGGTTGTGGCAGTTGATTGACAGATAC-------TAATAACACCCTCATTCAAGATC-TATTCTCTTCCTCC---TGTTGCGTCTCTTCGCTTCCCTGTGCTGTACTTTGA----TTGGAAGCTGAGTACAGTGGTCCTTCGAGAGTGATGAAGAT---------------------------TGCATTTTTTAAGGC------CCAATTCTTTAATGCTGTGTTTTTTTCCTCGTTGAGGAATTCATTATAACT-GCTGTTCGGACCAGGATTGCACAGGAAGATT-------------------------------------GTCGGTATCCCGCCTTTAA-TTTGAACTGGCTTTCCGTATTTTGTGTTGGACTGCCAGTCTCTT-----------------------TGGGCCCCCATGAACTCTTTAAAGTG--------TTTGAGGAAATGCGGGTCGACGTCATCAATGACG-TTG----------------TACCAGGCGTC-GTTACTGTATAC---TTTGGGACTCAGGTCCAGATGTCCGCACAAATAGTTATGTGGTCCCAATGATCTAGCCCACATCGTCTTCCCCGT-------TCGACTATCTCCCTCA-----------ATTACTATACTCCGAGGTCT-AAGGGGCCGCGCAGCGGCATCGACAACGTTATCGATGGCCCAAACTTCAAGTTCTTCTGGAACTTGATCGAAAGAAGAAGGAGA------------------------------AAAAGGAGAAATATAGGGAGCCGGTGGCTCCTGAAAGAT-TCTATCTAGATTTGC---ATTTAAATTATGAAATTGTAGTACAAAATCTTTAGGAGCTAGTTCCTTAATGACT-CTAAG--AGCCTCTGACTTACTGCCTGCGTTAAGTGCTGCGGCGTAAGCGT-CGTTGGCTGTCTGCT----GTCCTCCTCTTGCTGATCTTCCATCTATCTGAAACTCTCCCCACTCGAGAGTGTCCCCGTCCTTGTCGATGTAGGCCTTGACATCTGAGCTTGA---TTTAGCT-----CCCTGAATGTTCGGATGGAAAT--GTGCTGACCTGGTTGGGGATACCAGGTCGAAGAATCT-GTTATTCGTGCAGACGAATTTGCCCTCGAACTGGAT-GAGCACATGGAGAT---GAGGGCTCCCATCTTCGTGTAACTCTC-TGCAGAGTT--------TGATGTATTTTTTATTCGAGGG--TGTGTTGATGGCTTGAATTTGGGAAAGTGCTTCCTCTTTAGTGAGTGA----GCACTGTGGATAAGTGATGAAATAGTTTTTGGCTTGTACTTTAAAACGTTTGGGGGGAGCC--ATTGACTTTGGT--CAATTA-GAGA----CAACTGATGGG------CTTT------------TACTCTGGGAATTGGAGACTGGATACAA----------TTTATA--GTGT--CTCCAAATGGCATATTCTGTAAA-------TAAC-TAGAAGTTCGTTT-----GAAATTCAAATTCCCCTTTGGGGT--CCAAAA---GC-GGCCAT--CCGTA-TAATATT

>CLCuBaV-IN:Ban:04aazAY705380

-------ACCGGATGGCCGCGC--GATTTTT------TTGTGGGTCCCGC------CACTAAC------------ACTTGTCTGCCAATCACATGGCGC-GCTC-----------AAAGCTTAAATAATT--------TCCCGCTTATTATAAGTACTTCGTCCCTAAGTTTCTG---TTTGAAAA-----------ATGTGGGATCCACT------GTTAAACGAGTTCCCCGAGACGGAACACGGGTTTCGTTGCGTGCTTGCTATC--------AAATATCTTCA-------ACAATTGTCTGAAGAATA-CTCCCCTGATAC---GCTAGGTTACGAT--TTAATCCGCGATTTAATGTCAATTTTGCGTTCTAGGAATTATGTCGAAGCGTCCTGCCGATATCGTCATTTCTACCCCCGCGTCGAAGGTGCGTCGGCGTCTGAACTTCGACAGCCCTTATGCAACCCGTGCAGTTGTCCCCACT---GTCCGCG-----------------TCACAAAATCTCG---CATGTGGGCGAACAGGCCCATGAACCGCAAGCCCAGAATGTAC-AGGATGTACAGAAGCCTTGATGTTCC-AAGAGGCTGT----GAGGGTCCATGTAAGGTCCAGTCGTTTGAGTCTA-GACACGATGTAGTCCATATA-GGTA-AGGTCATGTGCATTAGTGATGTTACACGTGGTACTGGGTTAACCCATAGAGTTGG---TAAGCGTTTTTGTGTGA-AGTCT----GTCTATGTTTTGGGCAAGATATGGATGGATGAGAACATCAAGACCAAGAATCACACGAACAGTGTCATTTTTTTTC-TTGTTCGTGACCGTCGTCCTGTTGACAAG---CCACAAGATTTTGGA-GAGGTGTTCAATATGTTTGACAACGAGCCTAGTACTGCTACGGTGAAGAATATGCATAGAGATCGTTATCAGGT-GCTGAGGA---AGTGGCATGCA-----ACTGTTACTGGTGGACAGTACGCCTCAAAGGAACAGGCATTAGTTAAGAAG-TTTGTTAGGGTTA-ATAATTATGT-TGTTTATAACCAG--------CAA-GAGGCTGGGAACTATGAGAATCATTCTGAGAATGCTTTGATGTTGTATATGGCATGTACCCATGCCTCAAATCCTGTATACGCTACGCTTAAGATCCGTATTTATTTCTATGATTCCG-------TAACCAATTGATA------------------TTAATAAAGATCGAATTTTATTTCTGAAGTTTGGTCTACAAACAT-----GGTGTTTTCTATTTTCTTGTACAATACATGATCAACTGCTCTAATAATTGAATTAATTGAGATTACACCTAGATTGTTGAGGTACTTGAGGAC-------CTGTGTTTTGAAT---------------ACCCTTAAGAAAAGA-------------CCAGTCGGAG-GGTGTAAGGTC-----------GTCCAGATTC-GGAAGGTCAGAAAAC-ACTTGTGCACTCCC------AGAGCTCT----------CCGAAGGTTGTAATTGAATTGGATTCTGATCGTTATTATGTCCATGTTG--------TTTGTGAATGGCCGGTTGGGGAGGCTTAGGATCTTGAAATATAGGGGATTTGGAACTTCCG--------AGATA------------TAGACGCCACTCCATGCTTGAGCT--GCAGTGATGGGTTCCCCTGTGCG--TGAATCCATGGTTGTGGCAGTTGATAGACAAATAA-------TAAGAACACCCGCATTCAAGATC-TACTCTCCTCCTCC---TGTTGCGTCTCTTCGCTTCCCTGTGCTGAACTTTGA----TTGGTACCTGAGTACAATGGTCCTTCAAGGGTGATGAAGAT---------------------------CGCATTCTTTACTGC------CCAGTTCTTTAGTGCTGTGTTCTTTTCCTCGTCGAGAAATTCTTTATAACT-GCTGTTGGGACCTGGATTGCAGAGGAAGATT-------------------------------------GTCGGTATCCCGCCTTTAA-TTTGAACTGGCTTCCCGTACTTTGTGTTGGATTGCCAGTCCCTT-----------------------TGGGCCCCCATGAACTCCTTAAAGTG--------CTTGAGGAAATGCGGATCGACGTCATCAATGACG-TTA----------------TACCAGGCGTC-GTTATTGTAGAC---CTTGGGACTCAGGTCCAGATGTCCGCATAAATAGTTATGTGGTCCCAGTGATCTGGCCCACATCGTCTTCCCCGT-------TCGACTATCTCCCTCA-----------ATTACTATACTTCGAGGTCT-AAGGGGCCGCGCAGCGGCATCGACAACGTTATCGATGGCCCAAACTTCAAGTTCTTCTGGAACTTGATCAAAAGAAGAAAAAGA------------------------------AAAAGGAGAAACATAAGGAGCTGGTGGCTCCTGAAAGAT-CCTGTCTAGATTTGC---ATTTAAATTATGAAATTGTAGTACAAAATCTTTAGGAGCTAGTTCCTTAATGACT-CTAAG--AGCCTCTGACTTACTTCCCGCGTTAAGCGCTGCGGCGTAAGCGT-CGTTGGCTGTCTGTT----GTCCTCCTCTTGCTGATCTTCCATCGATCTGAAACTCTCCCCACTCGAGAGTGTCTCCGTCCTTGTCGATGTAGTCCTTGACATCTGAGCTTGA---TTTAGCT-----CCCTGAATGTTCGGATGGAAAT--GTGCTGACCTGGTTGGGGATACCAGGTCGAAGAATCT-CTGATTTTGGCACTTGTACTTCCCTTCGAACTGGAT-GAGCACGTGAATAT---GAGGTTCCCCATTTTCATGGAGCTCTC-TGCAGATCT--------TGATGAATTTTTTATTTGTAGG--AGTTTGTAGGTTTTGAAGTTGGGAAAGTGCTTCTTCTTTTGTAAGAGA----GCAATTTGGATAAGTGAGGAAATAATTTTTGGAATTAATAAGAAAACGTTTCGGATGCATG---------TTGACC-AAATAGAGGAC---CCGATTGACCAG------CTCTT----ACAACTCTCCCCTGTATATTGGGTCT------CAA----------TATATA--GTGAG-ACCCAAATGGCATTG-TCGTAA--------TTTT-GGAAT-GAAAATC-----AAAATCCTCA----C------GCT--CCAAAAA--GC-GGCCAT--CCGTA-TAATATT

>CLCuAaV-PK:K804a:96aazAJ002452

-------ACCGGATGGCCGCGCGATTTTTTTTAG---TGGTGGGT-CCAG--AACGCAC-----------------------GACGATGCAGA--------CTC-----------AAAGCTTAGATAACG--------CTCCTTCGGCTATAAGTACTTGCGCACTAAGTTTCAA---ATTGAAAT-----------ATGTGGGATCCACT------AGTAAACGAATTCCCGGATACGGTGCACGGGTTTCGTTGTATGCATTCTGTC--------AAATATTTGCA-------ACTTTTGTCGCAGGATTA-TTCTCCAGATAC---CCTTGGTTACGAG--TTAATACGTGATTTAATCTGTATTCTACGCTCCCGTAATTATGTCGAAGCGAGCTGCCGATATCGTCATTTCTACGCCCGCGTCGAAAGTACGCCGGCGTCTGAACTTCGGCAGCCCATACACCAGCCGTGCTGCTGCCCCCATT---GTCCGCG-----------------TCACAAAACAACA---GGCATGGACAAACAGGCCTATGAACAGGAAACCCAGAATGTAC-CGGATGTACAGAAGTCCGGATGTTCC-AAGGGGATGT----GAGGGTCCCTGTAAGGTACAGTCGTTTGAATCTC-GACACGATGTCGTTCATATT-GGTA-AGGTAATGTGTATTTCGGATGTTACGCGTGGAGTCGGTTTGACCCATCGTATAGG---TAAGCGTTTTTGTGTCA-AGTCA----GTTTATGTTTTAGGTAAGATATGGATGGACGAGAACATCAAGACCAAGAACCATACGAATTCGGTGATGTTTTTCC-TTGTTCGTGATCGACGACCGGTAGATAAA---CCACAAGATTTTGGT-GAAGTATTTAATATGTTTGATAACGAGCCCAGCACGGCGACCGTGAAGAACATGCATAGGGATTGGTACCAGGT-GTTGAGGA---AATGGCATGCA-----ACCGTTACTGGTGGTCAATATGCGAGTAAGGAGCAGGCATTGGTCAAGAAG-TTTGTTAGGGTTA-ACAACTACGT-TGTTTACAACCAG--------CAG-GAAGCAGGAAAATACGAGAATCACACCGAGAATGCATTGATGCTTTATATGGCTTGTACCCATGCTAGTAACCCAGTTTATGCTACGCTTAAGATTAGAATATATTTTTATGACTCTG-------TAACGAACTAATA------------------TTAATAAAGTTTGAATTTTATATCTGAATATTGGTCTACATACAT-----TGTTTGATTAATTACATTGTACAATACATGTTCAACGGCTTTAATAACTAAATTAAGTGAGATTACACCTAGATTGTTGAGATACTTGAGTAC-------TTGGGTTTTGAAT---------------ACCCTTAAGAAAAGA-------------CCAGTCGGAG-GGTGTAAGGTC-----------GTCCAGATTC-GGAAGGTTAGAAAAC-ACTTGTGCACTCCC------AGAGCTTT----------CCGAAGGTTGTAGTTGAATTGGATCCTCATTGTTATGATGTCCATGTTC--------GTCGTGAATGGACGGTTGTCGTGGCTGAGGATTTTGAAATAAAGGGGATTTGGAACCTCCC--------AGATA------------AAGACGCCATTCGTTGAATGAGCT--GCAGTGATGCGTTCCCCTGTGCG--AGAATCCATGGTTGTGGCAGTTGATACTTAGGTAA-------TAGCTGCATCCACATTCAAGATC-CACTCTCCTCCTCC---TGGTCCTCCTCTTGGCCTCCCTGTGCTGGACTTTGA----TGGGCAATTGAGTACAGTGGTTCCTCGAGGGTGATGAAGAC---------------------------TGCATTCTTAACTGC------CCAGTTTTTTAGGGCTTGGTTCTTTTCCTCGTCCAGATATTCTTTATAAGA-TGAGTTTGGCCCAGGATTGCAGAGGAAGATT-------------------------------------GTGGGTATTCCACCTTTAA-TTTGAACTGGCTTCCCGTACTTTGTGTTTGATTGCCAGTCCCTT-----------------------TGGGCCCCCATGAACTCTTTAAAGTG--------CTTGAGGAAGTGCGGATCGACATCATCAATGACG-TTA----------------TACCACGCATC-ATTGCTGTAAAC---TTTGGGGCTTAAATCTAAATGACCGCATAAGTAATTATGAGGCCCTAATGACCTAGCCCACATTGTTTTACCCGT-------ACGACTGTCTCCCTCA-----------ATCACTATACTTTGAGGCCTCAGAGG-CCGCGCAGCGGCATCCATGACATTCTCGGAGACCCACTCTTCAAGTTCTTCAGGAACTTGATTAAAAGAAGAAGATAA------------------------------AAAAGGAGAAACATAAACCTCCAACGGAGGAGTGAAAAT-CCTATCTAAATTACT---ATTTAAATTATGATATTGAAAAATAAATTTTTCTGGGAGTTTTTCCCTTATTATAGCCAT---AGCTGCTTCTTTAGAACCTGCATTTAGGGCTTCTGCAGCAGCAT-CATTAGCTGTCTGTT----GACCTCCTCTTGCAGATCTTCCATCGATCTGAAATGTGCCCCAGTCGATGTAATCTCCGTCCTTCTCGATGTAGGACTTAACATCAGAGCTGGA---CTTAGCT-----CCCTGGAAGTTTGGATGGAATT--GGGTGGAGGTATGAGGGTGAGTGACATCGAAATGTCT-GGGGTTTCTGAACTTGGCTTTACCTTTGAATTGGAT-GAGTGCATGGATAT---GCAGAGACCCATCTTGGTGTTTTTCTTGTGCA-ACCC--------TAATGAATAATTTGTCTGAAGG--GCATGAAATGTTAGATAATATATCTAACATCTGTTCTTTTGGAATTGG----GCATTTTGGGAAAGTAAGAAAAATATTTTTTGCATTTATGCAAAAAGAATTATTTCTGGGC--ATT----TTTG---CTATCGGGGAC---ACTTAAAACTCA------TATCAATTGG------------GGACACTGGGGAC------TCA----------TTTATA--CTGCGTCCCCAAGTGGCATAA-ATGTAAATATTTGCCTTT-ATGTTTGAATTTC-----AAATTCCAAA----C------GCT--CCAAAAA--GC-GGCCAT--CCGTA-TAATATT

>CLCuAaV-PK:K802a:96aazAJ002455

-------ACCGGATGGCCGCGCGATTTTTTTTAG---TGGTGGGT-CCAG--AACGCAC-----------------------GACGATGCAGA--------CTC-----------AAAGCTTAGATAACG--------CTCCTTCGGCTATAAGTACTTGCGCACTAAGTTTCAA---ATTGAAAC-----------ATGTGGGATCCACT------AGTAAACGAATTCCCGGATACGGTGCACGGGTTTCGTTGTATGCTTTCTGTC--------AAATATTTGCA-------ACTTTTGTCGCAGGATTA-TTCTCCAGATAC---CCTTGGTTACGAG--TTAATACGTGATTTAATCTGTATTCTACGCTCCCGTAATTATGTCGAAGCGAGCTGCCGATATCGTCATTTCTACGCCCGCGTCGAAAGTACGCCGGCGTCTGAACTTCGGCAGCCCATACACCAGCCGTGCTGCTGCCCCCATT---GTCCGCG-----------------TCACAAAACAACA---GGCATGGACAAACAGGCCTATGAACAGGAAACCCAGAATGTAC-CGGATGTACAGAAGTCCGGATGTTCC-AAGGGGATGT----GAGGGTCCCTGTAAGGTACAGTCGTTTGAATCTC-GACACGATGTCGTTCATATT-GGTA-AGGTAATGTGTATTTCGGATGTTACGCGTGGAGTCGGTTTGACCCATCGTATAGG---TAAGCGTTTTTGTGTCA-AGTCA----GTTTATGTTTTAGGTAAGATATGGATGGACGAGAACATCAAGACCAAGAACCATACGAATTCGGTGATGTTTTTCC-TTGTTCGTGATCGACGACCGGTAGATAAA---CCACAAGATTTTGGT-GAAGTATTTAATATGTTTGATAACGAGCCCAGTACGGCGACCGTGAAGAACATGCATAGGGATCGGTACCAGGT-GTTGAGGA---AATGGCATGCA-----ACCGTTACTGGTGGTCAATATGCGAGTAAGGAGCAGGCATTGGTCAAGAAG-TTTGTTAGGGTTA-ACAACTACGT-TGTTTACAACCAG--------CAG-GAAGCAGGAAAATACGAGAATCACACCGAGAATGCATTGATGCTTTATATGGCTTGTACCCATGCTAGTAACCCAGTTTATGCTACGCTTAAGATTAGAATATATTTTTATGACTCTG-------TAACGAACTAATA------------------TTAATAAAGTTTGAATTTTATATCTGAATATTGGTCTACATACAT-----TGTTTGATTAATTACATTGTACAATACATGTTCAACGGCTTTAATAACTAAATTAAGTGAGATTACACCTAGATTGTTGAGATACTTGAGTAC-------TTGGGTTTTGAAT---------------ACCCTTAAGAAAAGA-------------CCAGTCGGAG-GGTATAAGGTC-----------GTCCAGATTC-GGAAGGTTAGAAAAC-ACTTGTGCACTCCC------AGAGCTTT----------CCGAAGGTTGTAGTTGAATTGGATCCTCATTGTTATGATGTCCATGTTC--------GTCGTGAATGGACGGTTGTCGTGGCTGAGGATTTTGAAATAAAGGGGATTTGGAACCTCCC--------AGATA------------AAGACGCCATTCGTTGAATGAGCT--GCAGTGATGCGTTCCCCTGTGCG--AGAATCCATGGTTGTGGCAGTTGATACTTAGGTAA-------TAGCTGCATCCACATTCAAGATC-CACTCTCCTCCTCC---TGGTCCTCCTCTTGGCCTCCCTGTGCTGGACTTTGA----TGGGCAATTGAGTACAGTGGTTCCTCGAGGGTGATGAAGAC---------------------------TGCATTCTTAACTGC------CCAGTTTTTTAGGGCTTGGTTCTTTTCCTCGTCCAGATATTCTTTATAAGA-TGAGTTTGGCCCAGGATTGCAGAGGAAGATT-------------------------------------GTGGGTATTCCACCTTTAA-TTTGAACTGGCTTCCCGTACTTTGTGTTTGATTGCCAGTCCCTT-----------------------TGGGCCCCCATGAACTCTTTAAAGTG--------CTTGAGGAAGTGCGGATCGACATCATCAATGACG-TTA----------------TACCACGCATC-ATTGCTGTAAAC---TTTGGGGCTTAAATCTAAATGACCGCATAAGTAATTATGAGGCCCTAATGACCTAGCCCACATCGTTTTACCCGT-------ACGACTGTCTCCCTCA-----------ATCACTATACTTTGAGGCCTCAGAGG-CCGCGCAGCGGCATCCATGACATTCTCGGAGACCCACTCTTCAAGTTCTTCAGGAACTTGATTAAAAGAAGAAGATAA------------------------------AAAAGGAGAAACATAAACCTCCAACGGAGGAGTGAAAAT-CCTATCTAAATTACT---ATTTAAATTATGATATTGAAAAATAAATTTTTCTGGGAGTTTTTCCCTTATTATAGCCAT---AGCTGCTTCTTTAGAACCTGCATTTAGGGCTTCTGCAGCAGCAT-CATTAGCTGTCTGTT----GACCTCCTCTTGCAGATCTTCCATCGATCTGAAATGTGCCCCAGTCGATGTAATCTCCGTCCTTCTCGATGTAGGACTTAACATCAGAGCTGGA---CTTAGCT-----CCCTGGAAGTTTGGATGGAATT--GGGTGGAGGTATGAGGGTGAGTGACATCGAAATGTCT-GGGGTTTCTGAACTTGGCTTTACCTTTGAATTGGAT-GAGTGCATGGATAT---GCAGAGACCCATCTTGGTGTTTTTCTTGTGCA-ACCC--------TAATGAATAATTTGTCTGAAGG--GCATGAAATGTTAGATAATATATCTAACATCTGTTCTTTTGGAATTGG----GCATTTTGGGAAAGTAAGAAAAATATTTTTTGCATTTATGCAAAAAGAATTATTTCTGGGC--ATT----TTTG---CTATCGGGGAC---ACTTAAAACTCA------TATCAATTGG------------GGACACTGGGGAC------TCA----------TTTATA--CTGCGTCCCTAAATGGCATAA-ATGTAAATATTTGCCTTT-ATGTTTGAATTTC-----AAATTCCAAA----C------GCT--CCAAAAA--GC-GGCCAT--CCGTA-TAATATT

>CLCuAaV-IN:Kar:OY77:05aazGU112081

-------ACCGGATGGCCGCGCGATTTTTTTAAG---TGGTGGGT-CCAG--AACGCAC-----------------------GACGATGCAGA--------CTC-----------AAAGCTTAGATAACG--------CTCCTTAGACTATCAGTACGTGCCCACTAAGTTTCAA---TTCAAAAA-----------ATGTGGGATCCACT------ATTAAACGAGTGCCCGGATACGGTTCACGGTTTTCGTTGTATGCTTTCTGTG--------AAATATTTGCA-------ACTTTTGTCGCAGGAGTA-TTCACCAGATAC---GCTTGGTTACGAT--TTAATACGGGATTTAATTTGTATTGTCCGTTCTCGTAATTATGTCGAAGCGAGCTGCAGATATCGTCATTTCTACGCCCGAATCGAAAGTACGCCGGCGTCTGAACTTCGGCAGCCCATACACCAGCCGTGCTGCTGCCCCCATT---GTCCGCG-----------------TCACAAAACAACA---GGCATGGACAAACAGGCCTATGAACAGGAAACCCAGAATGTAC-CGGATGTACAGAAGTCCGGATGTTCC-AAGGGGATGT----GAGGGTCCCTGTAAGGTACAGTCGTTTGAATCTC-GACACGATGTCGTTCATATT-GGTA-AGGTAATGTGTATTTCGGATGTTACGCGTGGAGTCGGTTTGACCCATCGTATAGG---TAAGCGTTTTTGTGTCA-AGTCA----GTTTATGTTTTAGGTAAGATCTGGATGGACGAGAACATCAAGTCCAAGAACCATACGAATTCGGTGATGTTTTTCC-TTGTTCGTGATCGACGACCGGTAGATAAA---CCACAAGATTTTGGT-GAAGTATTTAATATGTTTGATAACGAGCCCAGTACGGCGACCGTGAAGAACATGCATAGGGATCGGTACCAGGT-GTTGAGGA---AATGGCATGCA-----ACCGTTACTGGTGGACAATATGCGAGTAAGGAGCAGGCTTTGGTCAAGAAG-TTTGTTAGGGTTA-ATAACTACGT-TGTTTACAACCAG--------CAG-GAAGCAGGAAAATACGAGAATCACACCGAGAATGCATTGATGCTTTACATGGCTTGTACTCATGCTAGCAACCCAGTGTATGCTACGCTTAAGATTCGTATTTATTTTTATGACTCTG-------TAACGAACTAATA------------------TTAATAAAGTTTGAATTTTATATCTGAATATTGGTCTACATACAT-----TGTTTGATTAATTACATTGTACAATACATGTTCAACGGCTTTAATAACTAAATTAAGTGAGATTACACCTAGATTGTTGAGATACTTGAGGAC-------TTGGGTTTTGAAT---------------ACCCTTAAGAAAAGA-------------CCAGTCGGAG-GGTGTAAGGTC-----------GTCCAGATTC-GGAAGGTTAGAAAAC-ACTTGTGTATTCCC------AGAGCTTT----------CCGTAGGTTGTAGTTGAAATGGATCCTGAGTGTTATTATGTCCATGTTC--------GTCGTGAATGGACGGTTGTCGTGGTTGAGGATTTTGAAATAGAGGGGATTTGGAACCTTCC--------AGATA------------TAGACGCCATTCTTTGCTTGAGCT--GCAGTGATGAGTGCCCCTGTGCG--AGAATCCATGGTTGTGGCAGTTGATTGACAGATAA-------TAAGAACACCCGCATTCAAGATC-TACTCTCCTCCTCC---TGATGCGCCTCTTCGCTTCCCTGTGCTGTACTTTGA----TTGGTACCTGAGTACAGGGGTCCTTCAAGTGTGATGAAGAT---------------------------CGCATTCTTTACTGC------CCAGTTCTTTAGTGCGGTGTTCTTTTCCTCGTCTAGGAATTCTTTATAACT-GCTGTTGGGACCAGGATTGCAGAGGAAGATT-------------------------------------GTTGGTATCCCGCCTTTAA-TTTGAACTGGCTTCCCGTACTTTGTGTTGGATTGCCAGTCCCTT-----------------------TGGGCCCCCATGAACTCTTTAAAGTG--------TTTGAGGAAATGCGGGTCGACGTCATCAATGACA-TTA----------------TACCAGGCGTC-GTTACTGTAGAC---CTTGGGACTCAGGTCCAGATGTCCACACAAATAGTTATGTGGTCCCAGTGATCTAGCCCACATCGTCTTGCCGGT-------CTGACTGTCTCCCTCA-----------ATGTCCAAACTTTGAGGTCTCAGAGG-CCGCGCAGCGGCATCCATGACATTCTCGGAGACCCACTCTTCAAGTTCTTCAGGAACTTGATTAAAAGAAGAAGATAA------------------------------AAAAGGAGAAACATAAACCTCCAACGGAGGAGTGAAAAT-CCTATCTAAATTACT---ATTTAAATTATGATATTGAAAAATAAATTTTTCTGGGAGTTTTTCCCTTATTATAGCCAT---AGCTGCTTCTTTAGAACCTGCATTTAGGGCTTCTGCAGCAGCAT-CATTAGCTGTCTGTT----GACCTCCTCTTGCAGATCTTCCATCGATCTGAAATGTGCCCCAGTCGATGTAATCTCCGTCCTTCTCGATGTAGGACTTAACATCAGAGCTGGA---CTTAGCT-----CCCTGGAAGTTTGGATGGAATT--GGGTGGAGGTATGAGGGTGAGTGACATCGAAATGTCT-GGGGTTTCTGAACTTGGCTTTACCTTTGAATTGGAT-GAGTGCATGGATAT---GCAGAGACCCATCTTGGTGTTTTTCTTGTGCA-ACCC--------TAATGAATAATTTGTCTGAAGG--GCATGAAATGTTAGATAATATATCTAACATCTGTTCTTTTGGAATTGG----GCATTTTGGGAAAGTAAGAAAAATATTTTTTGCATTTATGCAAAAAGAATTATTTCTGGGC--ATT----TTTG---CTATCGGGGAC---ACTTAAAACTCA------TATCAATTGG------------GGACACTGGGGAC------TCA----------TTTATA--CTGCGTCCCTAAATGGCATAA-ATGTAAATATTTGCCTTT-ATGTTTGAATTTC-----AAATTCCAAA----C------GCT--CCAAAAA--GC-GGCCAT--CCGTA-TAATATT

>CLCuAaV-IN:Har:05aazGU112004

-------ACCGGATGGCCGCGCGATTTTTTTAAG---TGGTGGGT-CCAG--AACGCAC-----------------------GACGATGCAGA--------CTC-----------AAAGCTTAGATAACG--------CTCCTTCGGCTATAAGTACGTGCGCACTAAGTTTCAA---ATTGAAAC-----------ATGTGGGATCCACT------AGTAAACGAACT-CCGGATACGGTTCACGGGTTTCGTTATATGCTATCTGGTA-------AAATATCTGCA-------ACTTTTGTCGCTGGAGTA-TTCACCAGTTAC---GCTTGGGTACGAG--TTAATACGGGATTTAATTTGTATTGTACGCTCTCGTAATTATGTCGAAGCGAGCTGCCGATATCGTCATTTCTACGCCCGCGTCGAAAGTACGCCGGCGTCTGAACTTCGGCAGCGCATACACCAGCCGTGCTGCTGCCCCCATT---GTCCGCG-----------------TTACAAAACAACA---GGCATGGTCAAACAGGCCTATGAACAGGAAACCCAGAATGTAC-CGGATGTACAGAAGTCCGGATGTTCC-AAGGGGATGT----GAGGGTCCCTGTAAGGTACAGTCGTTTGAATCTC-GACACGATGTCGTTCATATT-GGTA-AGGTAATGTGTATTTCGGATGTTACGCGTGGAGTCGGTTTGACCCATCGTATAGG---TAAGCGTTTTTGTGTCA-AGTCA----GTTTATGTTTTAGGTAAGATATGGACGGACGAGAACATCAAGACCAAGAACCATACGAATTCGGTGATGTTTTTCC-TTGTTCGTGATCGACGACCGACAGATAAA---CCACAAGATTTTGGT-GAAGTATTTAATATGTTTGATAACGAGCCCAGTACGGCGACCGTTAAGAACATGCATAGGGATCGGTACCAGGT-GTTGAGTA---AATGGCATGCA-----ACCGTTACTGGTGGACAATATGCGAGTAAGGAACAGGCGTTGGTTAAGAAG-TTTGTTAGGGTTA-ACAATTATGT-TGTTTACAACCAG--------CAG-GAGGCAGGAAAATACGAGAATCACACGGAGAATGCGTTGATGCTTTATATGGCTTGTACCCATGCTAGCAACCCAGTTTATGCTACGCTTAAGATTCGGATATATTTTTATGACTCTG-------TAACGAATTGAAA------------------TTAATAAAGATTGAATTTTATATCTGAATATTGGTCTACATACAT-----TGTTTGATTAATTACATTGTACAATACATGTTCGACGGCTTTAATAACTAAATTAAGTGAGATTACACCTAGATTGTTGAGATATTTGAGGAC-------TTGGGTTTTGAAT---------------ACCCTTAAGAAAAGA-------------CCAGTCGGAG-GGTGTAAGGTC-----------GTCCAGATTC-GGAAGGTTAGAAAAC-ACTTGTGCACTCCC------AGAGCTTT----------CCGAAGGTTGTAGTTGAATTGGATCCTCATTGTTATGATGTCCATGTTC--------GTCGTGAATGGACGGTTGTCGTGGCTGAGGATTTTGAAATAAAGGGGATTTGGAACCTCCC--------AGATA------------AAGACGCCATTCGTTGAATGAGCT--GCAGTGATGCGTTCCCCTGTGCG--AGAATCCATGGTTGTGGCAGTTGATACTTAGGTAA-------TAGCTGCATCCACACTCAAGGTC-CACTCGTTTCCTCC---TGTGCGCTCTCTTCGCTTCCCTGTGTTGAACTTTGA----TTGGTACCGGAGTAGAGCGGTTGGGTGAGAAAGACGAATGT---------------------------TGCATTTTTTAAAGC------CCAGGATTTTAAGGCTGAGTTCTTATCCTCGTCTAAGAACTCTTTATAGCT-GGCGTTGGGTCCTGGATTGCAGAGGAAGATT-------------------------------------GTTGGTATGCCGCCTTTAA-TTTGAACTGGCTTCCCGTACTTTGTGTTTGATTGCCAGTCCCTT-----------------------TGGGCCCCCATGAACTCTTTAAAGTG--------TTTGAGGAAGTGCGGATCGACATCATCAATGACG-TTA----------------TACCACGCATC-ATTGCTGTAAAC---TTTGGGGCTTAAATCTAAATGACCACATAAGTAATTATGTGGCCCTAACGACCTAGCCCACATTGTTTTTCCAGT-------ACGACTATCTCCCTCA-----------ATTACTATACTTTGAGGTCTCAGGGG-CCGCCCAGCGGCATCGACAACGTTCTCGCACGCCCACTCTTCAAGTTCTTCTGGAACTTGATCAAATGAAGAAGAAGA------------------------------AAAAGGAGAAACATAAGGAGCTGGAGGCTCC-TGAAAATATCTATCTAAATTAGA---ATTTAAATTATGAAATTGAAGTAGAAAGTCTCTTGGGGCTAACTCCCTAAGGACT-TTGAG--GGCCTGAGCTTTGGACCCTGCGTTGATTGCCTCGGCATATGCGT-CGTTGGCAGTTTGGC----AACCTCCTCTAGCTGATCTTCCATCGACTTGGAAAACTCCATGATCAAGGATGTCTCCGTCTTTCTCCATGTAGGTTTTGACATCGGAGCTGGA---TTTAGCT-----CCCTGGAAGTTTGGATGGAATT--GGGTGGAGGTATGAGGGTGAGTGACATCGAAATGTCT-GGGGTTTCTGAACTTGGCTTTACCTTTGAATTGGAT-GAGTGCATGGATAT---GCAGAGACCCATCTTGGTGTTTTTCTTGTGCA-ACCC--------TAATGAATAATTTGTCTGAAGG--GCATGAAATGTTAGATAATATATCTAACATCTGTTCTTTTGGAATTGG----GCATTTTGGGAAAGTAAGAAAAATATTTTTTGCATTTATGCAAAAAGAATTATTTCTGGGC--ATT----TTTG---CTATCGGGGAC---ACTTAAAACTCA------TATCAATTGG------------GGACACTGGGGAC------TCA----------TTTATA--CTGCGTCCCTAAATGGCATAA-ATGTAAATATTTGCCTTT-ATGTTTGAATTTC-----AAATTCCAAA----C------GCT--CCAAAAA--GC-GGCCAT--CCGTA-TAATATT

>ChLCPKV-PK:Mul:04aazDQ116877

-------ACCGGATGGCCGCGA--TTTTTTT------TCAGTGGTCCCACCACACGCACTAAAGGACA----TTTACACGTGGACCAATTAAA-ACATGTCCTC-----------AAAGCTTAATTGTTT-C-GTGGTCCCCTA----TTTAA--ACTTGGGCTCCAAGTAGTGC---ACTCATACCA---------ATGTGGGATCCATT------AGTAAACGAGTTTCCCGAAACCGTTCACGGTTTTAGGTGTATGCTAGCAGTG--------AAATATCTCCA-------GCTTGTAGAAAAGACGTA-TTCGCCCGATAC---TCTTGGGTACGAT--TTAATTAGGGATTTAATTTCAGTCATCAGGGCTAGAAATTATGTCGAAGCGACCAGCAGATATAATCATTTCCACGCCCGTTTCGAAGGTACGCCGACGTCTCAACTTCGACAGCCCATATGCGAGCCGTGCCACTGCCCCCATT---GTCCGCG-----------------TCACCAAAGCAAA---AGCATGGGCGAACAGGCCCATGAACAGAAAGCCCAGGATGTAC-AGGATGTTCCGAAGTCCGGATGTTCC-TAGAGGATGT----GAAGGCCCATGTAAGGTCCAGTCCTTTGAGTCTA-GACATGACATTCAACATATA-GGTA-AAGTTATGTGTATCAGTGATGTTACTCGTGGAACTGGGTTGACCCATCGAGTGGG---TAAGAGGTTTTGTGTTA-AATCC----GTTTATGTCTTGGGTAAGATATGGATGGATGAAAATATTAAGACCAAGAATCACACGAACAGTGTGATGTTTTTTC-TAGTTCGGGATCGTAGACCTGTGGATAAA---CCTCAAGACTTTGGC-GAGGTGTTTAACATGTTTGATAATGAGCCCAGTACGGCTACTGCGAAGAATGTTCATCGTGATAGGTATCAAGT-TCTTCGGA---AATGGCATGCA-----ACAGTTACCGGTGGACAGTATGCGTCTAAGGAACAAGCTCTTGTGAAGAAG-TTTGTGAGGGTCA-ATAATTATGT-TGTGTATAACCAG--------CAA-GAAGCTGGAAAGTATGAAAATCATTCTGAGAATGCGTTAATGTTGTATATGGCGTGTACTCATGCCTCTAACCCTGTGTATGCTACTTTGAAGATACGGATCTATTTTTATGATTCCG-------TAACAAA------------------------TTAATAAATGTTAAACTTTATTGAATAAGATTGTTGTACATATAC-----AATGTGTTGTATTACATTCCATAATACATGATCAACCGCACGATTTACATTATTAATACTGACAACTCCTAAACGATCTAAATATTTAATAAC-------CTGAGTCTTAAAG---------------ACCCTCAAGAAACGC-------------CCAGTCTGAG-GCTGTGAAGTC-----------ATCCAGATTC-GGTAGACTAGAAAAC-ACTTGTGCACTCCC------AGAGCTTT----------CCTCAGGTTGTGATTGAACTGTATTCGGACGGTGATTATGTCTTGGTTC--------ATGAGGAATGGACGGTTCCGGTGCTCTATTATCTTGAAATACAGGGGATTTTGAATCTCCC--------AGATA------------AACACGCCATTCTCTGCTTGAGCT--GCAGTGATGGGTTCCCCTGTGCG--TGAATCCATAGCCGTGGCAGCGTAATGCTATGAAA-------TATGAACAGCCGCAGTCTAGGTC-AACGCGTCGTCGCC---TGATTCCCCTCTTGGCTTGCCTGTGCTGCACTTTGA----TTGGAACCTGAGTAGAGTGGGCCTTGGAGGGTGATGAAGGT---------------------------CGCATTCTTTAAAGC------CCAACTTTTCAGCGCACTATTCTTCTCTTCATCCAAGAACTCTTTATAGCT-AGAGTTGGGTCCTGGATTGCAGAGGAAGATA-------------------------------------GCGGGAATTCCGCCTTTAA-TTTGAACTGGCTTTCCGTATTTCGTATTGCTTTGCCAGTCCCTT-----------------------TGGGCCCCCATGAATTCCTTAAAGTG--------CTTTAGATAATGCGGATCAACGTCATCAATGATG-TTG----------------TACCATGCGTC-GTTGCTGTAGAC---CTTAGGGCTAAGGTCTAGATGACCGCACAAATAATTGTGTGGGCCCAAAGACCTAGCCCACATGGTCTTTCCAGT-------CCTAGAAGCACCCTCC-----------ACCACAATACTCATGGGTCTCAACGG-CCGCGCAGCGGCATCAACCACATTCTCCGACCTCCACTCCTCAAGTTCTTCCGGAACTTGATCAAAAGAAGAAGAACA------------------------------ACAAGGAGAAACATAAACCTCCACCGGAGGTGTAAAAAT-CCTATCTAAATTACT---TTTTAAATTATGATATTGAAAAATAAAATCTTTTGGGAGCTTTTCCCTTATTATTGCTAACGCAGCTTCAGCCGAA---CCTGCATTTAAGGCCTCTGCGGCAGCAT-CATTAGCTGTCTGTT----GACCTCCTCTAGCAGATCGTCCATCGATCTGAAACCGACCCCAGTCGATGTAATCACCGTCCTTCTCGATGTAGGACTTGACATCAGAGCTGGA---CTTAGCT-----CCCTGGAAGTTTGGGTGGAATT--GGGTGGTGTTATTAGGGTGAGTGACATCGAAATGTCT-AGGGTTTCGGAACTGGGATTTACCTTTGAATTGGAT-GAGGGCATGGATAT---GCAAGTTCCCATCTTGGTGTTTTTCCTGTG-ATACTC--------TGATAAATAATTTATCAGAAGGACAATTTATAGATTTGAGAATT--TCTAGCATTTGTTCTTTGGGTATTGG----GCATTTTGGATAAGTTAGGAAGATATTTTTTGCCTTAACTTGGAATTGGTGTTGACGTGGCATATTGTA-TTGG----------GTGC---TCTTCAAAACCC------TATGGAATGGG-----------GTGCTTTGGGTGC------CTA----------TTTATA--CGGAGCTCCCAAATGGCATTG-ACGTAA--------TTTC-GTGGA-AAATTCC-----AAAATTTTCC----C------GCT--CGAAAAATCGC-GGCCAT--CCGTA-TAATATT

>ChLCPKV-IN:PRM:05aazDQ629103

-------ACCGGATGGCCGCGATTTTTTTTA------CCGTGGGCCCCAC--TACGCACGTGCTGACA----AAGACATGTGCACCAATTAAA-ATCGTCCCTC-----------ATAGCTTAATTATTT-C-ATGGTCCCCCC----TATAA--ACTTGGGCTCCAAGT----A---GTGCACTCTT-----ACCAATGTGGGATCCATT------AGTAAACGAGTTTCCTGAAACCGTTCACGGTTTTAGGTGTATGTTAGCAGTT--------AAATATCTGCA-------GCTACTAGAAAATACGTA-TTCTCCAGACAC---TCTGGGGTACGAT--TTAATCAGGGATTTGATCTCCGTTATTAGGGCTAAGAATTATGTCCAAGCGACCAGCAGATATAATCATTTCCACGCCCGCCTCGAAGGTACGCCGCCGTCTCAACTTCGACAGCCCTTATGCCAGCCGTGCTGCTGCCCCCACT---GTCCGCG-----------------TCACAAAGGCCAG---AGCATGGGTGAACAGGCCCATGAACAGGAAGCCCAGGATGTAC-AGGATGTACAGAAGCCCAGATGTTCC-TAGGGGGTGT----GAAGGCCCATGTAACGTCCAGTCTTTTGAGTCTA-GACACGATGTAGTTCATATA-GGGA-AGGTTATGTGTATTAGTGATGTTACCCGTGGTACTGGGTTAACCCATAGAGTAGG---TAAGCGTTTCTGTGTTA-AGTCT----GTGTACGTATTACGTGAGATATGGATGGATGAGAATATCAAGACTAAAGATCACACGAATAGTGTGATGTTTTCCC-TTGTCCGTGATCGTCGTCCTGTTGATAAA---CCACAAGATTTTGGA-GAGGTGTTCAACATGTTTGACAACGAGCCTAGCACTGCCACTGTGAAGAATATGCATAGAGATCGTTATCAGGT-TTTGAGAA---AATGGCACGCA-----ACGGTTACTGGTGGACAGTACGCGTCGAAGGAACAGGCATTAGTTAAGAAG-TTCGTTAGGGTTA-ATAATTATGT-TGTGTATAACCAG--------CAA-GAAGCTGGTAAATATGAGAATCATACTGAGAATGCATTGATGTTGTACATGGCGTGTACTCACGCCTCTAATCCCGTGTATGCTATTTTAAAGATACGGATCTATTTCTATGATTCAG-------TATCGAA------------------------TTAATAAAGATTGAATTTTATTATATTTGAACTTTGTACATGAAT-----TGTTTGTGCTAATACATTCCATAATACATGGTTGACAGCACTAAGTACATTGTTTATACTAATTACAGCAAAATTATTTAAATACTGCAACAC-------TTGGGTCCTAAAT---------------ACCCTTAAGAAATGA-------------CCAGTCTGAG-GCTGTAAGGTC-----------GTCCAGATTC-GGAAGGTTAGAAAAC-ATTTGTGTATCCCC------AACGCTTT----------CCTCAGGTTGTGATTGAACCGTATCTGCACGGTGATGATGTCGTGGTTC--------CTCAGGAATGGCCGGTTGTGGTGCTCTGTTATCTTGAAATATAGGGGATTTGTTATCTCCC--------AGATA------------AACACGGAATTCTCTGCTTGAGCT--GCAGTGATGGGTTCCCCTGTGCG--TGAATCCATAGCCGTGGCAGCGTAATGCGATGAAA-------TATGAACAACCGCAGTCTAGGTC-AACCCGACGACGCC---TGGTCCCCTTCTTGGCCAGCCTGTGCTGCACTTTGA----TTGGAACCTGAGTAGAGTGGGCCTTCGAGGGTGATGAAGGT---------------------------CGCATTCTTTAAAGC------CCAATTTTTGAGTGCGCTATTTTTCTCTTCATCCAAGAACTCGTTATAGCT-GGAATTGGGTCCTGGATTGCAGAGGAAGATA-------------------------------------GCGGGAATTCCACCTTTAA-TTTGAACTGGCTTTCCGTATTTAGTGTTGGATTGCCAGTCCTTC-----------------------TGGGCCCCCATGAATTCTTTAAAGTC--------CTTTAGGTAGTGGGGGTCGACGTCATCAATGACG-TTA----------------TACCAGGCGTC-ATTACTGTAGAC---CTTTGGGCTAAGGTCGAGATGTCCACACAAATAGTTGTGTGGACCTAGTGACCTGGCCCACATTGTCTTGCCTGT-------TCTACTATCGCCCTCT-----------ATCACTATACTGATGGGTCTCAATGG-CCGCGCAGCGGCACCGAGAACATTCTCGGCAGCCCATTCTTCAAGTTCCTCCGGAACTTGATCGAAAGAAGAAGAACA------------------------------AAAAGGAGAAACATAAACCTCCAACGGAGGCGTAAAAAT-CCTATCTAAATTTGC---ACTTAAATTATGATATTGTAATACAAAATCTTTGGGAGCTTTCTCCCTTAATATA-TTGAG--GGCCGCAGCTTTGGACCCTGAATTGATTGCCCCGGCATATGCGT-CGTTGGCAGACTGCT----GACCTCCTCTAGCTGATCTTCCATCGACTTGGAAAACTCCAAAATCAATGAAGTCTCCGTTTTTTTCCACATAGGCCTTGACATCTGA---CGAGCTTTTAGCT-----CCCTGAATGTTCGGATGGAAAT--GTGCTGGCCCTGATGGGGAAACGAGGTCGAAGAATCT-GTTGTTTTGGCATTTGAATTTACCTTCGAATTGTAT-GAGGACGTGGATAT---GAGGTTCCCCATTTTCATGTAATTCGC-GACAAACCC--------TGATGAACAATTTATTAGTGGG--GGTGCTTAGGTTTTGAAGTTGGGAAAGTGCTTCTTCTTTAGTGAGGGA----ACACTGTGGATATGTTAAGAAATAATTTTTAGCATATATTTGAAAACGTTTTGGAGGAGCCATGTTGAC-TTGGT--CAATCG-GTAC---TCAACAAACTTG------GCTAT----GCAA---TC----GGTGAATGGTACT------CAC----------TATATAGTGTGAG-TACCAAATGGCATGT-TCGTAA--------TTTT-GTAAG-CAAATTC-----AAAATTTAAT-----------TCTTACCAAAAA--GCGGGCCAT--CCGCACTAATATT

>ACMV-PK:Mul:Sto:08GQ204109

-------ACCGGTTGGCCCCGCCCCCCTTTAA-----ACGTGGTCCCCGC-----GCACTATGT-------------ATGTCGGCCAATCATGTCGTAGCGTT------------AAAGGTTATTTATTAGTGGTGGGCCAC------TATAT--ACTTGCAGGCGAAGT---CG---TTGCTAGTGCGCA------ATGTGGGATCCACT------GGTGAATGAGTTTCCTGACTCGGTGCATGGGCTTAGGTGTATGCTTGCAATT--------AAATATTTGCA-------GGCCTTAGAGGATACATA-CGAGCCCAGTAC---TCTGGGCCACGAT--CTGGTTAGAGATTTAGTCTCAGTTATCAGGGCTCGTAATTATGTCGAAGCGACCAGGAGATATCATCATTTCCACTCCAGGCTCCAAGGTTCGTCGAAGGCTGAACTTCGACAGCCCATACAGGAACCGTGCTACTGCCCCCACT---GTCCACG-----------------TCACAAATCGAAAACGGGCCTGGATGAACAGGCCCATGTACAGAAAGCCCATGATGTAC-AGGATGTATAGAAGCCCAGACATACC-CAGGGGCTGT----GAAGGCCCATGTAAGGTCCAGTCGTTTGAGCAGA-GGGATGATGTTAAGCACCTT-GGTATCTGTAAGGTG-ATTAGTGATGTGACACGTGGGCCTGGGCTGACACACAGGGTCGG---AAAGAGGTTTTGTATCA-AGTCC----GTTTACATTCTTGGCAAGATCTGGATGGATGAAAATATTAAGAAGCAGAATCACACTAATAATGTGATGTTTTACC-TGCTTAGGGATAGAAGGCCTTATGGCAATACGCCCCAAGACTTTGGG-CAGATATTTAACATGTTTGATAATGAGCCCAGTACTGCAACAATTAAGAACGATTTGAGGGATAGGTTTCAGGT-GTTGAGGA---AATTTCATGCC-----ACTGTTATTGGTGGTCCATCTGGCATGAAGGAGCAGGCTTTGGTGAAAAGG-TTTTACAGGTTGA-ATCATCACGT-GACATATAATCAT--------CAG-GAGGCAGGGAAGTATGAGAATCACACAGAGAATGCGTTGCTTTTGTACATGGCATGTACTCATGCCTCCAATCCTGTATATGCTACGTTGAAAATACGTATATACTTCTATGACAGTA------TTGGCAA-------------------------TTAATAAATATTGAATTTTATT-----TCATGAGTCAACTGACACGCAATAGTTTTTTCAATTACATTGAACAAAACATGATCAGCAGCTCTAATTACATGGTTAATTGAGATAACACCTATATTATCCAAGTATTTAAGTAC-------TTGGTATCTAAAG---------------ACCCTTAAGAAAAGA-------------CCAGTCTGAG-GCTGTAAGGTC-----------GTCCAGATCC-TGAAGTTGAGAAAAC-ATTTGTGAATCCCC------AGCTCCTT----------CCGCAGGTTGTGATTGAATCGAACCTGGACTGTTATGATGTCCTGGTTC--------AGCAGGAATGGTCGTTGTTGGTGCCTGGTTATTGTGAAATACAGGGGATTGTTTATTTCCC--------AGGTA------------TACACGCCATTCATTGCTTGAGGA--GCAGTGATGAGTTCCCCTGTGCG--CAAATCCATGATTGGAGCAGTTGATATGGAGGTAA-------TATGAACAGCCACAGACAAGATC-CACTCTCCTACGCCGGATGGCTCGCTTCTTGAATTGTCTGTGACTGACTTTGA----TTGGAACGTGAGTAGAGTGGTTCTGTGAGGGTGATGAAGAT---------------------------TGCATTCTTTAATGC------CCAGGCCTTTAGCGCTTCTTGCTTTTCCTCGTCTAGGAACTCTTTATAGGA-CGAGGTTGGTCCTGGATTGCAGAGGAAGATA-------------------------------------GTGGGAATGCCACCTTTAA-TTTGAGCGGGTTTCCCGTATTTTGTGTTGGACTGCCAGTCCCGC-----------------------TGGGACCCCATGAATTCCTTAAAGTG--------CTTTAGGTAGTGTGGATCGACGTCATCAATGACG-TTG----------------TACCAAGCAGC-ATTATTGAAGAC---CTTTGGACTAAGGTCCAGGTGTCCACACAGGTAATTGTGTGGGCCTAAAGATCTGGCCCATATCGTCTTCCCTGT-------TCTGCTATCACCTTCT-----------ATAACAATACTATTGGGTCTCCATGG-CCGCGCAGCGGAAGCCCTAACATTATCAGCGACCCATTCTTCAAGTTCATCAGGAACTTGGTCAAAGGAAGAACATGG------------------------------GAAGGGAGAAATATAAGGAGCTGGTGGCTCCTGGAAAAT-CCTTTCTAAATTACT---ATTTAGATTATGAAACTGAAGTACAAAGTCCTTTGGGACTAATTCCCTAATGACA-TTAAG--AGCTTCTGACTTACTGCCGCTGTTAAGCGCTTTGGCGTAAGCAT-CATTTGCGGACTGTT----GACCTCCTCTAGCAGATCGTCCATCGATCTGAAATCGTCCCCATTCGACGGTGTCGCCGTCCTTATCCAGGTATGACTTGACATCTGAGCTTGA---TTTGGCA-----CCTTGAATGTTGGGGTGGAAACTGGTGCTA--CAGCTTGGGTGTAGACAATCGAAGAGACG-ATTGTTCGTAATCGTGATTTTGCCCTCGAATTGGAT-GAGGGCATGCAAGT---GAGGTTCCCCATTCTGATGCAGCTCTC-TACAGATTT--------TAATGAACTTAGGGTTAGATGG--GAGAGAGAGTGTTTGAATGAATGACAGCAGGTGTTCTTTGGGGATAGA----ACACTTTGGGTATGTGAGAAAGACATTCTTGGCTTGAACTCTAAAACG---AGGAGTCCTCATTTTGAC-CAAGT--CAATTG-GAGA----CACTCAACTAGAGACACTCTTGAGCATC-----TCCTCCTGTTAATTGGAGA------CAT----------TATATA-GGTGT--CTCTAAATGGCATTC-TTGTAA--------TAAGTTAAACTTTAATTTGAATGAAAAGCCTCAAAAGGCGCATAACA--CCCAAGG-----GGCCAA--CCGTA-TAATATT
